# Estimating SARS-CoV-2 infections from deaths, confirmed cases, tests, and random surveys

**DOI:** 10.1073/pnas.2103272118

**Published:** 2021-07-26

**Authors:** Nicholas J. Irons, Adrian E. Raftery

**Affiliations:** ^a^Department of Statistics, University of Washington, Seattle, WA 98195;; ^b^Department of Sociology, University of Washington, Seattle, WA 98195

**Keywords:** SARS-CoV-2 incidence, coronavirus infections, Bayesian estimation, United States COVID data

## Abstract

There are multiple sources of data giving information about the number of SARS-CoV-2 infections in the population, but all have major drawbacks, including biases and delayed reporting. For example, the number of confirmed cases largely underestimates the number of infections, and deaths lag infections substantially, while test positivity rates tend to greatly overestimate prevalence. Representative random prevalence surveys, the only putatively unbiased source, are sparse in time and space, and the results can come with big delays. Reliable estimates of population prevalence are necessary for understanding the spread of the virus and the effectiveness of mitigation strategies. We develop a simple Bayesian framework to estimate viral prevalence by combining several of the main available data sources. It is based on a discrete-time Susceptible–Infected–Removed (SIR) model with time-varying reproductive parameter. Our model includes likelihood components that incorporate data on deaths due to the virus, confirmed cases, and the number of tests administered on each day. We anchor our inference with data from random-sample testing surveys in Indiana and Ohio. We use the results from these two states to calibrate the model on positive test counts and proceed to estimate the infection fatality rate and the number of new infections on each day in each state in the United States. We estimate the extent to which reported COVID cases have underestimated true infection counts, which was large, especially in the first months of the pandemic. We explore the implications of our results for progress toward herd immunity.

## Significance

The novel coronavirus SARS-CoV-2 has infected over 33 million people in the United States. Nationwide, over 600,000 have died in the COVID-19 pandemic, which has necessitated shutdowns of schools and sectors of the economy. The extent of the virus’ spread remains uncertain due to biases in test data. We combine multiple data sources to estimate the true number of infections in all US states. These data include representative random testing surveys from Indiana and Ohio, which provide potentially unbiased prevalence estimates. We find that approximately 60% of infections have gone unreported. Even so, only about 20% of the United States had been infected as of early March 2021, suggesting that the country was far from herd immunity at that point.

SARS-CoV-2 test data are fraught with biases that obscure the true rate of infection in the population. Lack of access to viral tests, which was particularly pronounced in the early days of the pandemic, in conjunction with selection bias due to asymptomatic and mild infections, yield case counts that tend to underestimate the true number of infections in the population. By the same token, test positivity rates tend to overestimate viral prevalence. Hospitalization rates and emergency room visits do not estimate the overall infection rate and are not comparable between states or counties, or over time. Reported deaths due to COVID are considered less problematic as an estimate of the true death count and provide a more accurate reflection of the course of the pandemic (1).

We combine several of the main sources of data relevant to the number of infections using a simple Bayesian model that accounts for the biases and delays in the data. Our model relies on data on deaths due to COVID, confirmed cases, and testing reported by the COVID Tracking Project (2). We use a modified Susceptible–Infected–Removed (SIR) model, a compartmental epidemiological model widely used to simulate the spread of disease in a population. We combine this with a Poisson likelihood for death counts and a normal likelihood for estimates of viral and seroprevalence from random-sample testing surveys conducted in Indiana and Ohio (3, 4).

With these data, we infer the infection fatality rate (IFR) and obtain statistically principled estimates of the number of new infections on each day since March 2020 in Indiana and Ohio. We then leverage our results from these states to build a model for confirmed cases that accounts for preferential testing as a function of the cumulative number of tests administered in each state. This allows us to pin down the IFR and infection counts for the vast majority of states that have not conducted representative testing surveys.

Our simple Bayesian model takes inspiration from Johndrow et al. (5), although it differs in significant ways. Whereas Johndrow et al. model the effect of social distancing measures by allowing the SIR contact parameter to change prelockdown and postlockdown, we allow it to vary in time to account for fluctuation in the tightening and loosening of restrictions, as well as in adherence to the restrictions. Furthermore, we incorporate testing data, develop a statistical model for preferential testing, and include the IFR as a parameter in the model to be estimated, rather than a fixed constant. Finally, to simplify model implementation, we use a discrete-time SIR model, rather than a continuous-time model based on differential equations.

## Results

Here, we present detailed results for Indiana and Ohio, two states with statewide representative random-sample testing surveys that we incorporate into our probabilistic model. In order to assess the accuracy of our estimates, we also present results for Connecticut and New York. Connecticut has conducted a statewide representative seroprevalence survey (6) and was also included in a nonrandom seroprevalence study of 10 sites across the country (7). New York has the highest number of reported deaths due to COVID, and there is a body of literature studying the spread of the disease in the state, including refs. 8–10. The estimates from these studies provide a basis of comparison for our results.

We also present aggregated estimates for the entire United States. *SI Appendix*, Table S1 includes estimates of the IFR and the cumulative incidence (i.e., the percent of the state’s population having been infected) and undercount factor for all 50 states and the District of Columbia (DC) as of March 7, 2021, the last day reported by the COVID Tracking Project. Plots for the 50 states and DC are also shown in *SI Appendix*. We have created an online dashboard where updated results can be found, including estimated daily infections, the IFR, and the reproductive number r(t) in each state (11).

### Indiana.

**Fig. 1.**
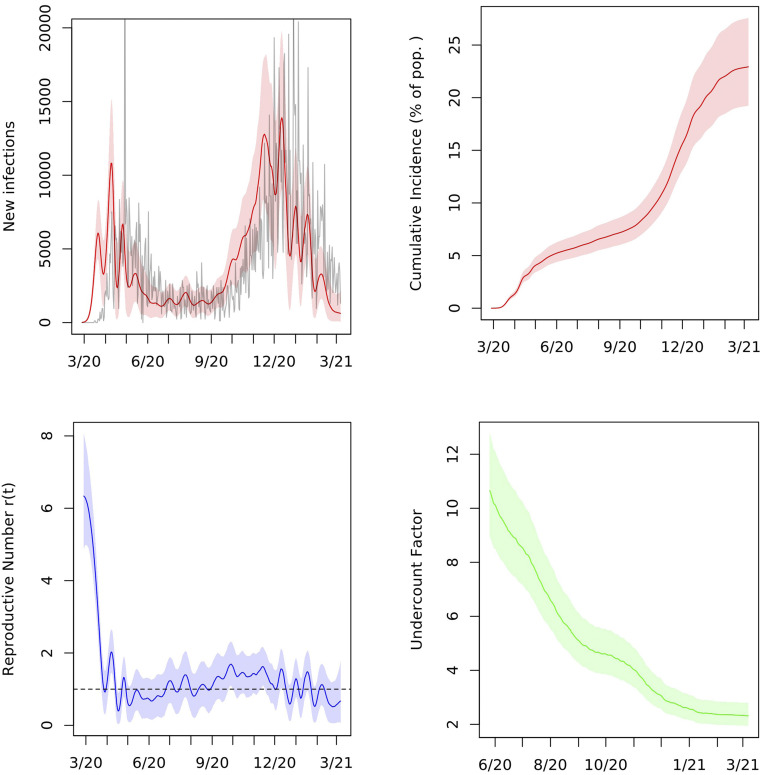
Posterior median and middle 95% intervals for daily new infections, cumulative incidence, reproductive number r(t), and cumulative undercount in Indiana from March 2020 to March 2021. In *Upper Left*, deaths divided by the posterior median IFR are plotted in gray for comparison. Pop., population.

We estimate an IFR of 0.84% (95% interval 0.70 to 1.00) in Indiana. We estimate the cumulative incidence of COVID-19 in the state at 19.7% (16.5 to 23.7) as of January 1, 2021, and 20.9% (17.5 to 25.1) as of January 15, 2021. By March 7, 2021, cumulative incidence had increased to 22.9% (19.2 to 27.6), nearly a quarter of the state, or about 1.5 million infections. There have been 2.3 (1.9 to 2.8) infections for every confirmed case in the state through this date. This suggests that a large majority of infections in the course of the pandemic have gone unreported, although Fig. 1 shows that undercounting was most pronounced early on and has improved substantially over time.

Fig. 1 exhibits posterior estimates of new infections on each day, $\nu_{t}$, as well as the cumulative undercount factor, which is the ratio of estimated cumulative infections to cumulative confirmed cases. Fig. 1 displays the viral prevalence, the cumulative incidence, and the reproductive number $r\left(t\right)=\beta_{t}/\gamma$ on each day.

By the time that the first confirmed case was reported in Indiana on March 6, 2020, there had likely been more than 800 infections in the state (95% interval 483 to 1,384). We estimate that as of May 1, 2020, there were 274,000 cumulative infections (95% interval 230,000 to 327,000), compared to 18,630 confirmed cases by that date. This yields a cumulative incidence of 4.1% (3.4 to 4.8) and an undercount factor of 14.7 (12.4 to 17.6). This estimate is comparable to others in the literature for that period (5, 7, 12). Between March 16 and March 19, 2020, the state’s Governor Eric Holcomb ordered a stop to indoor dining, declared a state of emergency, and closed schools; on March 23, 2020, he issued a stay-at-home order. According to our model, the first wave of infections reached its peak about 2 wk later in early April 2020.

### Ohio.

We estimate an IFR of 0.83% (95% interval 0.68 to 1.03) in Ohio. As of March 7, 2021, the cumulative incidence in the state was 19.5% (15.9 to 23.7), and the cumulative undercount factor was 2.3 (1.9 to 2.9).

**Fig. 2.**
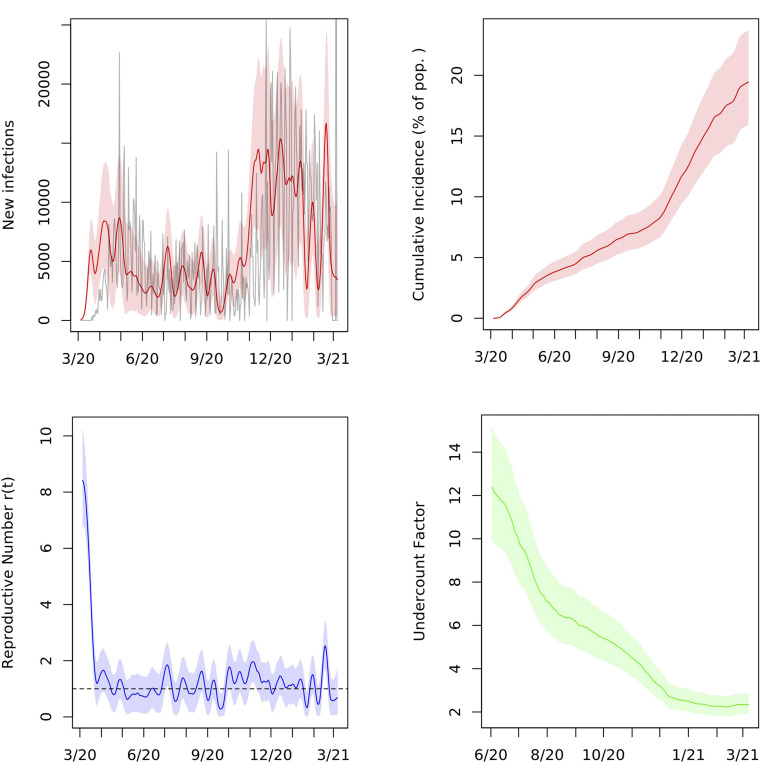
Posterior median and middle 95% intervals for daily new infections, cumulative incidence, reproductive number r(t), and cumulative undercount in Ohio from March 2020 to March 2021. In *Upper Left*, deaths divided by the posterior median IFR are plotted in gray for comparison. Pop., population.

Ohio Governor Mike Dewine declared a state of emergency on March 9, 2020, and the state’s first stay-at-home order took effect on March 23, 2020. In mid-April, the governor declared that businesses could begin to reopen on May 1. Fig. 2 shows that the first wave of infections, which picked up in March 2020 and likely peaked by late April 2020, did not die out, but, rather, leveled out to a sustained spread through the summer of 2020. The posterior median of the reproductive number r(t) in the state hovered around one from early April through mid-September and increased thereafter as the second wave of infections began in the fall.

### Connecticut.

We estimate an IFR of 1.37% (95% interval 1.10 to 1.70) in Connecticut. As of March 7, 2021, 15.9% (12.9 to 19.9) of the state’s population had been infected, leading to an undercount factor of 2.0 (1.6 to 2.5).

According to our model, as of April 26, 2020, 5.7% (4.6 to 7.1) of the state’s population had recovered from COVID. In comparison, Havers et al. (7) estimated a seroprevalence of 4.9% (95% interval 3.6 to 6.5) in the state in the period April 26 to May 3. Their study relied on a convenience sample of residual blood specimens collected for clinical purposes, and so it may have been affected by selection bias, as well as imperfect sensitivity and specificity of the antibody test used. Nevertheless, their estimate agrees well with the result from our model.

By July 5, 2020, our estimate of the recovered population increased to 8.9% (7.2 to 11.1). By comparison, in a random-sample blood test survey, Mahajan et al. (6) reported a seroprevalence of 4.0% (90% interval 2.0 to 6.0) for the period June 10 to July 29, which is significantly lower. While our estimates disagree with those of Mahajan et al., we note that the survey response rate was low at 7.8%, raising the possibility of significant nonresponse bias. For this reason, we did not include the Connecticut survey as a source of data in our analysis.

### New York.

We estimate an IFR of 1.12% (95% interval 0.87 to 1.42) for New York state. As of March 7, 2021, 18.6% (14.7 to 23.9) of the state had been infected, yielding an undercount factor of 2.1 (1.7 to 2.8) through that date.

We know of no other estimates of the IFR in New York in the literature. However, Yang et al. (8) estimated an IFR of 1.39% (95% interval 1.04 to 1.77) for the first wave in New York City (NYC) through June 6, 2020, based on available testing, mortality, and mobility data. According to NYC Health Department data (10), this period accounted for more than 85% of COVID deaths in the city and 57% of all confirmed COVID deaths (not including probable deaths) in the state through the first week of January 2021. As such, we expect the IFR for the state as a whole to have been similar to that of NYC during the spring of 2020, and our results are consistent with those of Yang et al.

We estimate that by June 6, 11.5% of the state’s population (95% interval 9.0 to 14.7), or about 2.2 million people, had been infected with the novel coronavirus. Multiplying that number by the fraction of confirmed COVID deaths in the state occurring in NYC during that period yields 1.7 million infections, or 20% of the city’s population. This number matches that of Stadlbauer et al. (9), who measured 20% seroprevalence in NYC at that time based on randomly sampled residual plasma collected from patients at Mount Sinai Hospital scheduled for routine care visits unrelated to COVID-19.

### United States.

**Fig. 3.**
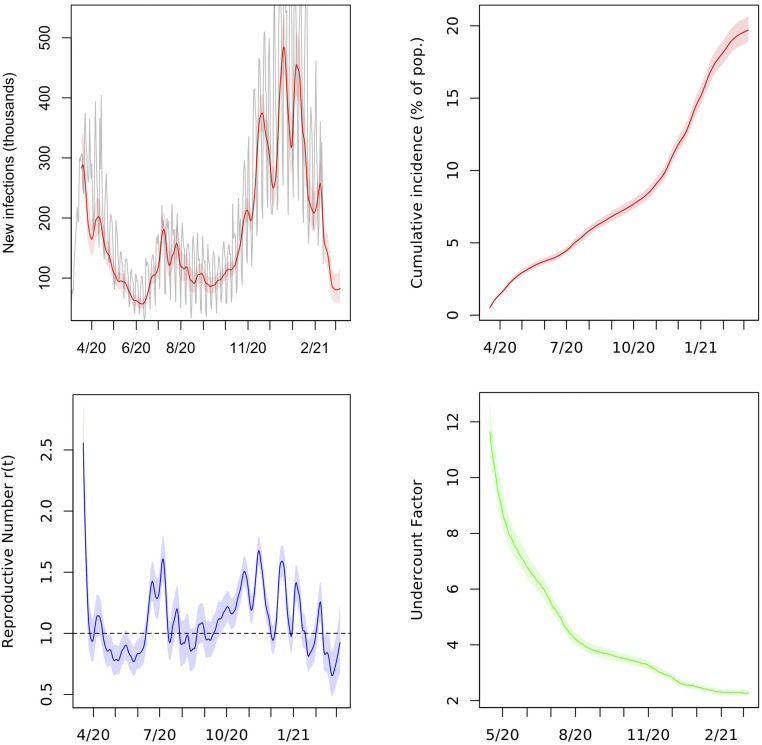
Aggregated estimates of new infections, cumulative incidence, reproductive number r(t), and cumulative undercount for the United States from March 2020 to March 2021. In *Upper Left*, deaths (in thousands) divided by 0.0068 and shifted back 23 days are plotted in gray for comparison. Pop., population.

We summed posterior samples of the SIR trajectories from all of the states to obtain estimates of viral prevalence in the United States on each day. The results are summarized in Fig. 3. For each sampled trajectory of the infection curve, we calculated an effective contact parameter $\beta_{t}$ for the entire country for each day from the SIR Eq. **1**.

As of March 7, 2021, we estimate that 19.7% of the US population, or about 65 million people, had been infected with SARS-CoV-2. This suggests that the United States was far from reaching herd immunity and that it was unlikely to do so from infections alone in the short term while state and local governments continued to implement lockdowns and other mitigations. Up to that date, we estimate that 1 out of every 2.3 infections in the United States had been confirmed via testing. This implies that approximately 60% of all infections in the country had gone unreported.

In Fig. 3, *Upper Left*, which exhibits estimates of new infections on each day in the United States, we plot reported COVID deaths per 1,000 population shifted back 23 days (which is the mean of the time-to-death distribution τ). In the plot, we divide deaths per 1,000 by 0.0068. This is the point estimate of IFR reported by Meyerowitz-Katz and Merone (13) in their meta-analysis of 24 IFR estimates from a wide range of countries published between February and June 2020. The two curves have a substantial overlap, suggesting that the IFR implied by our estimates of true infections in the United States is consistent with their findings.

### Implications for Herd Immunity.

**Fig. 4.**
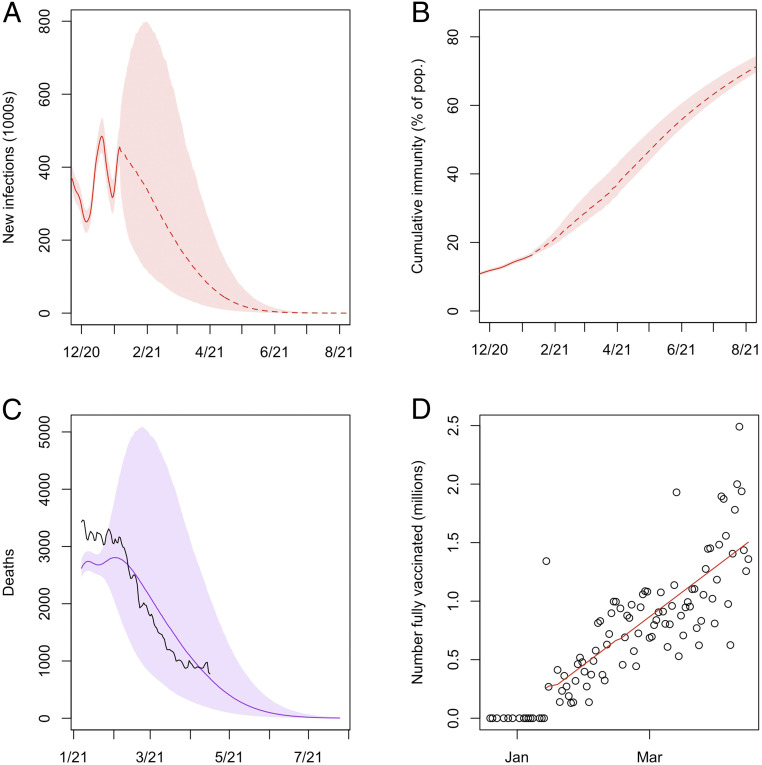
(*A*–*C*) The 95% credible intervals for the predictive distributions of new infections (*A*), cumulative immunity (viral incidence and full vaccinations) (*B*), and COVID deaths (*C*) in the United States projected out from January 2021 through July 2021. In *C*, the 7-day moving average of COVID deaths is plotted in black. We use *New York Times* data (15) for deaths after March 7, 2021, the last day reported by the COVID Tracking Project. (*D*) Scatterplot of the number of people newly fully vaccinated on each day in the United States as reported by Our World in Data (14). The line of best fit for the data from January 14, 2021 (the first day on which a positive number is reported) is plotted in red. Pop., population.

To illustrate the potential use of our method, we conducted a simulation to assess the implications of our results for herd immunity in the United States. We project the SIR model for the United States forward from January 6, 2021, and incorporate vaccine administration into the dynamics. We make the following strong assumptions:

1) Recovered individuals are immune to the virus, i.e., reinfection does not occur.
2) Immunity is conferred upon becoming fully vaccinated. Data tracking the number of people in the United States fully vaccinated on each day are available from Our World in Data (14). Beyond April 16, 2021, we assume that the number fully vaccinated on each day follows the linear trend it had exhibited so far until reaching 2 million per day (Fig. 4*D*). After that, we assume that the number fully vaccinated per day remains at 2 million.
3) After January 6, the reproductive number r(t) follows an autoregressive AR(1) model with mean estimated from the sampled posterior trajectories of r(t) through January 6.

The first point merits further discussion. Our projections that follow are particularly sensitive to this assumption. It may turn out that individuals who have been vaccinated or previously infected are still susceptible to new variants of the virus that are cropping up and will continue to spread. It is also possible that the natural immunity conferred by asymptomatic and mild infections that elicited minimal immune response, which constitute a large portion of the total, will not last long enough to prevent widespread reinfection in the next few months. In either case, if Assumption 1 is violated, then we may experience further waves of infection and delayed progress toward herd immunity.

Assumption 3 requires that the reproductive number oscillates around its mean, which is approximately 1.1 based on our estimates of r(t) through January 6, 2021. This assumption is borne out by the plots of r(t) in Figs. 1–3 and for many other states in *SI Appendix*. A possible explanation for this trend is the public and governmental response to deviations of r(t) from one. As r(t) exceeds one and cases rise, lockdowns and other nonpharmaceutical interventions may be implemented to contain the virus; as r(t) drops below one and cases dwindle, businesses, such as bars and restaurants, may be allowed to reopen, causing r(t) to increase. When fitting the model in time periods for which we have data, we assumed instead that r(t) follows a random walk, as described in *Materials and Methods*. This process works well for estimation, but the variance can increase too much if used for projection beyond the short-term. The stochastic AR model captures future uncertainty in r(t) more accurately. We also estimate the autocorrelation and variance parameters of the AR(1) model from the sampled trajectories of r(t).

We project the 40,000 samples from the posterior distribution of the US infection trajectory forward under the modified SIR model described above. New infections and cumulative immunity (the percentage of the population previously infected or fully vaccinated) on each day are plotted in Fig. 4. Based on our simulation, we find that the number of new infections per day in the country would likely fall below 5,000, about 1/100th of the winter peak, by June 2021, if our assumptions are valid. At this point, the virus’ spread through the population will have been effectively suppressed. In getting there, we project that we will incur another 18 million to 31 million new infections, beginning from January 7. These numbers are obtained as the interquartile range of the projected cumulative incidence. Note that at that point, our model suggests that cumulative immunity will be 60% or less.

To put this in perspective, there were about 360,000 confirmed COVID deaths and 52 million infections (by our reckoning) as of January 6, 2021. Assuming an IFR of 0.68%, the additional 18 million to 31 million new infections would lead to 186,000 to 270,000 more COVID deaths, with 173,000 to 245,000 occurring between January 7 and April 16, 2021. According to COVID data reported by the *New York Times* (15), there were 204,000 COVID deaths in the country between January 7 and April 16, which is consistent with our projections. Fig. 4*C* also demonstrates that the predictive distribution of deaths from our projections matches up well with the data. We find that the projections given here are not very sensitive to plausible modifications of Assumptions 2 and 3.

## Discussion

To craft and implement effective policy and mitigation strategies, policymakers need reliable assessments of the impact of previous nonpharmaceutical interventions on the transmission rate of the disease. We have developed a simple Bayesian model of the dynamics of SARS-CoV-2 transmission incorporating readily available time-series data tracking the virus, as well as statewide representative point-prevalence surveys conducted in Indiana and Ohio, which are the highest-quality random testing surveys carried out to date. We present estimates of the IFR and the time-varying viral prevalence and reproductive number r(t) in each US state on each day. Our results indicate that a large majority of COVID infections go unreported. Even so, we find that the United States was still far from reaching herd immunity to the virus in early March 2021 from infections alone. This suggests that continued mitigation and an aggressive vaccination effort are necessary to surpass the herd-immunity threshold without incurring many more deaths due to the disease. This work demonstrates the value of random-sample testing in response to this and future pandemics.

By incorporating testing and case data aggregated over any period of time, our additive model for positive tests in Eq. **2** allows us to avoid using data at the daily level, which can be very unreliable. For example, the reported cumulative number of tests administered in a state may not be updated for up to 2 wk at a time, or it may decrease from one day to the next as data are deduplicated upon further review. The latter scenario frequently occurs with reported cases as well. Working with data at the daily level generally requires using some kind of moving average, which washes out stochasticity in the data and leads to oversmoothing inconsistent with the high overdispersion of SARS-CoV-2 transmission (16).

Our inference relies on daily reported deaths due to COVID in each state, as opposed to excess deaths. Because of the possibility of death misclassification, excess-death data represent a mix of confirmed COVID deaths and deaths from other causes. Nevertheless, relying on reported deaths is a potential source of bias, as they are affected by the accuracy of cause-of-death determinations. Their numbers can fall significantly below excess-death counts and may undershoot the true number of deaths due to the disease (1). Ascertainment of COVID deaths may vary between states, with the cumulative excess-death count since the start of the pandemic exceeding reported COVID deaths by upwards of 50% in some states, according to a *New York Times* analysis of Centers for Disease Control and Prevention (CDC) mortality data (17). Consequently, our results may underestimate viral incidence in those states.

The CDC estimated a total of 83 million infections in the United States through December 2020 (18), which is substantially larger than our estimate of 50 million infections in that period. Their numbers are based on the work of Reese et al. (19), who infer COVID incidence in the United States using a multiplier model to account for underdetection in the number of confirmed cases. Beyond the limitations of our study discussed above, there are a few possible explanations for the difference in our estimates. Reese at al. (19) base their estimates on nationally reported laboratory-confirmed cases, which do not constitute a probabilistic sample of the population. To this point, the authors remark that “…some infections, such as those among healthcare workers or from outbreaks in congregate residential settings, may be more likely to be tested and nationally reported compared with the general population, and could overestimate nonhospitalized cases and infections.” Furthermore, the multiplier in their model relies on documented rates of test administration and care-seeking among symptomatic COVID patients. Reese et al. note that data on rates of test administration in this group are limited, especially at the local level. As such, Reese et al. do not account for geographic variation in testing, which is a potential source of bias.

## Materials and Methods

### SIR Model.

We first define our discrete-time SIR model for infections in each state. Let $S_{t}$ denote the number of susceptible people in the population on day t, $I_{t}$ the number of infections, and $R_{t}$ the number removed. The number removed includes those who have died of the disease and those who have recovered and are assumed immune for the rest of the period of our study. With N denoting the state population, these quantities evolve in time according to the equations

$$\left\{\begin{array}{cc} S_{t+1}-S_{t}\; & =-\frac{\beta_{t}}{N}I_{t}S_{t},\\ I_{t+1}-I_{t}\; & =\frac{\beta_{t}}{N}I_{t}S_{t}-\gamma I_{t},\\ R_{t+1}-R_{t}\; & =\gamma I_{t}. \end{array}\right.$$ . [1]

Note that $\nu_{t}=S_{t-1}-S_{t}$ is the number of new infections on day t. We allow the parameters $\beta_{t}$, interpreted as the mean number of contacts per person on day t, to vary over time. This accounts for variation in exposure due to implementation or loosening of social distancing and other policy measures over time. We model $\beta_{t}$ as a random walk with step size σ estimated from the data, $\beta_{t+1}∼\text{Normal}\;\left(\beta_{t},\sigma^{2}\right)$. We assume that $\gamma^{-1}$, the average length in days of the infectious period, is determined by the disease and is therefore constant over time.

### Likelihood on Deaths.

Let $\tau =\left\{\tau_{0},\tau_{1},\ldots ,\tau_{m}\right\}$ denote the distribution of time to death for those infected individuals who die from the disease, i.e., $\tau_{s}$ is the probability of death s days after infection, conditional on death occurring. Similar to Johndrow et al. (5), who calibrated τ by matching quantiles of a negative binomial distribution to case data from China (20, 21), we assume that τ follows a NegativeBinomial(α,1/(β+1)) distribution with parameters α=21,β=1.1, and we truncate the distribution at the 99th percentile, or m=40 days, to rule out extremely delayed deaths. We denote by $D_{t}$ the reported deaths due to COVID on day t, which we obtain from the COVID Tracking Project (2). We link the daily new infection counts $\nu ={\left(\nu_{t}\right)}_{t}$ to reported deaths via the likelihood $D_{t}\overset{\text{ind.}}{∼}\text{Poisson}\left(\text{IFR}\sum_{k=1}^{t}\nu_{k}\tau_{t-k}\right)$.

### Representative Random Prevalence Surveys.

To pin down the IFR, we add likelihood components incorporating the Indiana and Ohio prevalence survey data (3, 4). Active viral prevalence in Indiana in the period April 25 to 29, 2020, was estimated as ${\hat{\theta }}_{v}=1.74\%$. We model this quantity using a normal approximation to the binomial distribution, ${\hat{\theta }}_{v}∼\text{Normal}\left(\theta_{v},\frac{\theta_{v}\left(1-\theta_{v}\right)}{n_{v}}\right)$, where $\theta_{v}=\left(\sum_{t=T_{1}}^{T_{2}}I_{t}\right)/N\left(T_{1}-T_{2}\right)$ is the average viral prevalence between days $T_{1}$ = April 25 and $T_{2}$ = April 29. Here, $n_{v}=3,605$ is the number of viral tests administered. Similarly, the estimated seroprevalence in the testing period, ${\hat{\theta }}_{s}=1.09\%$, is modeled as ${\hat{\theta }}_{s}∼\text{Normal}\left(\theta_{s},\frac{\theta_{s}\left(1-\theta_{s}\right)}{n_{s}}\right)$, where $\theta_{s}=\sum_{t=T_{1}}^{T_{2}}R_{t}/N\left(T_{1}-T_{2}\right)$ and $n_{s}=3518$. These results come from the first phase of the Indiana prevalence survey described in Menachemi et al. (3). The sampled population consisted of all noninstitutionalized Indiana residents aged ≥12 years listed on state tax returns, including filers and dependents. Stratified random sampling was conducted by using Indiana’s 10 public health preparedness districts as sampling strata, and 15,495 participants were contacted by the state health department. Of those contacted, 3,658, or 23.6%, agreed to participate in the study. While low, this response rate is not far from the survey industry average of 30% (22, 23). Menachemi et al. (3) note that respondents might have been subject to response bias, which could have resulted in underestimates or overestimates. To adjust for differences in nonresponse between groups, data were weighted for age, race, and Hispanic ethnicity. Participants were tested for active infection via RT-PCR and past infection via antibody test between April 25 and April 29, 2020. The RT-PCR tests used had high, but imperfect, sensitivity, and the antibody tests had high, but imperfect, specificity. The former could have caused false-negative results and the latter false-positive results. In a follow-up paper published in PNAS, Yiannoutsos et al. (24) conducted a Bayesian analysis of the Indiana survey data to address uncertainty in the results related to imperfect testing and difference in prevalence among subgroups characterized by ethnicity, race, and age. Due to very low response rates—less than 8% in the second and third phases—we do not include data from the subsequent phases of the Indiana study in our analysis.

The likelihood for the prevalence survey data from Ohio is analogous. The survey design was a stratified two-stage cluster sample, with strata defined by eight administrative regions in the state of Ohio. Within each region, 30 census tracts were randomly selected with probability proportional to total population. Within each tract, households were randomly sampled, and one adult within each household was randomly selected to participate in the study. Of those contacted, 727, or 18.5%, agreed to participate. Between July 9 and July 28, 2020, participants were tested for active and past infection via RT-PCR and antibody test. Due to the low response rate and imperfect diagnostic tests used in the study, the same caveats described above for the Indiana survey apply. Kline et al. (4) conducted a Bayesian analysis of the seroprevalence survey data to address the uncertainty in the results associated with nonresponse and imperfect testing. As reported in ref. 4, the estimated seroprevalence in the state was ${\hat{\theta }}_{s}=1.3\%$ in the period July 9 to 28, with a sample size of $n_{s}=667$. Results from the PCR tests in the same study were reported in a press conference on October 1 available on YouTube (ref. (25), minute 22). The viral prevalence in that period is estimated as ${\hat{\theta }}_{v}=0.9\%$ with sample size $n_{v}=727$. To the best of our knowledge, these numbers have not yet been published.

### Modeling Preferential Testing.

**Fig. 5.**
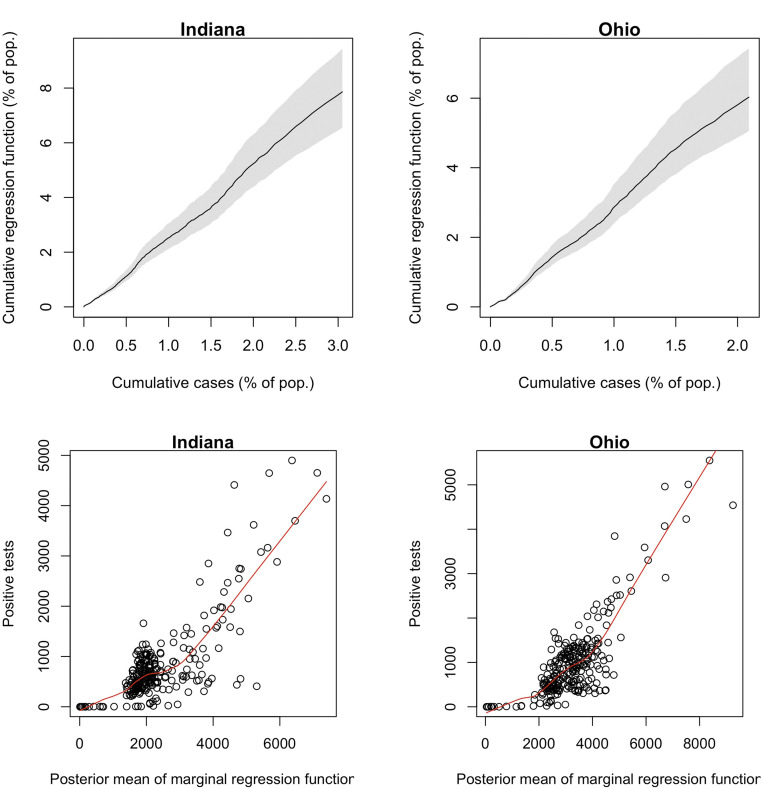
(*Upper*) Posterior median and 95% confidence bands for the cumulative regression function in Eq. **2** plotted against cumulative cases in Indiana and Ohio. (*Lower*) Positive tests on each day plotted against the posterior mean of the marginal regression function in Eq. **3**. Locally estimated scatterplot smoothing curves are plotted in red. Pop., population.

As shown in Figs. 1 and 2, the undercount curve $\left(I_{t}+R_{t}\right)/\left(\sum_{k\leq t}C_{k}\right)$ has a common shape in Indiana and Ohio. Here, $I_{t}$ and $R_{t}$ are the SIR parameters on day t, and $C_{t}$ is the total number of confirmed and probable cases, defined as unique people with a positive PCR or other approved nucleic acid amplification test in the state on day t, as reported by the COVID Tracking Project (2). We found that the reciprocal of the undercount is approximately linear when plotted against the square root of the cumulative number of tests administered in the state on each day and that the slopes of these lines for the two states are similar; Fig. 5. This led to the following model for the test data:

$$\overset{t}{\underset{k=1}{\sum }}C_{k}∼\text{Normal}\left(\phi_{t}\left(I_{t}+R_{t}\right),\eta_{t}^{2}\right).$$ [2]

Here, the parameters $\phi_{t}$ and $\eta_{t}$ are proportional to the square root of the fraction of the population tested up to day t,

$$\phi_{t}=\phi \sqrt{\frac{\sum_{k=1}^{t}T_{k}}{N}},\;\eta_{t}^{2}=\eta^{2}\frac{\sum_{k=1}^{t}T_{k}}{N},$$

so that $\phi_{t}$ is the overall fraction of infections that appear in the cumulative number of positive tests. We assume that this fraction grows as the state’s test capacity ramps up and that the variance in this relationship, $\eta_{t}^{2}$, grows linearly with the total number of tests administered. Here, $T_{t}$ is the number of total test results in the state on day t, as reported by the COVID Tracking Project (2). Due to variation in test-reporting methods across states, this number may include antigen tests as well as viral (PCR) tests. Moreover, different states report total tests using different units, whether in terms of test encounters, test specimens, or unique people tested. As such, $T_{t}$ is best understood as an estimate of the state’s test capacity. This is the extent to which it is used in our preferential testing model. For example, we do not model test positivity rates $C_{t}/T_{t}$ on each day.

To arrive at the distribution in Eq. **2**, we can model the cases on each day independently as

$$C_{t}\overset{ind.}{∼}\text{Normal}\left(\phi_{t}\left(I_{t}+R_{t}\right)-\phi_{t-1}\left(I_{t-1}+R_{t-1}\right),\eta^{2}\frac{T_{t}}{N}\right).$$ [3]

Noting that $\nu_{t}=\left(I_{t}+R_{t}\right)-\left(I_{t-1}+R_{t-1}\right),$ we can write the mean of $C_{t}$ as

$$\phi_{t}\cdot \nu_{t}+\left(\phi_{t}-\phi_{t-1}\right)\left(I_{t-1}+R_{t-1}\right).$$

Hence, in expectation, $C_{t}$ can be decomposed as a fraction of the new infections on day t, $\nu_{t}$, and a smaller fraction of the cumulative incidence on day t−1, $I_{t-1}+R_{t-1}$.

In fitting the model, we do not use the likelihood on each day [**3**] due to inconsistent reporting of cases and tests, as well as weekly oscillations in these numbers due to reduced reporting on weekends. Rather, in each state, we combine cases and tests into nonoverlapping consecutive L-day periods, where L is at least seven to account for weekend effects, and model the counts in these periods independently.

We first fit the model in Indiana and Ohio without the likelihood on cases described above. That is, initially we used only deaths data and the random-sample surveys in each state. With the resulting posterior samples of cumulative incidence $I_{t}+R_{t}$ on each day, we arrived at the likelihood on cases. Fig. 5 demonstrates the relationships defined in Eqs. **2** and **3** . We refer to the normal means in [**2**] and [**3**] (divided by the parameter ϕ) as the cumulative and marginal regression functions, respectively. Fig. 5, *Lower* reveals a comparable slope ϕ for Indiana and Ohio after a brief initial period when testing and cases were very low. The widening CIs in Fig. 5, *Upper* exhibit the growth of the variance in [**2**] as a function of cumulative testing.

A number of other models for case and test data have been proposed. Campbell et al. (26) introduced a binomial likelihood on cases, $C_{t}∼\text{Binomial}\left(T_{t},1-{\left(1-I_{t}/N\right)}^{\alpha }\right)$, where $I_{t}/N$ is the infection rate on day t, and α>0 is a parameter representing the degree of preferential testing. Assuming the infection rate is small, a binomial expansion of the test positivity rate yields the approximation $1-{\left(1-I_{t}/N\right)}^{\alpha }\approx \alpha I_{t}/N$. An application of Bayes’ rule to the latter model shows that α=P(tested|infected)/P(tested). This model has some limitations in the context of our study. Firstly, the degree of preferential testing α is likely to decrease as testing increases, and it is not obvious how one might parametrize $\alpha =\alpha_{t}$ to account for this. Secondly, the model is not additive, as the test positivity relies on the active infection rate. As a result, it is not well suited to handling state-level testing data, which can be unreliable on the daily level.

Youyang Gu (27) and Peter Ellis (28) proposed similar models to correct case counts using test positivity rates. They take the form $\nu_{t}=C_{t}\left[m\cdot {\left(C_{t}/T_{t}\right)}^{k}+b\right]$, where m>0,k∈[0,1],b≥0 are parameters. Benatia et al. (29) also estimate population prevalence on day t by the number of positive tests on day t scaled by a multiplicative factor depending on the number of tests administered on day t as a fraction of the state population. These models are susceptible to the same issues as that of Campbell et al. (26). They rely on daily test positivity rates, which are reported inconsistently across states (30). And as Youyang Gu (27) notes, the parameters estimated at one point in time do not carry over to other time periods. Furthermore, by assuming that new infections are a function only of cases and tests on that day, these models ignore the lag between infections and their confirmation via testing. They also presume that there are no new infections on days in which no positive tests are reported. Our likelihood on cases in Eq. **3** allows for new infections to be reflected in case counts at a later date.

Note that our model does not take into account imperfect testing. Modeling imperfect testing is complicated by the inconsistent test-reporting methods across states described above, which obscure the true number of PCR tests administered in a state on each day. Given that estimated active infection rates are generally low (<5%) at any given time, imperfect test specificity (i.e., the proportion of true negative results) is a greater potential source of bias in case counts $C_{t}$ than sensitivity. False positives resulting from imperfect specificity would increase $C_{t}$. We note, however, that the molecular RT-PCR assays widely deployed to test for the presence of viral RNA are shown to have near perfect specificity (31–34).

### Prior Specification.

Lastly, we specify prior distributions for the model parameters $\left\{\text{IFR},\beta_{1},\sigma ,\gamma^{-1},\left(S_{1},I_{1}\right),\phi ,\eta \right\}$. We used a weakly informative Uniform(0,0.03) prior distribution for the IFR in each state. For Indiana, we used a truncated normal prior for the mean infectious period, $\gamma^{-1}∼{\text{Normal}}_{\left[5.5,11.5\right]}\left(8.5,1.5^{2}\right)$. This is motivated by clinical data, which show that most infected individuals remain infectious no longer than 10 days after symptom onset (35–42) and that patients can be highly infectious several days before symptom onset (43).

We assumed that the removal rate γ is determined by the disease and so does not vary between states. Therefore, after fitting the model to the data for Indiana, we used the posterior distribution of γ for Indiana as the prior distribution of γ for Ohio. We then used the posterior distribution from Ohio as the prior distribution for the remaining states, each of which we modeled independently. The prior distributions of the remaining parameters are diffuse independent uniform priors. Their exact forms are provided in *SI Appendix*. To estimate ϕ, we used the same process as described for γ.

### Implementation.

We built the model in R and fit it with the RStan software package, which implements the No-U-Turn-Sampler for Bayesian inference (44–46). For each state, we ran four chains in parallel for 20,000 steps each, with the first 10,000 as burn-in, to obtain 40,000 samples from the posterior distribution of the model parameters. Code to fit the model is available at the GitHub repository (47).

### Data Cleaning.

In certain states, the COVID Tracking Project data report a negative number of cases, tests, or deaths on some days, often due to record deduplication or changes in data reporting by the state government. If a negative number of cases or tests is reported, we address this by setting that datum to zero and distributing the negative number over all previous days proportional to the number of cases or tests reported on those days. If a negative number of deaths is reported, we set that datum to zero and subtract the negative number from the deaths reported on the previous day. If this results in a negative number of deaths on the previous day, we continue this procedure until all counts are nonnegative.

The COVID Tracking Project also notes days when state governments report a backlog of cases or deaths, which usually results in a large spike in the data on that day. We address this by setting that datum to the average of the number of cases or deaths reported on the day before and the day after and distributing the excess number of cases or deaths over all previous days proportional to the number of cases or deaths reported on those days.

## Supplementary Material

Supplementary File

## Data Availability

Code to fit the model is available at the GitHub repository (https://github.com/njirons/covidest) (47). An online dashboard displaying our results is available (https://rsc.stat.washington.edu/covid-dashboard) (11). Previously published data were used for this work (Covid Tracking Project; https://covidtracking.com/) (48).
