# Supplementary material for: Estimating SARS-CoV-2 infections from deaths, confirmed cases, tests, and random surveys
Source: Proc Natl Acad Sci U S A. 2021 Jul 26;118(31):e2103272118. doi: 10.1073/pnas.2103272118 (PMC8346866; doi:10.1073/pnas.2103272118)
Supplement: Supplementary File [file pnas.2103272118.sapp.pdf]

Estimating SARS-CoV-2 Infections from Deaths, Confirmed Cases, Tests, and Random Surveys: Supporting Information

Nicholas J. Irons and Adrian E. Raftery

Prior specification

Here we list the prior distributions used for the parameters of our model in each state. Apart from the removal rate γ and the preferential testing parameter ϕ, which are assumed to be the same across states and therefore obtained by composing models as described below, we use the following weakly or uninformative independent uniform priors for the remaining parameters:

$$
\begin{aligned}
\text{IFR} &\sim \text{Uniform}(0, 0.03), \\
\beta_1 &\sim \text{Uniform}(0, 1), \\
\sigma &\sim \text{Uniform}(0, 1), \\
\eta &\sim \text{Uniform}(0, 3), \\
(S_1, I_1, R_1) &\sim 0.01N \cdot \text{Dirichlet}(1, 1, 1) + (0.99N, 0, 0).
\end{aligned}
$$

In the last line N denotes the state population. This distribution specifies that no more than 1% of the state has been infected as of the first day for which COVID Tracking Project data are available for the state (i.e., the susceptible population is $S_1 \geq 0.99N$), which is generally between late February and mid-March 2020. Note that the Dirichlet$(1, 1, 1)$ distribution is the uniform distribution on the 2-dimensional simplex. While the contact parameter on the first day β_1 is given an uninformative uniform prior, on subsequent days we assume that β_t follows a random walk model, $\beta_{t+1} \sim \text{Normal}(\beta_t, \sigma^2)$.

In Indiana, the prior density for the duration of the infectious period is a truncated normal distribution informed by the epidemiology literature, $\gamma^{-1} \sim \text{Normal}_{[5.5,11.5]}(8.5, 1.5^2)$, and the prior for the preferential testing parameter is uninformative, $\phi \sim \text{Uniform}(0, 2)$. After fitting the model for Indiana, we use the posterior distribution of γ^{-1} and ϕ as the prior for Ohio. We then use the resulting posterior from Ohio as a prior for the remaining states.

SARS-CoV-2 infection estimates for all US states

Table S1 lists estimates of the COVID infection fatality rate (IFR), cumulative viral incidence, and the infection undercount factor (total infections divided by total cases) for all US states as of March 7, 2021.

Figures 1 through 51 display daily estimates of new infections, cumulative incidence, the SIR reproductive number $r(t) = \beta_t/\gamma$, and the undercount factor for all states from March 2020 to March 2021.

State	IFR	Cumulative Incidence	Undercount
Alabama	0.90% (0.70-1.15)	24.0% (18.8-30.6)	2.4 (1.8-3.0)
Alaska	0.35% (0.28-0.43)	11.8% (10.1-14.2)	1.5 (1.3-1.8)
Arizona	0.93% (0.75-1.14)	24.5% (20.1-30.3)	2.2 (1.8-2.7)
Arkansas	0.78% (0.64-0.95)	23.2% (19.2-28.4)	2.2 (1.8-2.7)
California	0.88% (0.70-1.09)	16.9% (13.6-20.9)	1.9 (1.6-2.4)
Colorado	0.60% (0.49-0.71)	17.2% (14.6-20.9)	2.3 (2.0-2.8)
Connecticut	1.37% (1.10-1.70)	15.9% (12.9-19.9)	2.0 (1.6-2.5)
Delaware	0.92% (0.74-1.13)	17.0% (14.0-21.0)	1.9 (1.6-2.3)
Florida	0.78% (0.64-0.94)	19.5% (16.3-23.8)	2.2 (1.9-2.8)
Georgia	0.67% (0.54-0.82)	25.9% (21.4-32.0)	2.7 (2.2-3.4)
Hawaii	0.70% (0.54-0.91)	4.6% (3.6-5.8)	2.3 (1.8-2.9)
Idaho	0.28% (0.23-0.33)	37.8% (32.1-45.9)	4.0 (3.4-4.8)
Illinois	0.96% (0.78-1.16)	19.3% (16.1-23.7)	2.0 (1.7-2.5)
Indiana	0.84% (0.70-1.00)	22.9% (19.2-27.6)	2.3 (1.9-2.8)
Iowa	0.76% (0.61-0.97)	23.6% (18.7-29.5)	2.6 (2.1-3.3)
Kansas	0.59% (0.47-0.75)	28.6% (22.6-36.1)	2.8 (2.2-3.5)
Kentucky	0.53% (0.43-0.63)	21.4% (18.0-25.8)	2.3 (2.0-2.8)
Louisiana	1.01% (0.82-1.22)	21.1% (17.5-26.1)	2.3 (1.9-2.8)
Maine	0.73% (0.60-0.87)	7.3% (6.2-8.8)	2.1 (1.8-2.6)
Maryland	0.94% (0.77-1.15)	13.9% (11.5-17.1)	2.2 (1.8-2.7)
Massachusetts	1.71% (1.36-2.13)	14.4% (11.6-18.0)	1.7 (1.4-2.1)
Michigan	1.00% (0.77-1.29)	17.1% (13.3-22.1)	2.6 (2.0-3.4)
Minnesota	0.64% (0.52-0.76)	18.4% (15.4-22.4)	2.1 (1.8-2.6)
Mississippi	0.74% (0.61-0.89)	31.6% (26.6-38.6)	3.2 (2.7-3.9)
Missouri	0.69% (0.55-0.87)	19.6% (15.8-24.7)	2.5 (2.0-3.2)
Montana	0.62% (0.50-0.75)	21.0% (17.5-25.5)	2.3 (1.9-2.8)
Nebraska	0.53% (0.43-0.64)	21.1% (17.7-25.7)	2.0 (1.7-2.5)
Nevada	0.73% (0.59-0.87)	22.5% (18.8-27.5)	2.4 (2.0-2.9)
New Hampshire	0.66% (0.54-0.79)	13.4% (11.3-16.2)	2.4 (2.0-2.9)
New Jersey	1.22% (0.97-1.53)	22.2% (17.7-28.0)	2.4 (1.9-3.1)
New Mexico	1.01% (0.83-1.21)	18.6% (15.6-22.7)	2.1 (1.8-2.5)
New York	1.12% (0.87-1.42)	18.6% (14.7-23.9)	2.1 (1.7-2.8)
North Carolina	0.58% (0.48-0.68)	19.4% (16.5-23.4)	2.4 (2.0-2.8)
North Dakota	0.86% (0.71-1.03)	22.4% (19.0-27.1)	1.7 (1.4-2.0)
Ohio	0.83% (0.68-1.03)	19.5% (15.9-23.7)	2.3 (1.9-2.9)
Oklahoma	0.47% (0.38-0.56)	25.1% (21.1-30.6)	2.3 (1.9-2.8)
Oregon	0.69% (0.55-0.85)	8.2% (6.7-10.0)	2.2 (1.8-2.8)
Pennsylvania	1.00% (0.82-1.19)	19.4% (16.4-23.7)	2.6 (2.2-3.2)
Rhode Island	1.41% (1.14-1.72)	17.4% (14.3-21.4)	1.4 (1.2-1.8)
South Carolina	0.76% (0.63-0.91)	23.0% (19.4-28.0)	2.3 (1.9-2.8)
South Dakota	0.68% (0.56-0.82)	31.2% (26.1-37.9)	2.5 (2.1-3.0)
Tennessee	0.79% (0.64-0.97)	21.8% (17.7-26.9)	1.9 (1.6-2.4)
Texas	0.70% (0.57-0.85)	22.7% (18.8-27.8)	2.5 (2.1-3.0)
Utah	0.22% (0.18-0.26)	28.0% (23.8-33.7)	2.5 (2.1-3.0)
Vermont	0.72% (0.57-0.92)	4.6% (3.8-5.6)	1.8 (1.5-2.2)
Virginia	1.53% (1.09-2.36)	8.3% (5.5-11.7)	1.2 (0.8-1.7)
Washington	0.51% (0.41-0.64)	12.9% (10.3-16.1)	2.9 (2.3-3.6)
West Virginia	0.86% (0.71-1.01)	15.4% (13.2-18.5)	2.0 (1.8-2.5)
Wisconsin	0.57% (0.46-0.69)	21.5% (17.9-26.6)	2.0 (1.7-2.5)
Wyoming	0.58% (0.46-0.71)	21.0% (17.3-25.9)	2.2 (1.8-2.7)
District of Columbia	1.19% (0.94-1.49)	12.2% (9.8-15.3)	2.1 (1.7-2.7)

Table S1: Posterior median and 95% intervals for IFR, cumulative incidence as of March 7, 2021, and undercount factor as of March 7, 2021.

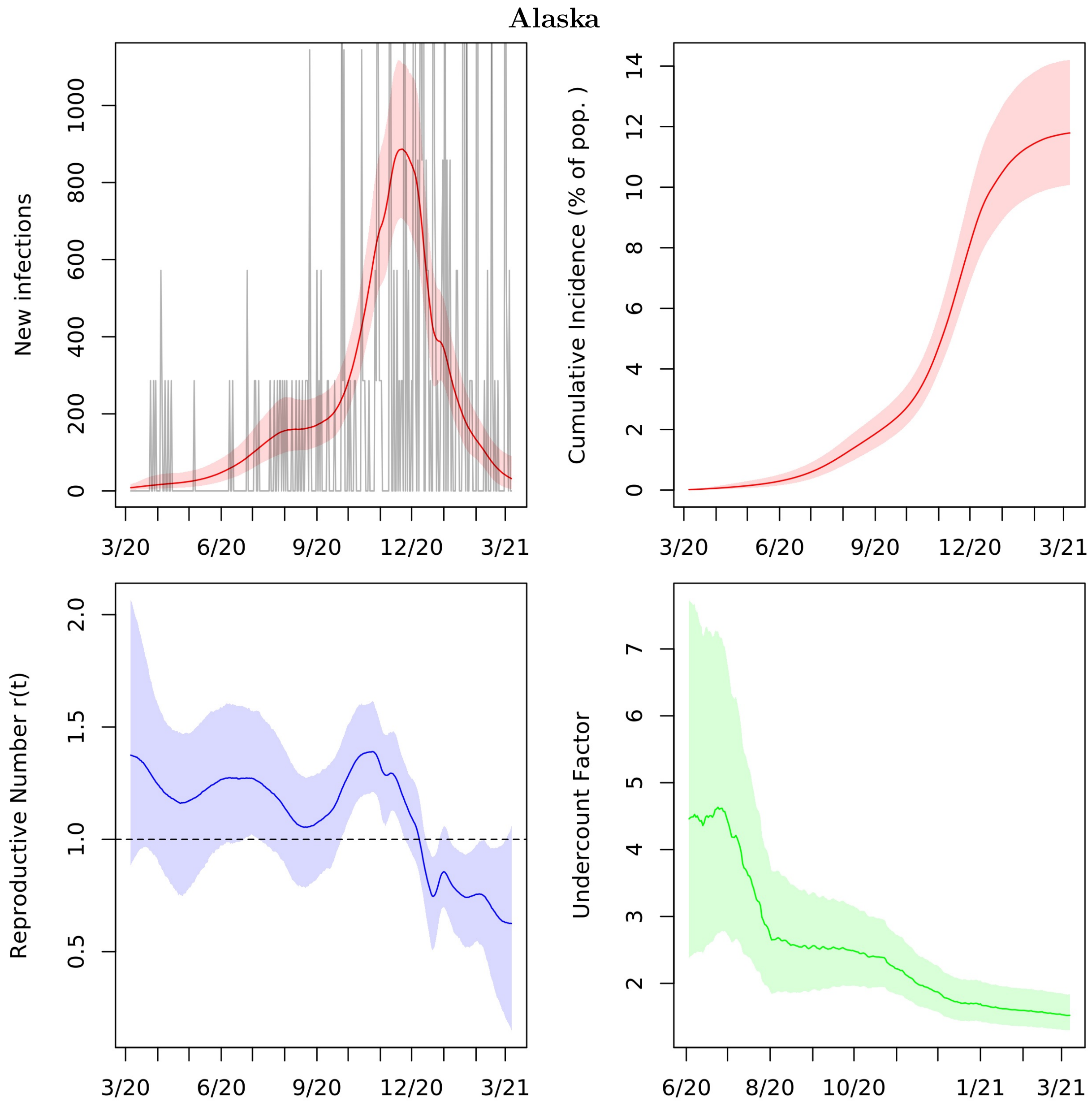

Figure 1: Posterior median and middle 95% intervals for daily new infections, cumulative incidence, $r(t)$, and cumulative undercount from March 2020 to March 2021. In the top left panel, deaths divided by the posterior median IFR are plotted in grey for comparison.

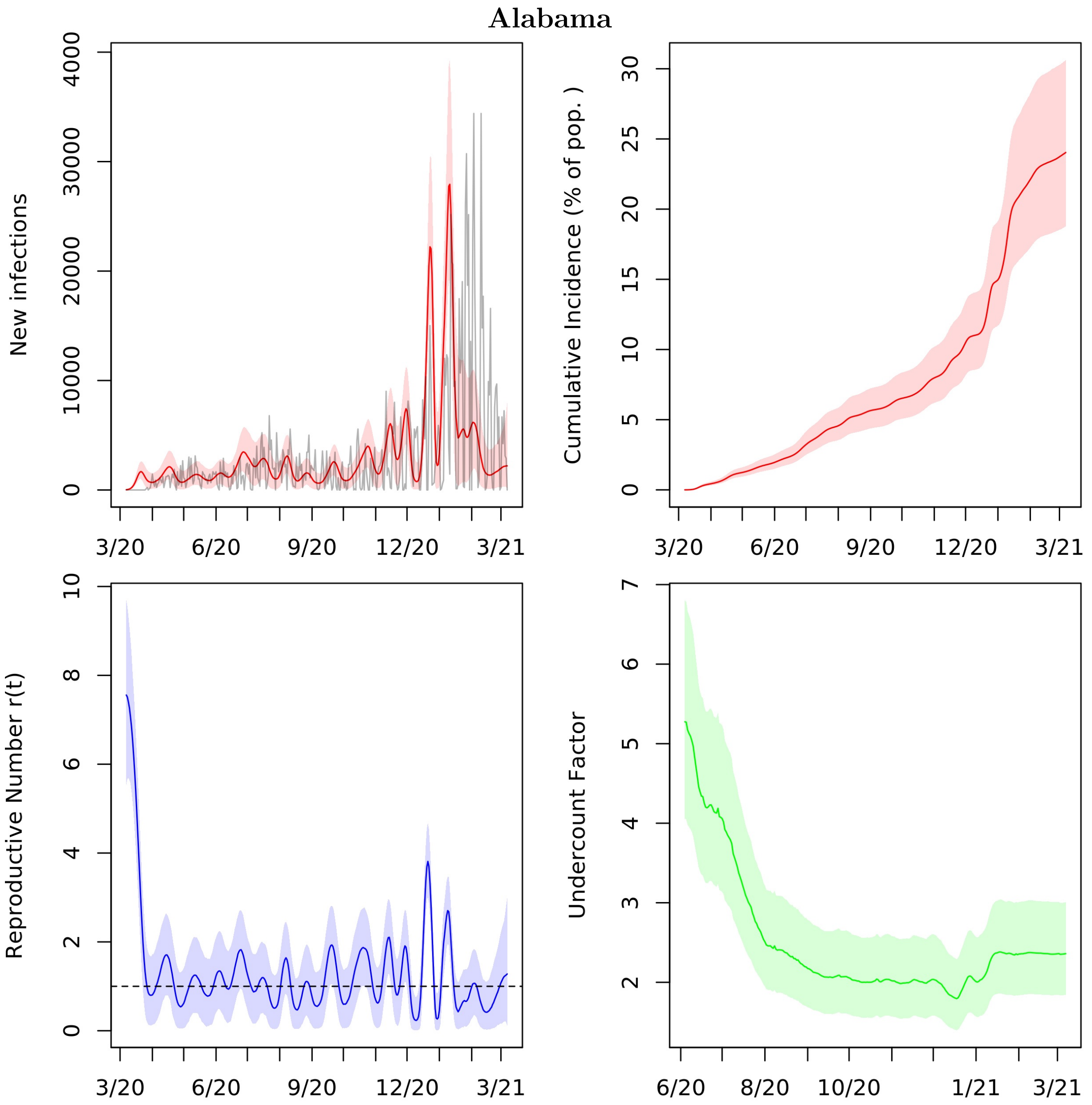

Figure 2: Posterior median and middle 95% intervals for daily new infections, cumulative incidence, $r(t)$, and cumulative undercount from March 2020 to March 2021. In the top left panel, deaths divided by the posterior median IFR are plotted in grey for comparison.

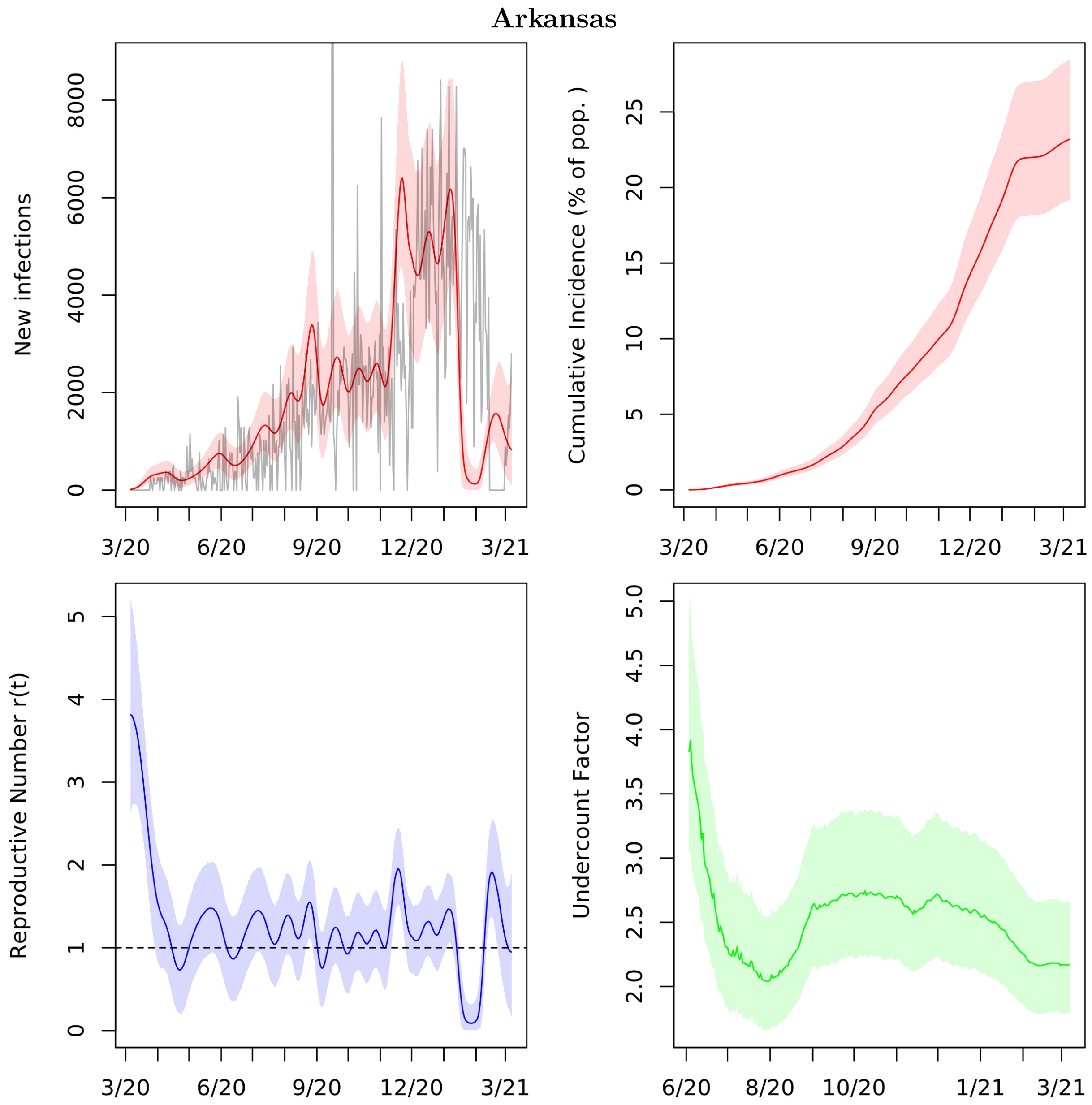

Figure 3: Posterior median and middle 95% intervals for daily new infections, cumulative incidence, $r(t)$, and cumulative undercount from March 2020 to March 2021. In the top left panel, deaths divided by the posterior median IFR are plotted in grey for comparison.

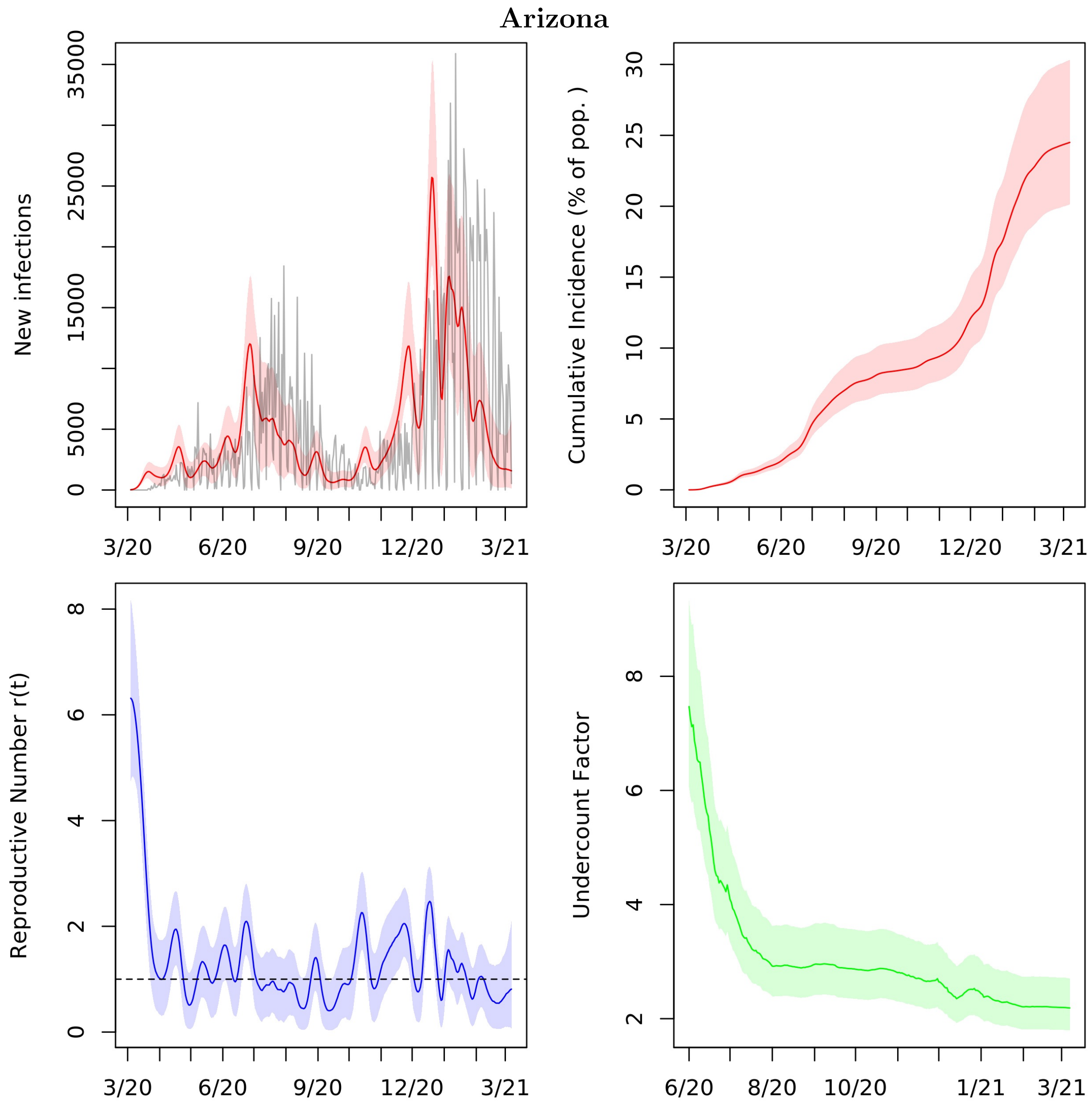

Figure 4: Posterior median and middle 95% intervals for daily new infections, cumulative incidence, $r(t)$, and cumulative undercount from March 2020 to March 2021. In the top left panel, deaths divided by the posterior median IFR are plotted in grey for comparison.

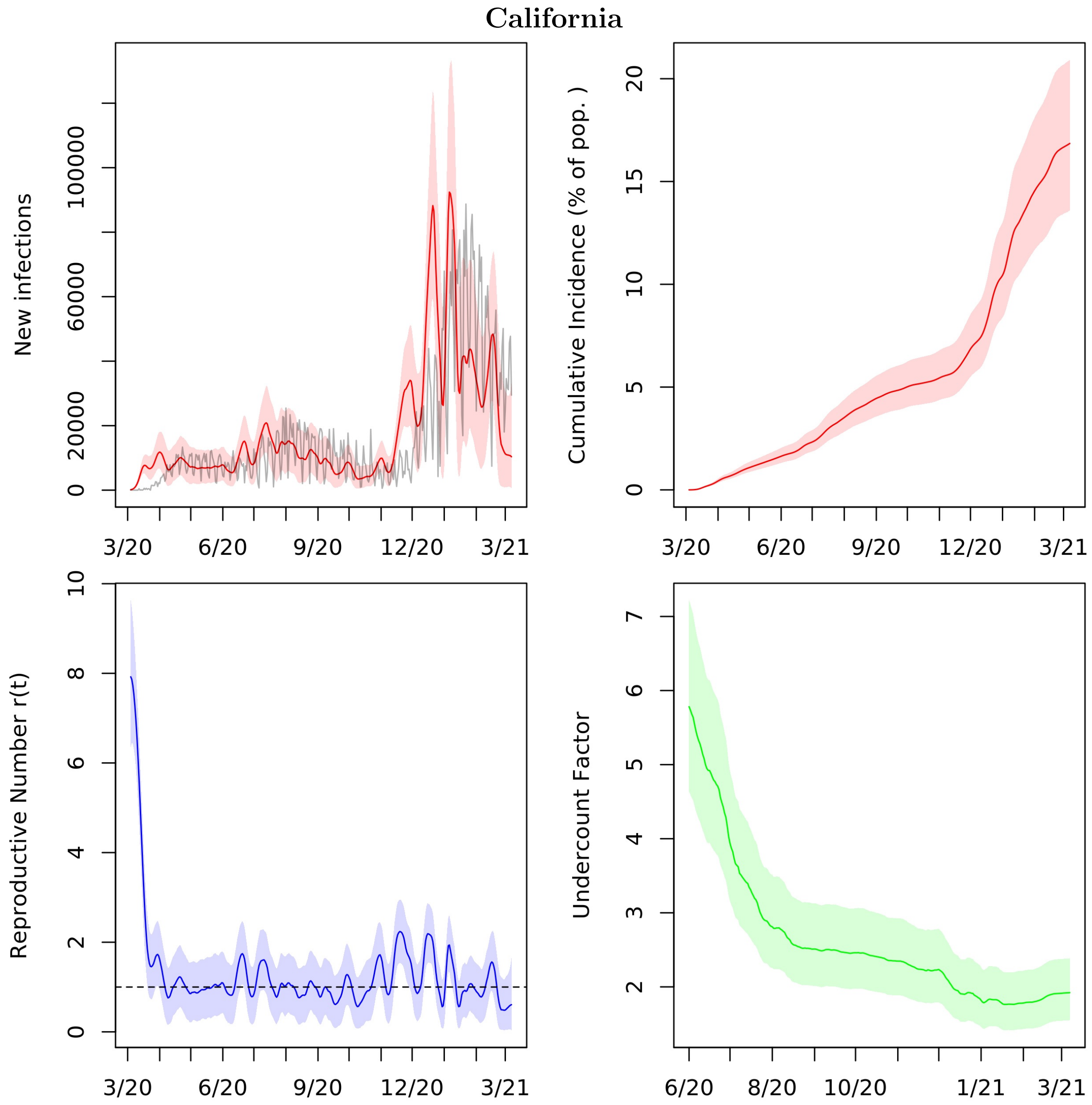

Figure 5: Posterior median and middle 95% intervals for daily new infections, cumulative incidence, $r(t)$, and cumulative undercount from March 2020 to March 2021. In the top left panel, deaths divided by the posterior median IFR are plotted in grey for comparison.

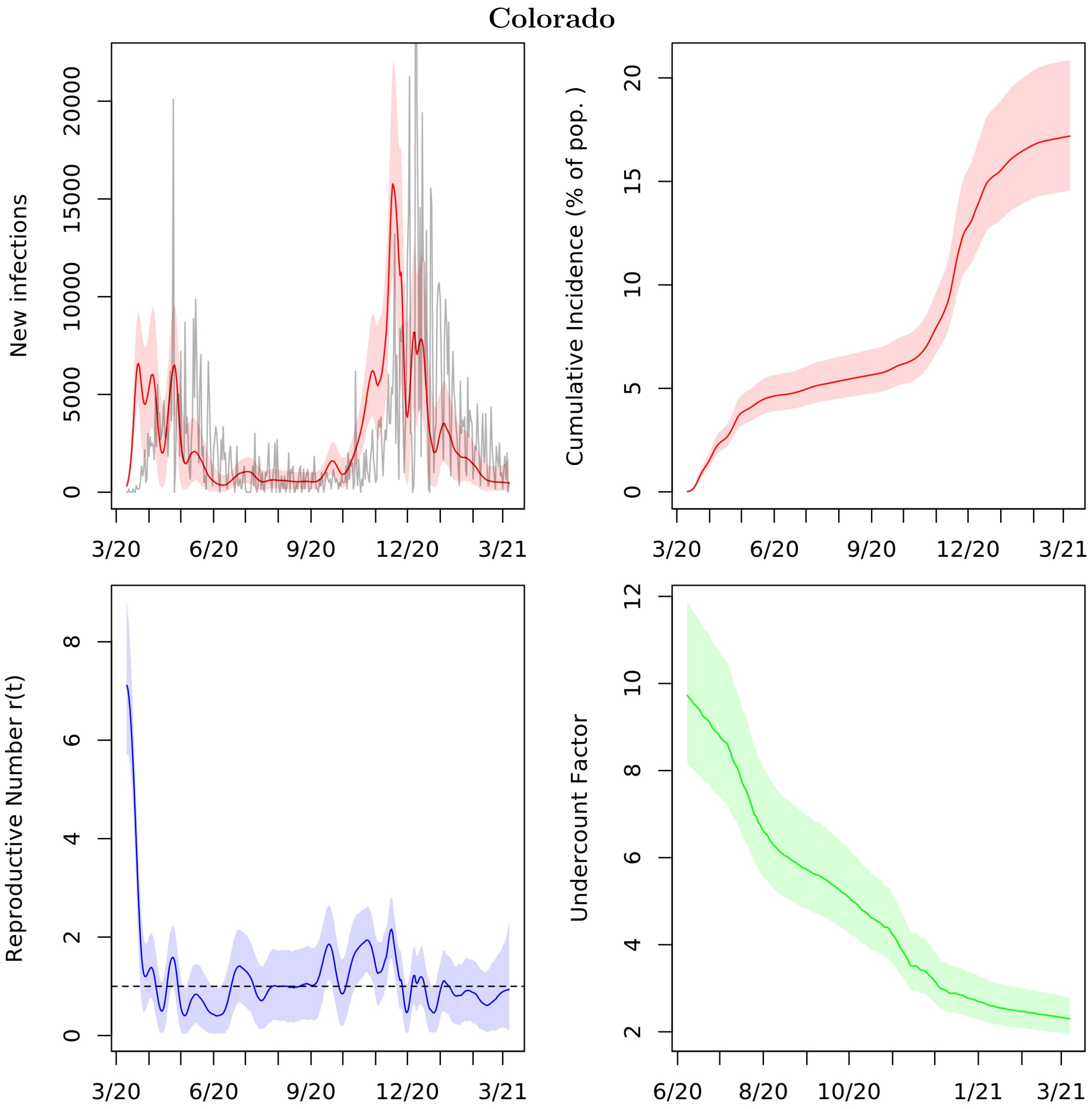

Figure 6: Posterior median and middle 95% intervals for daily new infections, cumulative incidence, $r(t)$, and cumulative undercount from March 2020 to March 2021. In the top left panel, deaths divided by the posterior median IFR are plotted in grey for comparison.

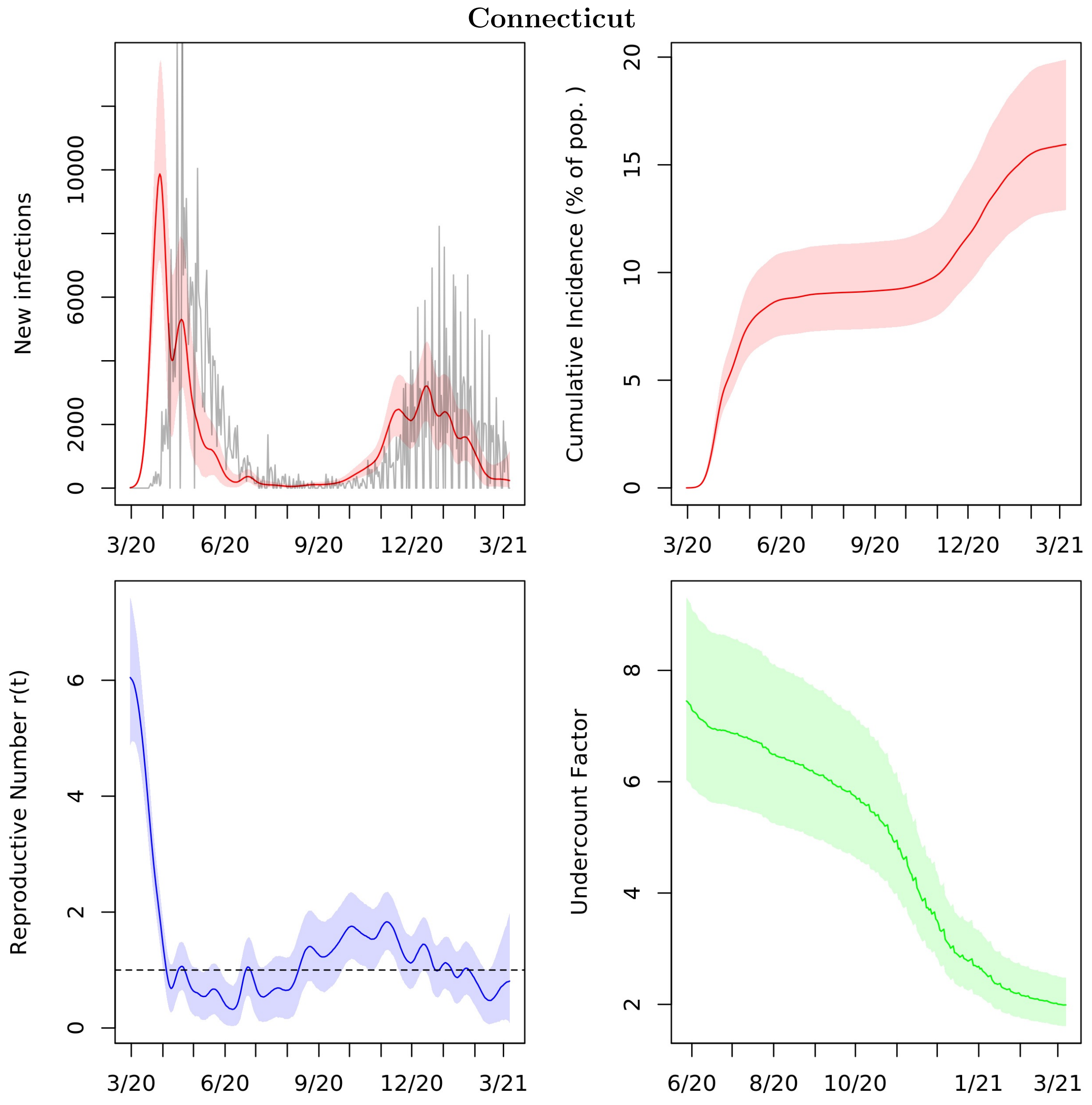

Figure 7: Posterior median and middle 95% intervals for daily new infections, cumulative incidence, $r(t)$, and cumulative undercount from March 2020 to March 2021. In the top left panel, deaths divided by the posterior median IFR are plotted in grey for comparison.

District of Columbia

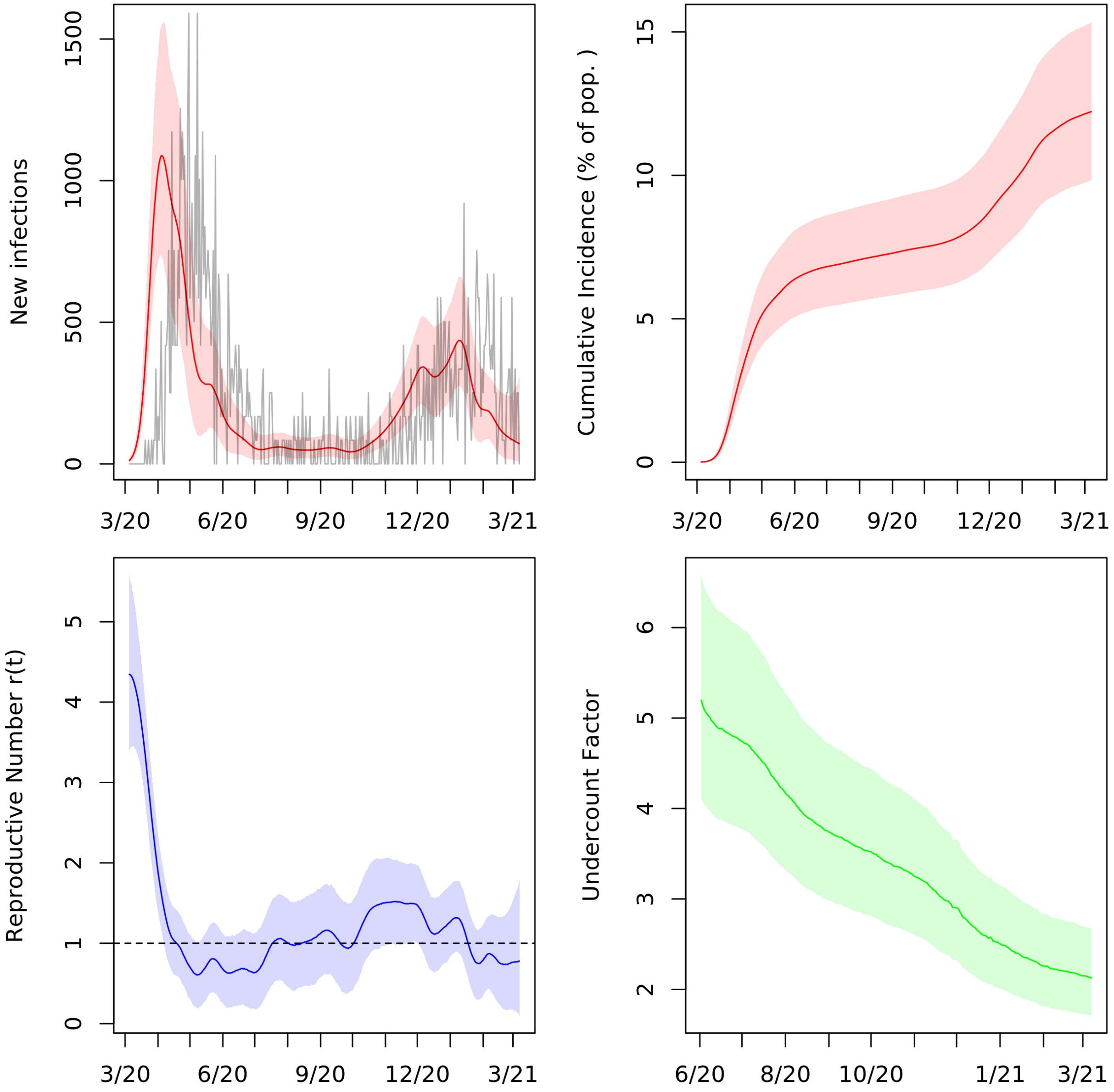

Figure 8: Posterior median and middle 95% intervals for daily new infections, cumulative incidence, $r(t)$, and cumulative undercount from March 2020 to March 2021. In the top left panel, deaths divided by the posterior median IFR are plotted in grey for comparison.

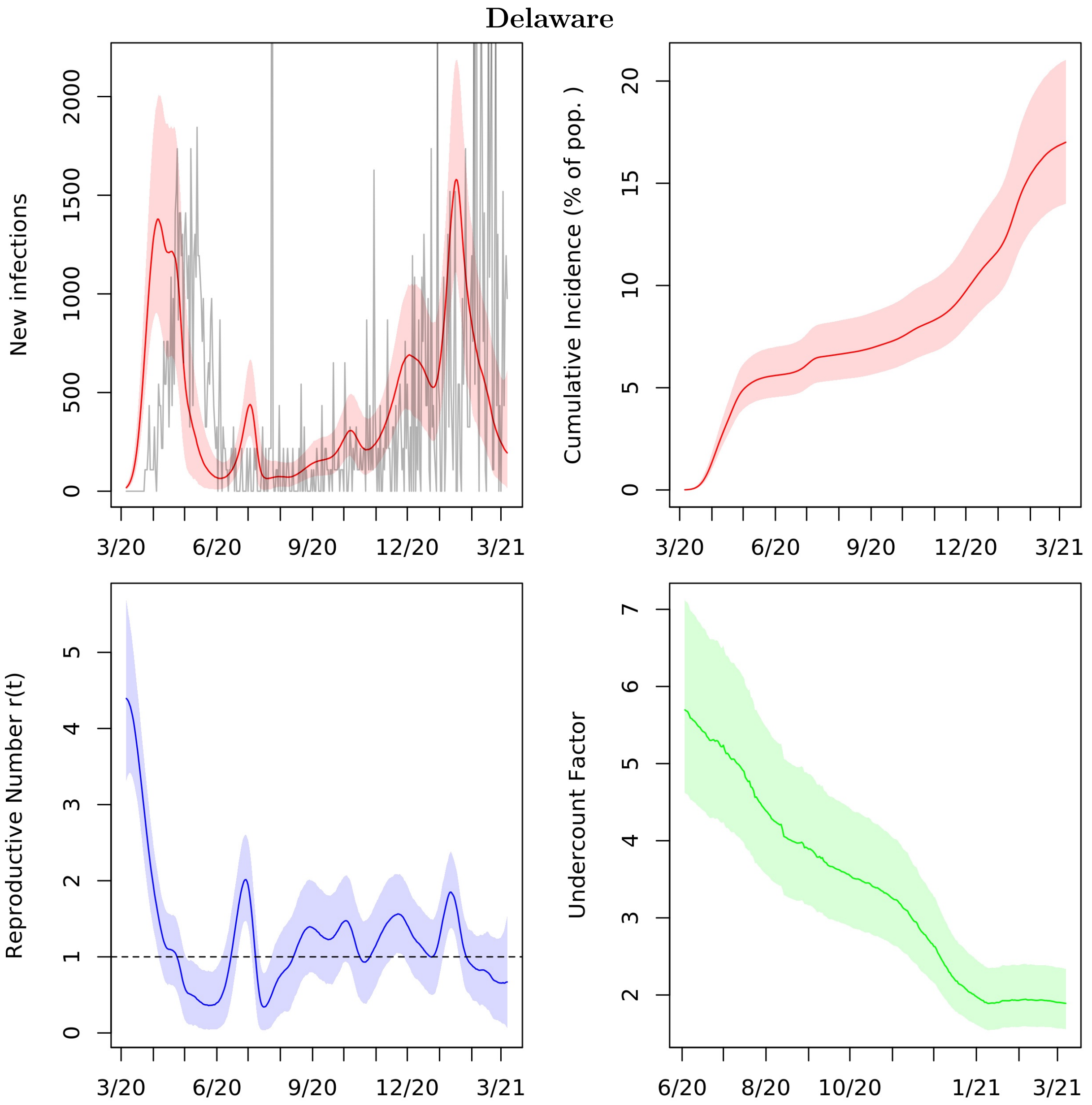

Figure 9: Posterior median and middle 95% intervals for daily new infections, cumulative incidence, $r(t)$, and cumulative undercount from March 2020 to March 2021. In the top left panel, deaths divided by the posterior median IFR are plotted in grey for comparison.

Florida

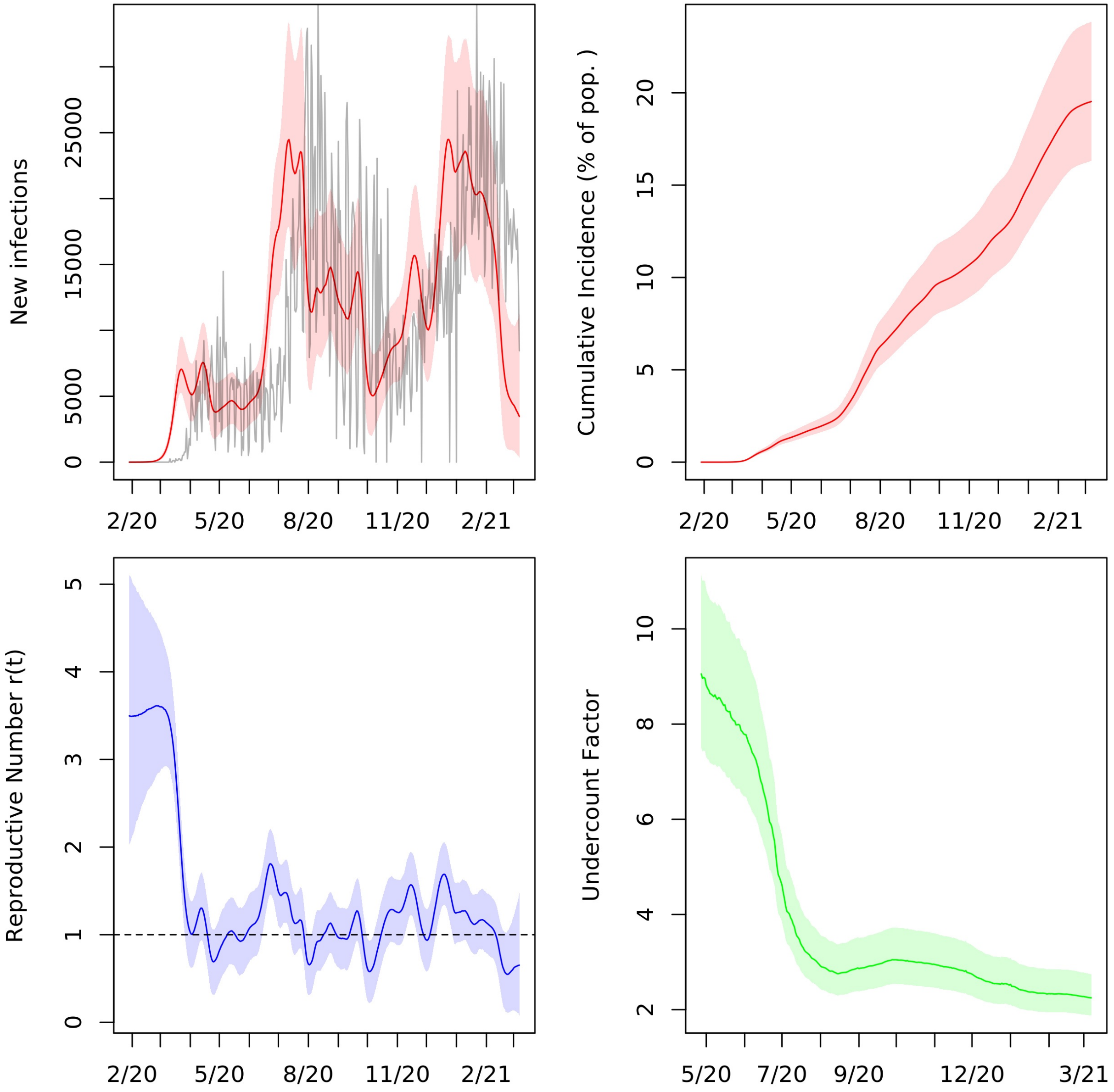

Figure 10: Posterior median and middle 95% intervals for daily new infections, cumulative incidence, $r(t)$, and cumulative undercount from March 2020 to March 2021. In the top left panel, deaths divided by the posterior median IFR are plotted in grey for comparison.

Georgia

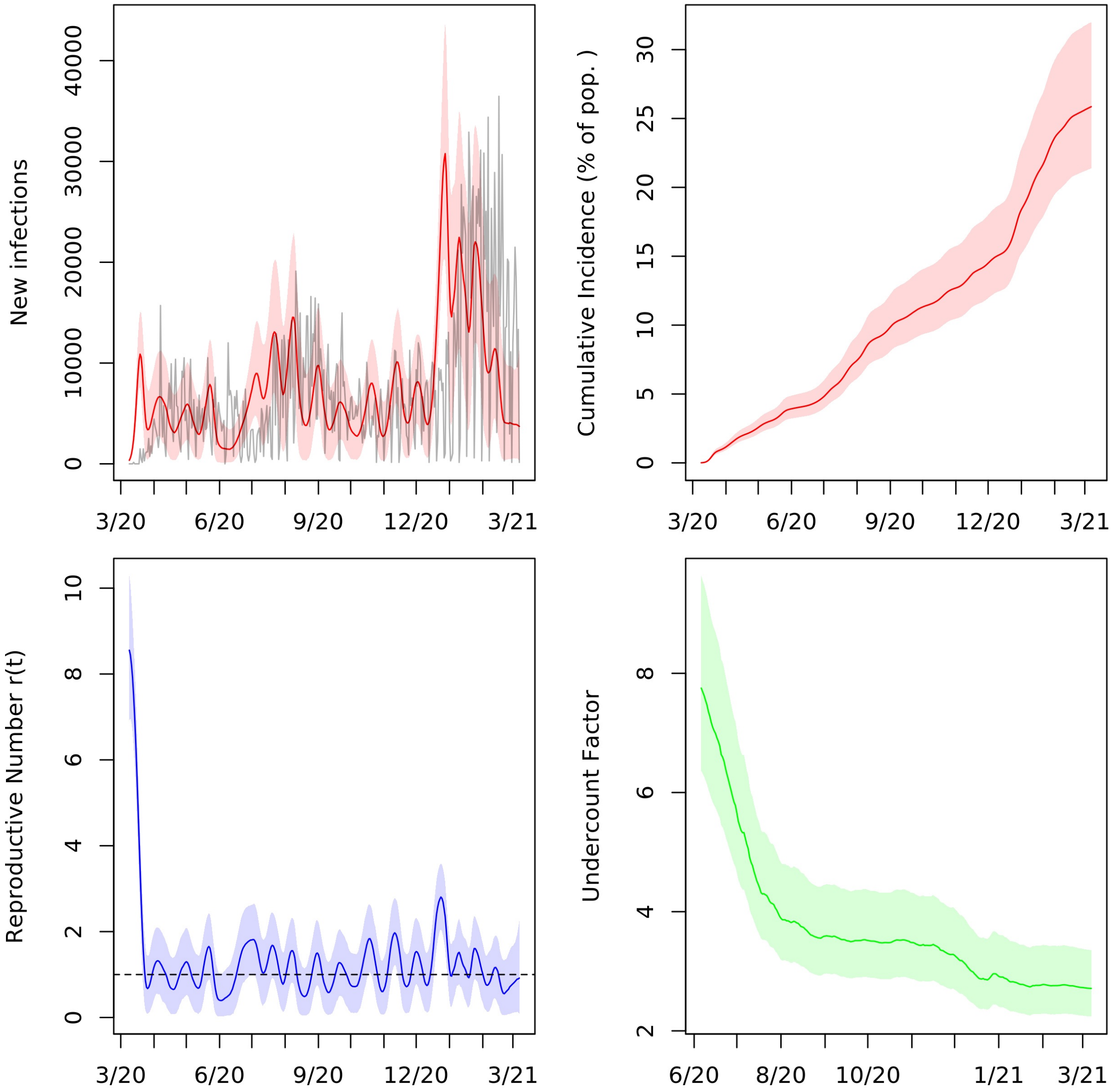

Figure 11: Posterior median and middle 95% intervals for daily new infections, cumulative incidence, $r(t)$, and cumulative undercount from March 2020 to March 2021. In the top left panel, deaths divided by the posterior median IFR are plotted in grey for comparison.

Hawaii

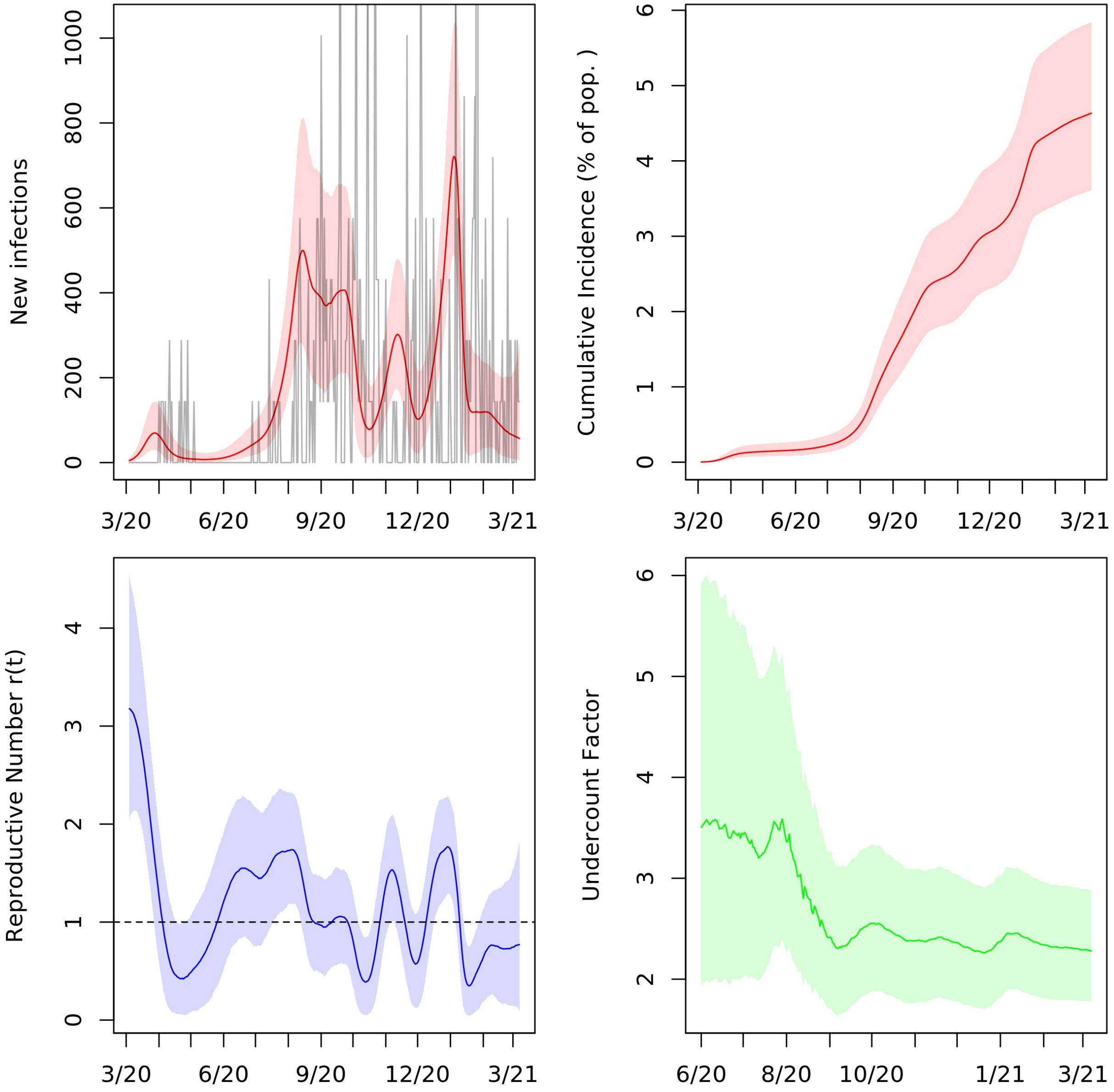

Figure 12: Posterior median and middle 95% intervals for daily new infections, cumulative incidence, $r(t)$, and cumulative undercount from March 2020 to March 2021. In the top left panel, deaths divided by the posterior median IFR are plotted in grey for comparison.

Iowa

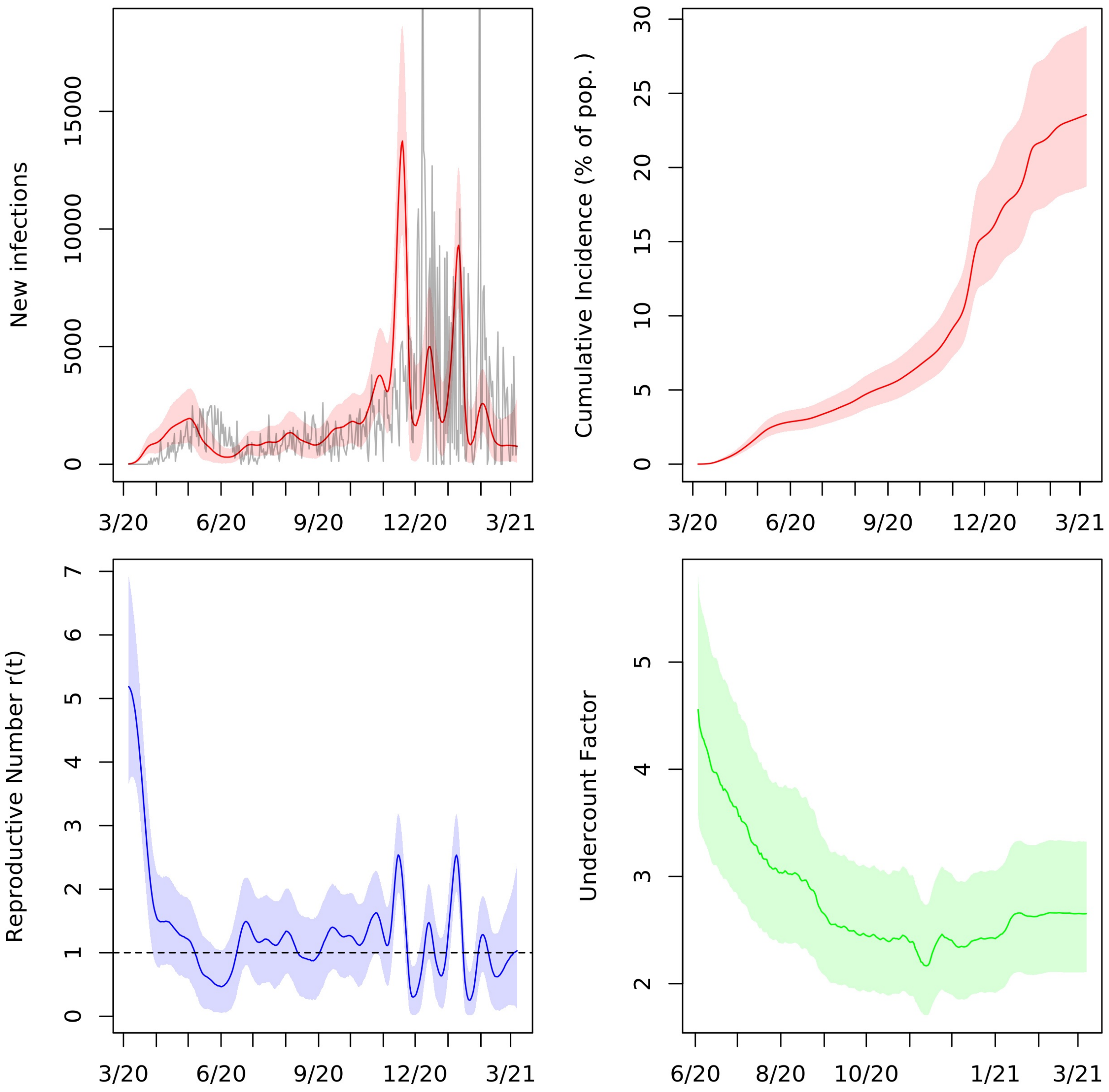

Figure 13: Posterior median and middle 95% intervals for daily new infections, cumulative incidence, $r(t)$, and cumulative undercount from March 2020 to March 2021. In the top left panel, deaths divided by the posterior median IFR are plotted in grey for comparison.

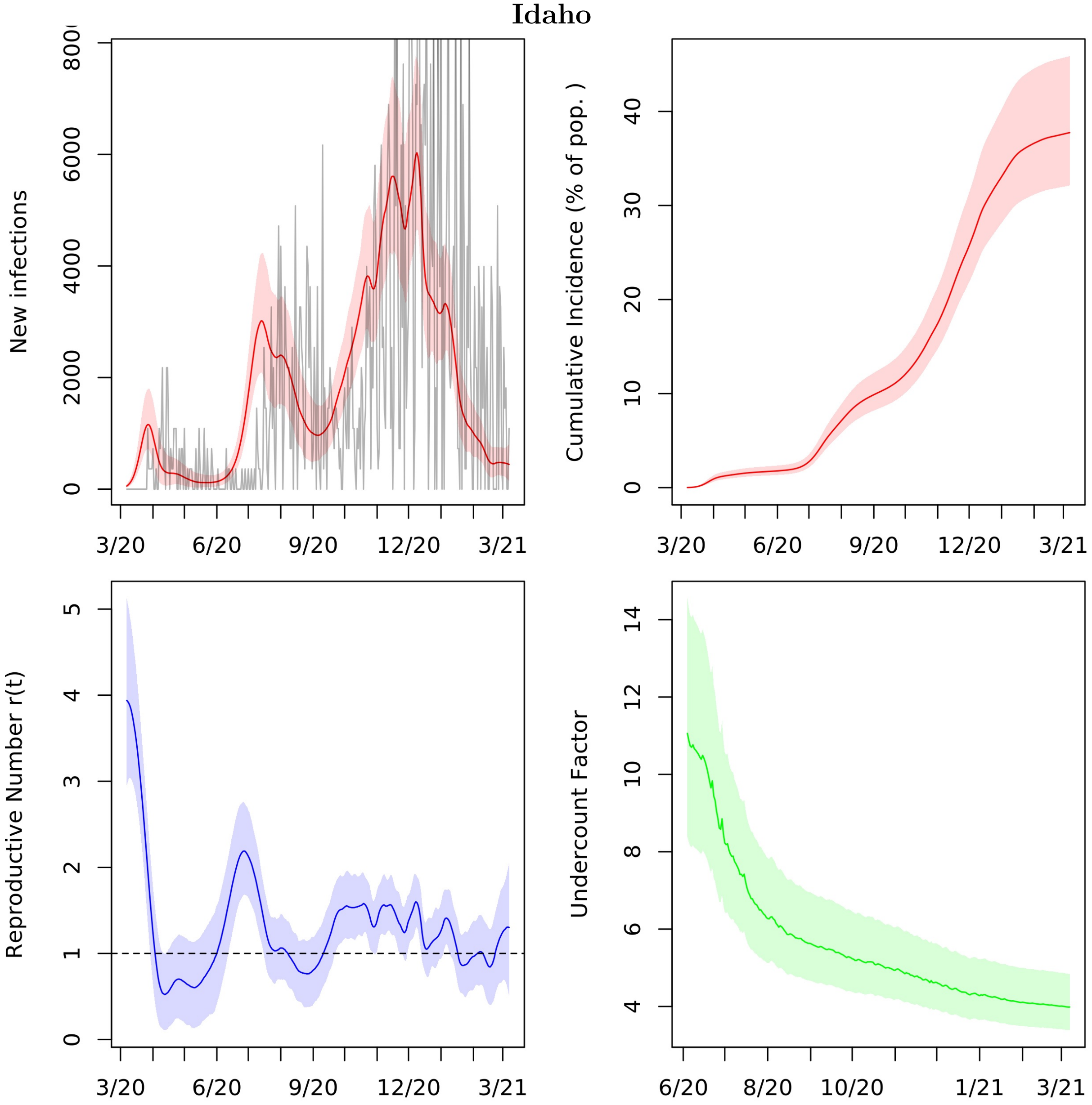

Figure 14: Posterior median and middle 95% intervals for daily new infections, cumulative incidence, $r(t)$, and cumulative undercount from March 2020 to March 2021. In the top left panel, deaths divided by the posterior median IFR are plotted in grey for comparison.

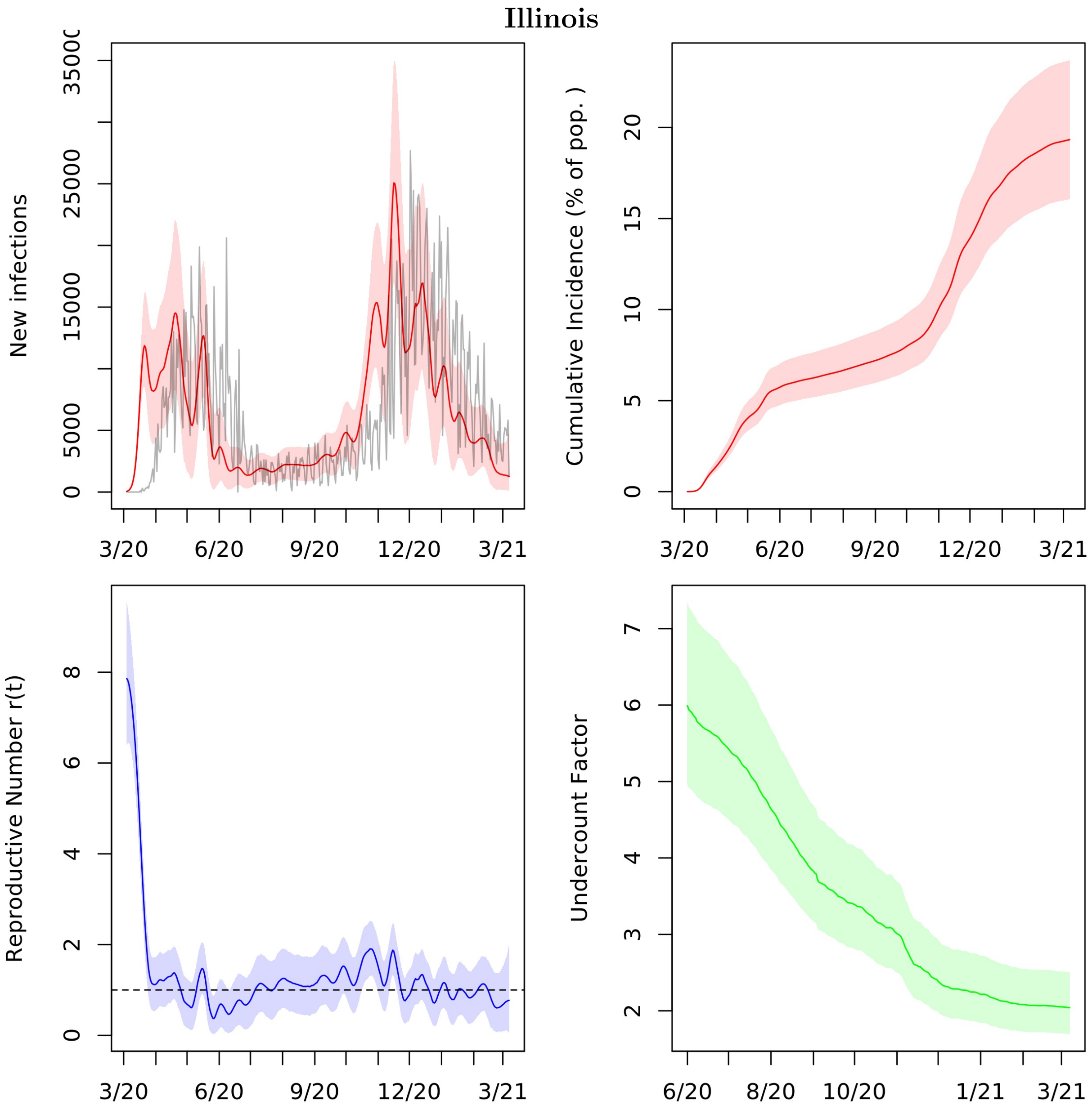

Figure 15: Posterior median and middle 95% intervals for daily new infections, cumulative incidence, $r(t)$, and cumulative undercount from March 2020 to March 2021. In the top left panel, deaths divided by the posterior median IFR are plotted in grey for comparison.

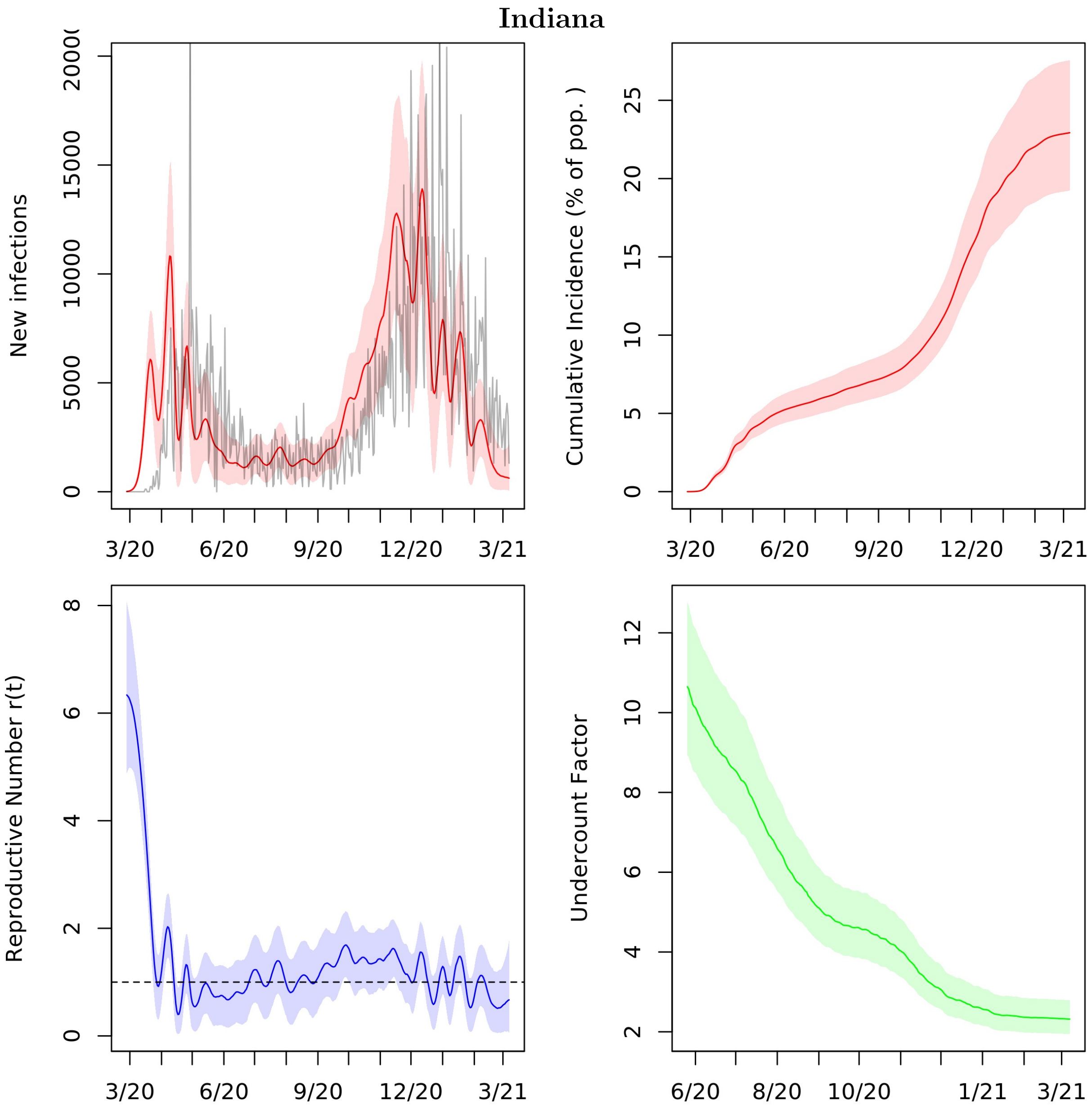

Figure 16: Posterior median and middle 95% intervals for daily new infections, cumulative incidence, $r(t)$, and cumulative undercount from March 2020 to March 2021. In the top left panel, deaths divided by the posterior median IFR are plotted in grey for comparison.

Kansas

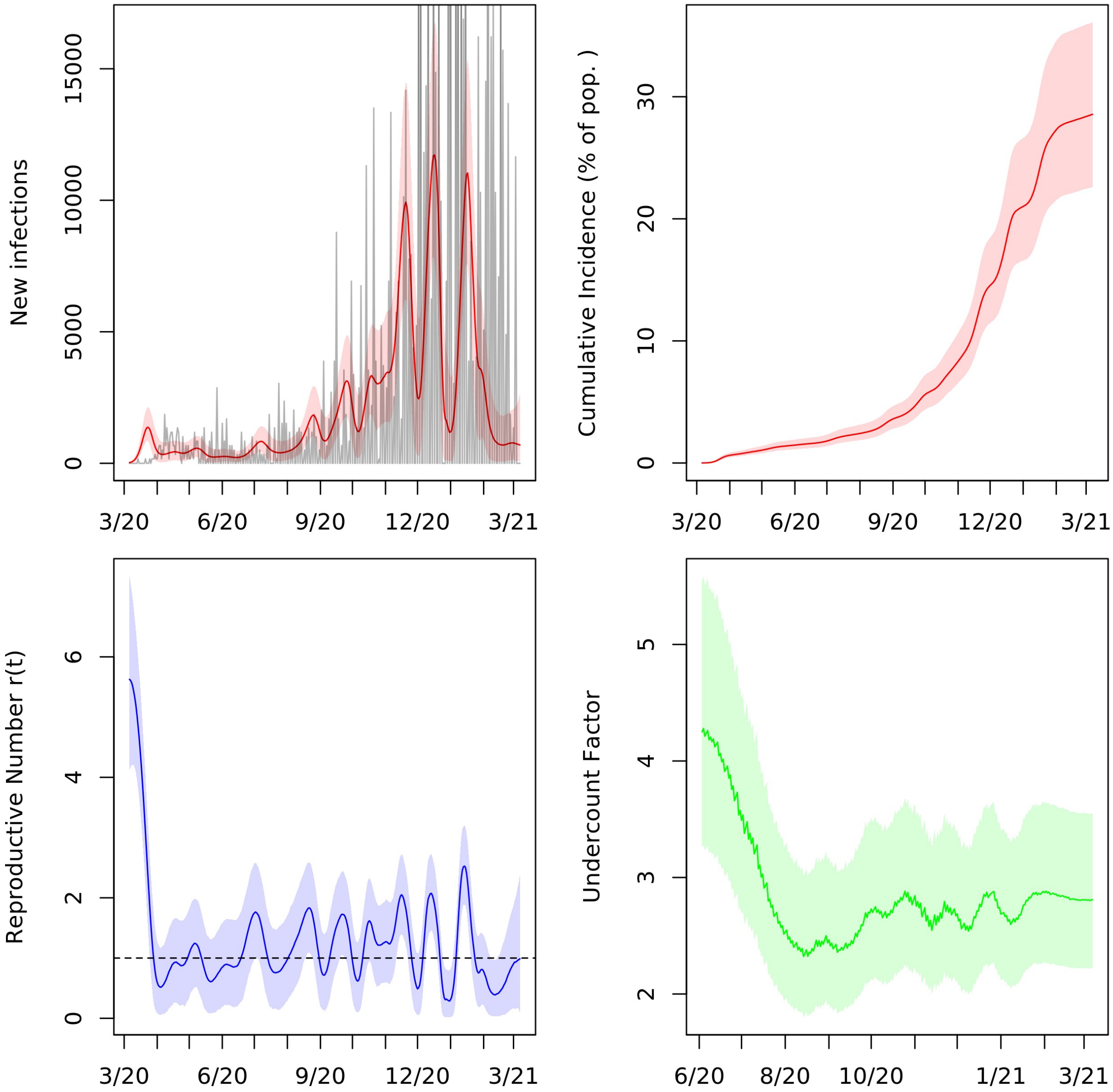

Figure 17: Posterior median and middle 95% intervals for daily new infections, cumulative incidence, $r(t)$, and cumulative undercount from March 2020 to March 2021. In the top left panel, deaths divided by the posterior median IFR are plotted in grey for comparison.

Kentucky

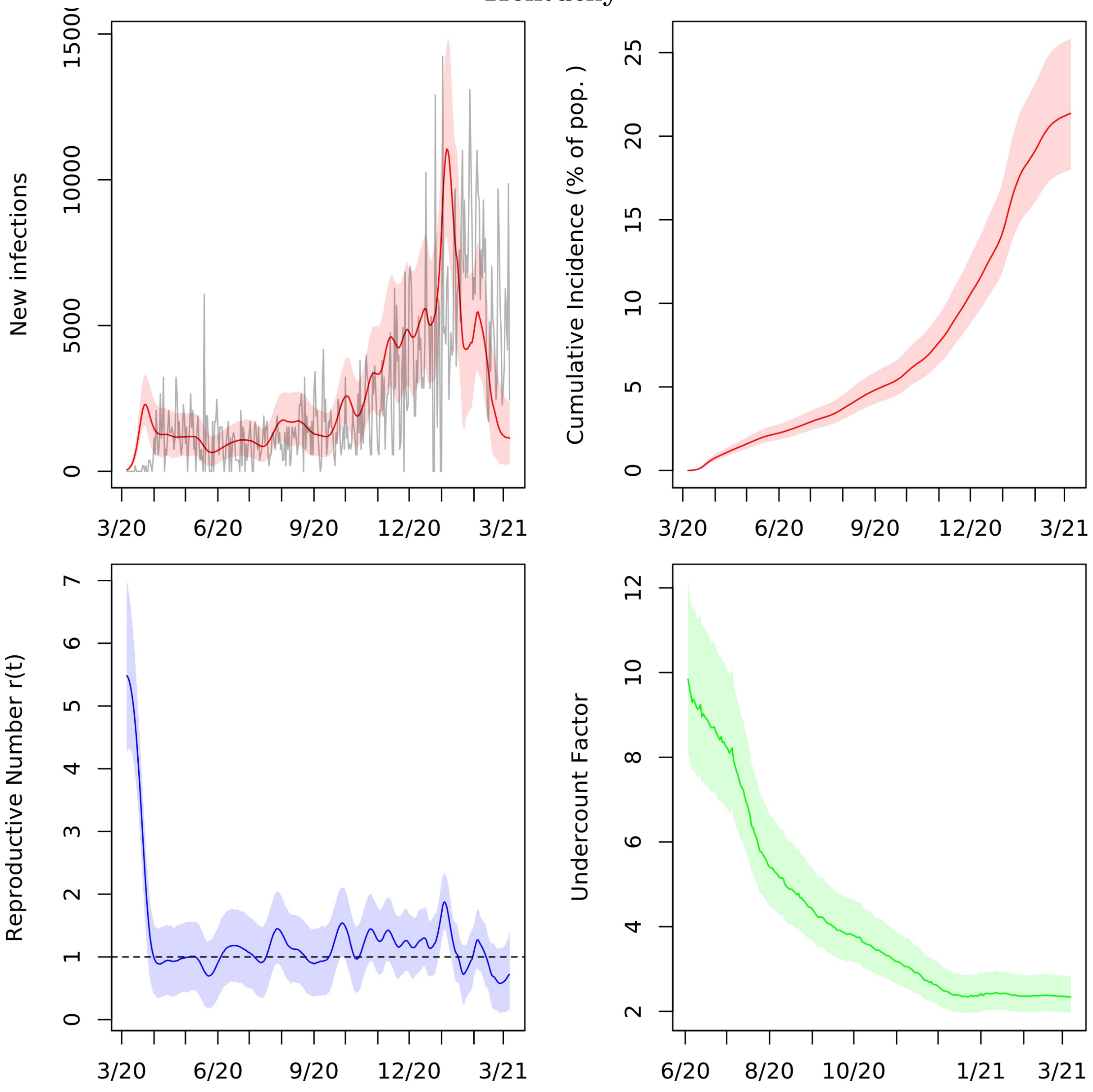

Figure 18: Posterior median and middle 95% intervals for daily new infections, cumulative incidence, $r(t)$, and cumulative undercount from March 2020 to March 2021. In the top left panel, deaths divided by the posterior median IFR are plotted in grey for comparison.

Louisiana

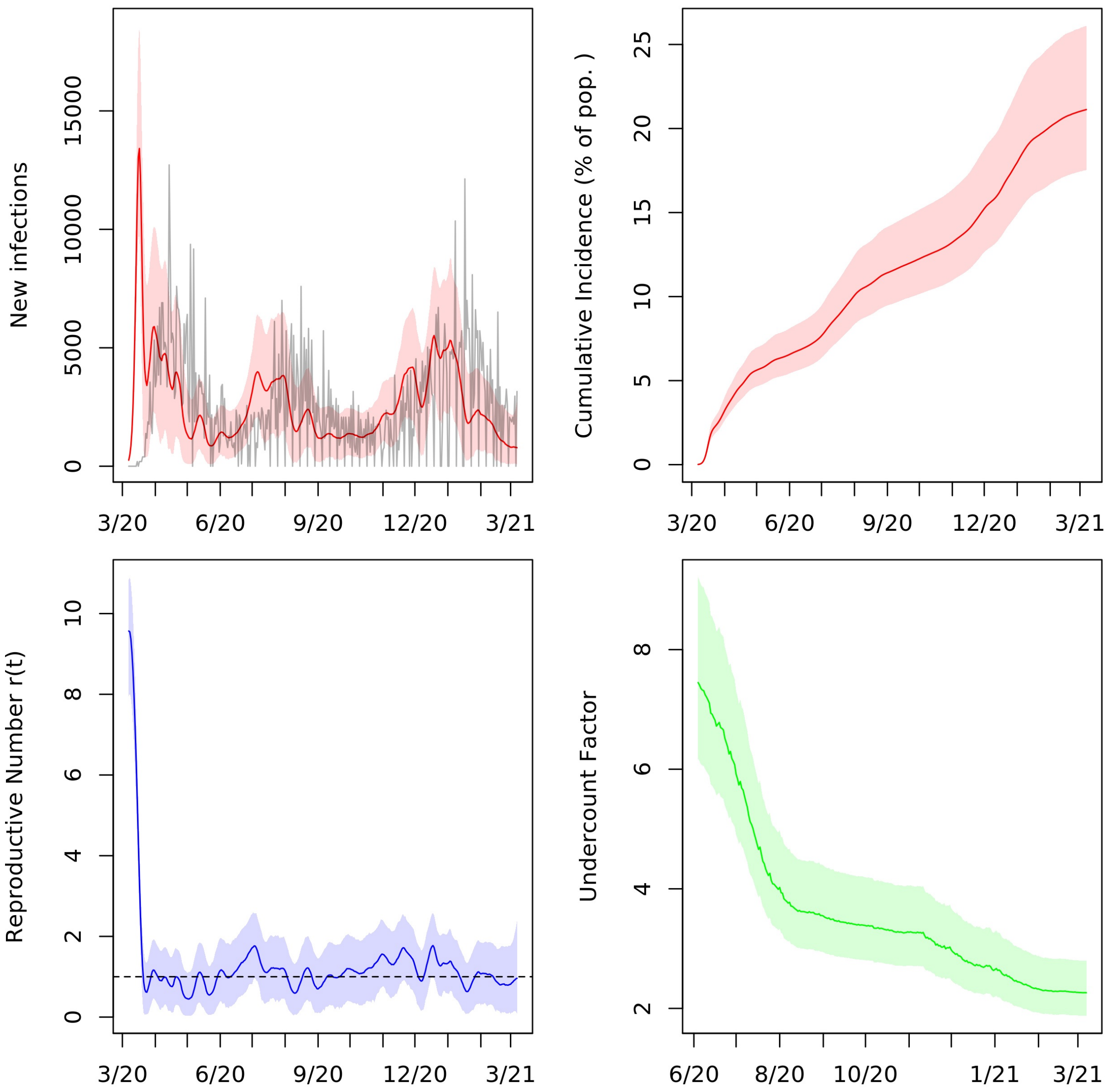

Figure 19: Posterior median and middle 95% intervals for daily new infections, cumulative incidence, $r(t)$, and cumulative undercount from March 2020 to March 2021. In the top left panel, deaths divided by the posterior median IFR are plotted in grey for comparison.

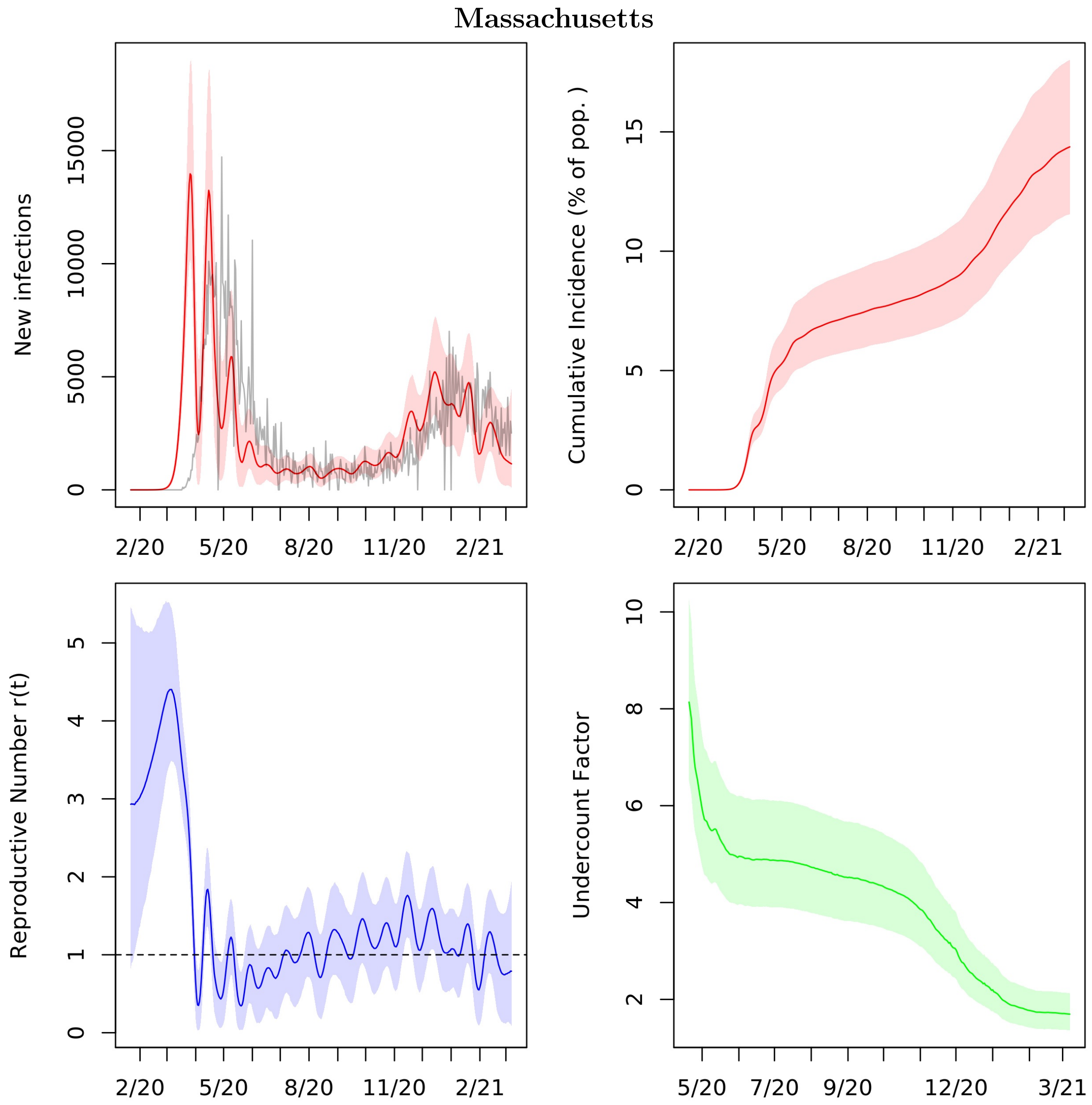

Figure 20: Posterior median and middle 95% intervals for daily new infections, cumulative incidence, $r(t)$, and cumulative undercount from March 2020 to March 2021. In the top left panel, deaths divided by the posterior median IFR are plotted in grey for comparison.

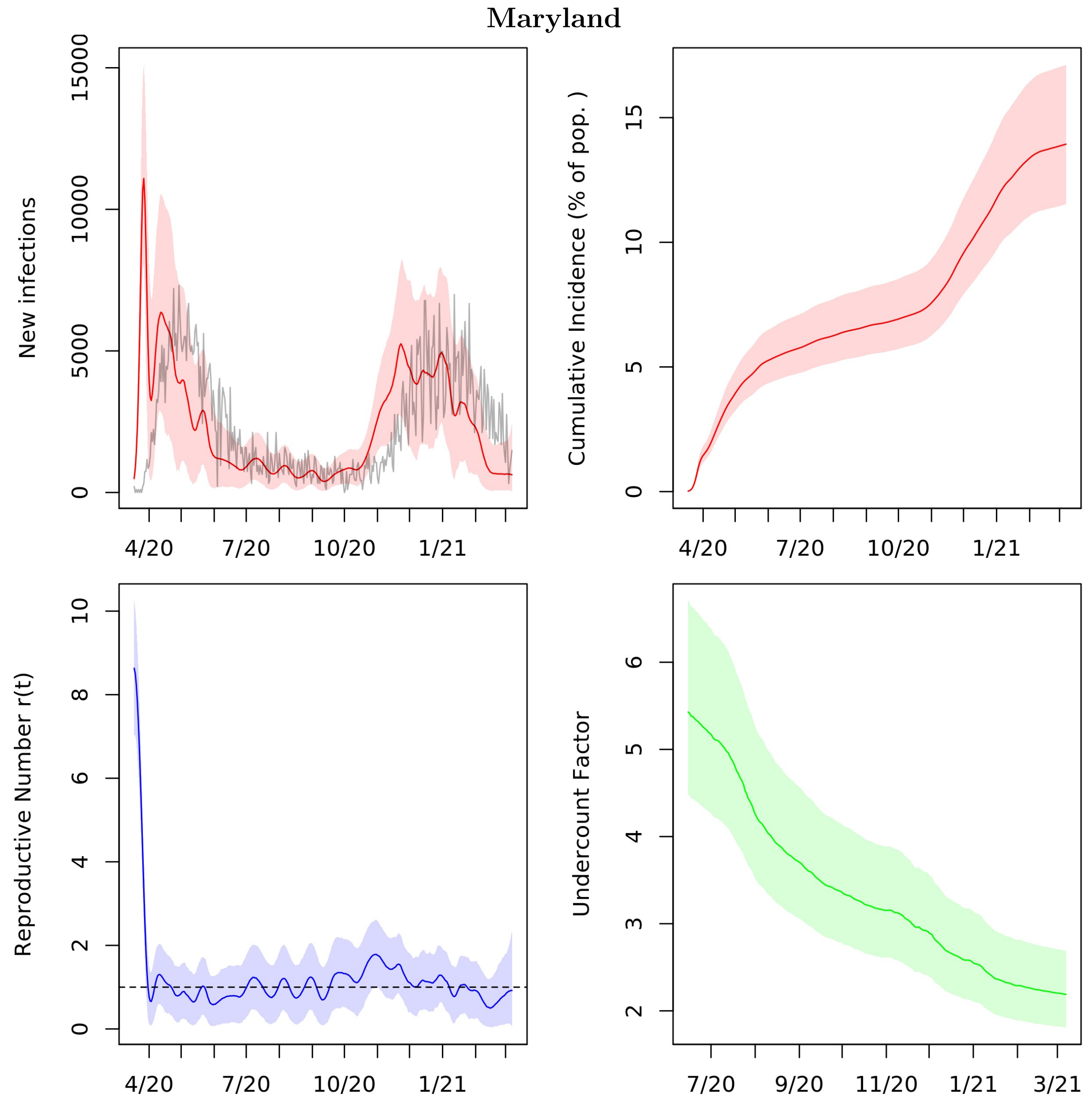

Figure 21: Posterior median and middle 95% intervals for daily new infections, cumulative incidence, $r(t)$, and cumulative undercount from March 2020 to March 2021. In the top left panel, deaths divided by the posterior median IFR are plotted in grey for comparison.

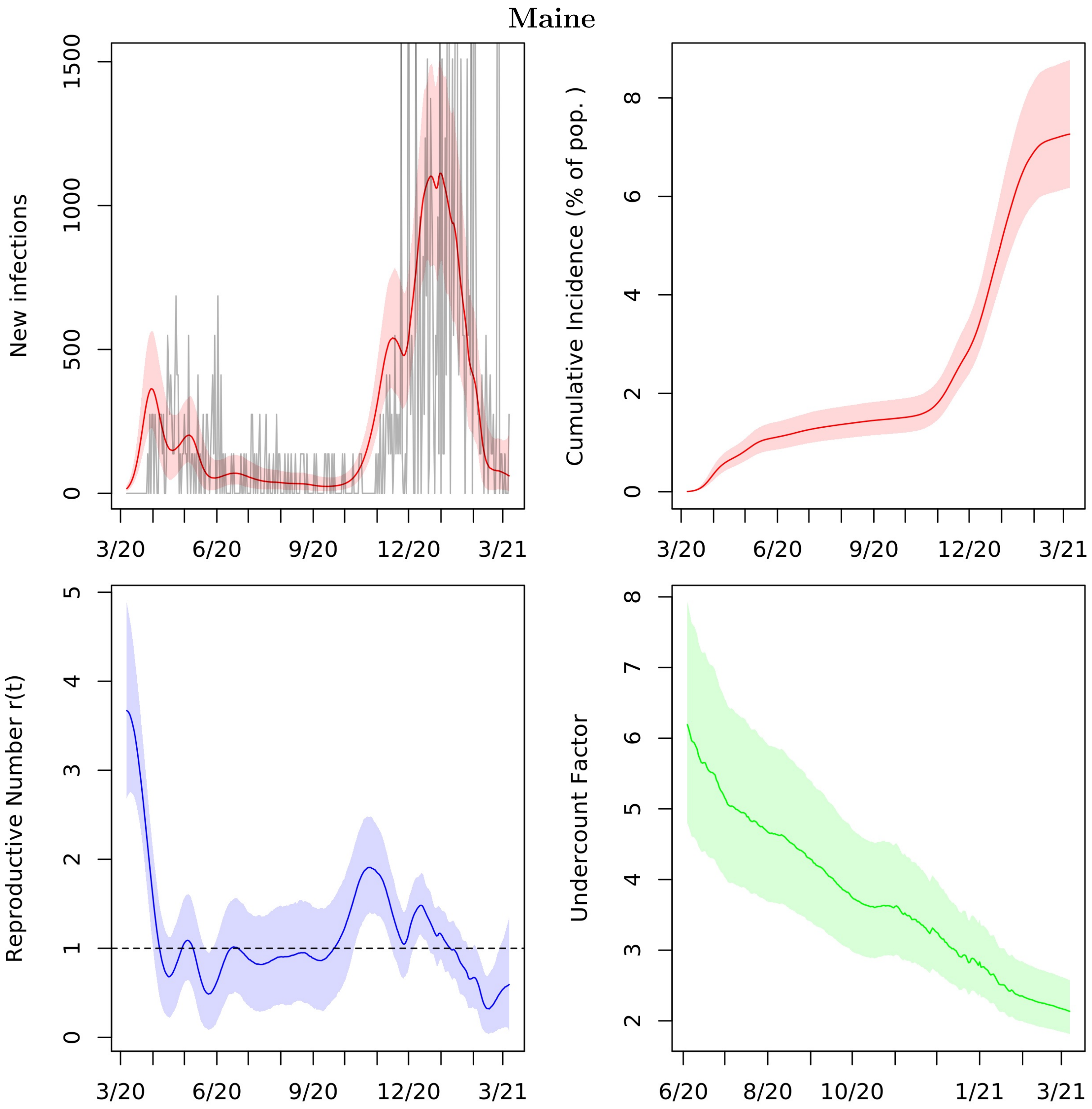

Figure 22: Posterior median and middle 95% intervals for daily new infections, cumulative incidence, $r(t)$, and cumulative undercount from March 2020 to March 2021. In the top left panel, deaths divided by the posterior median IFR are plotted in grey for comparison.

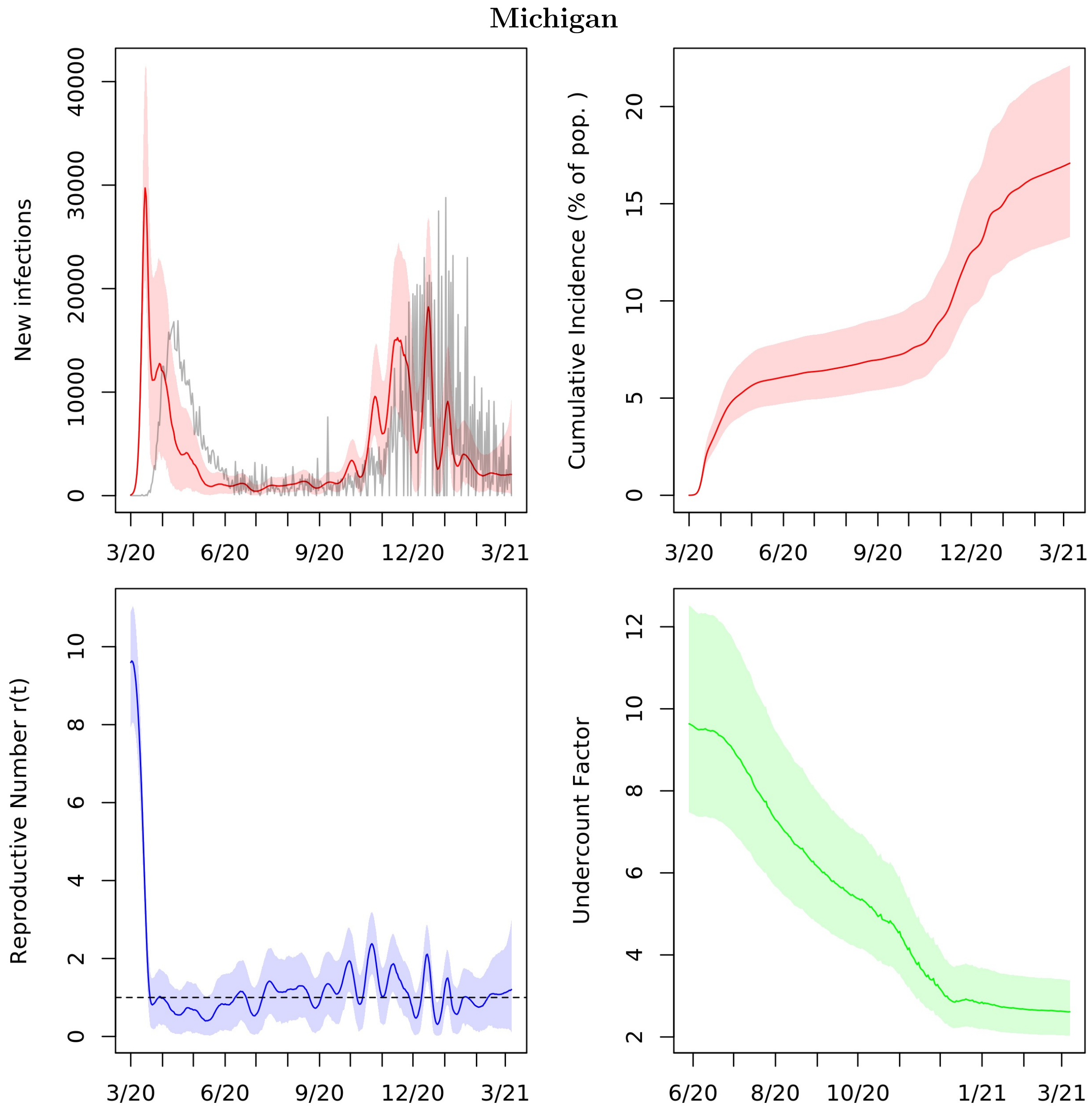

Figure 23: Posterior median and middle 95% intervals for daily new infections, cumulative incidence, $r(t)$, and cumulative undercount from March 2020 to March 2021. In the top left panel, deaths divided by the posterior median IFR are plotted in grey for comparison.

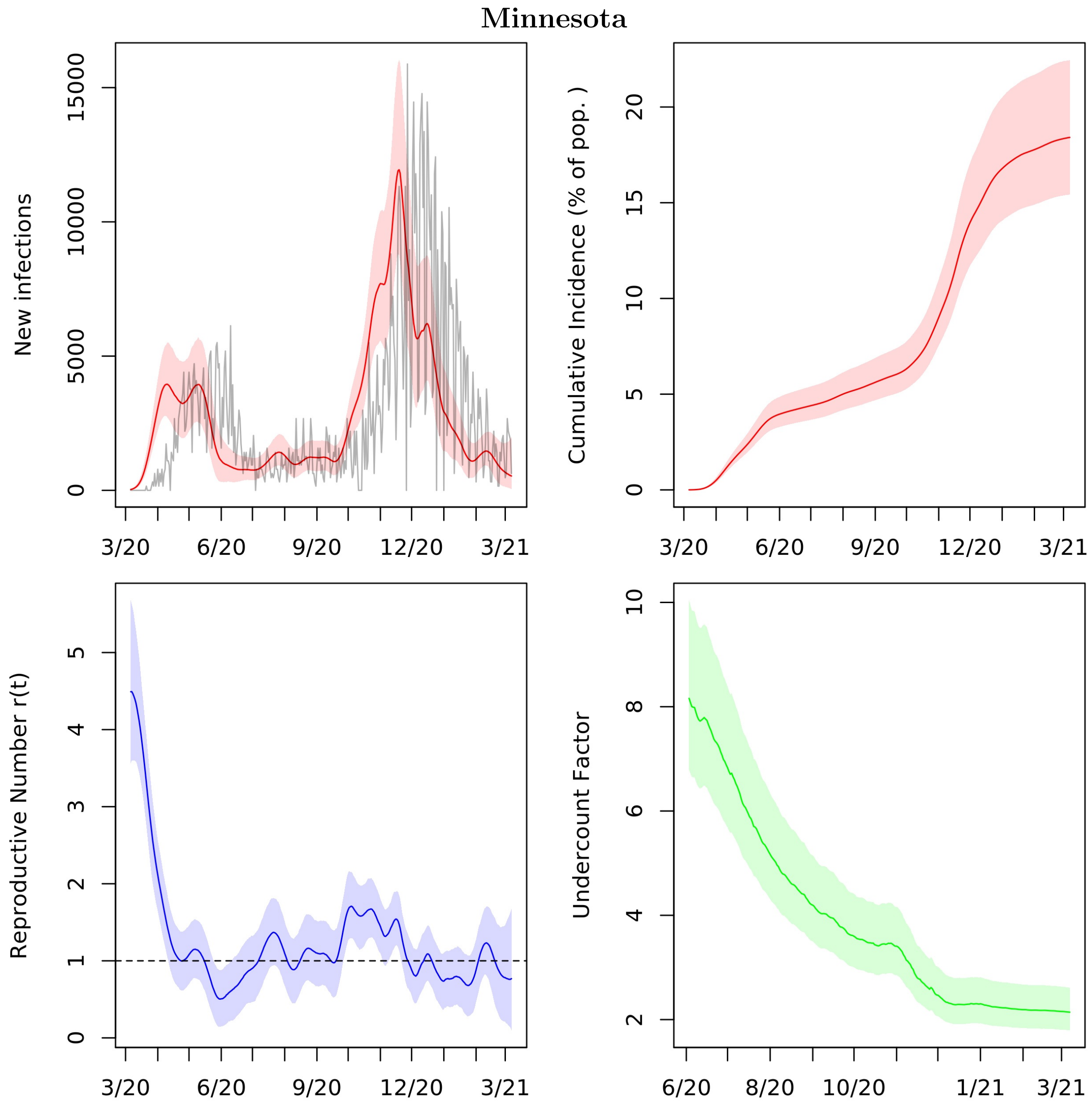

Figure 24: Posterior median and middle 95% intervals for daily new infections, cumulative incidence, $r(t)$, and cumulative undercount from March 2020 to March 2021. In the top left panel, deaths divided by the posterior median IFR are plotted in grey for comparison.

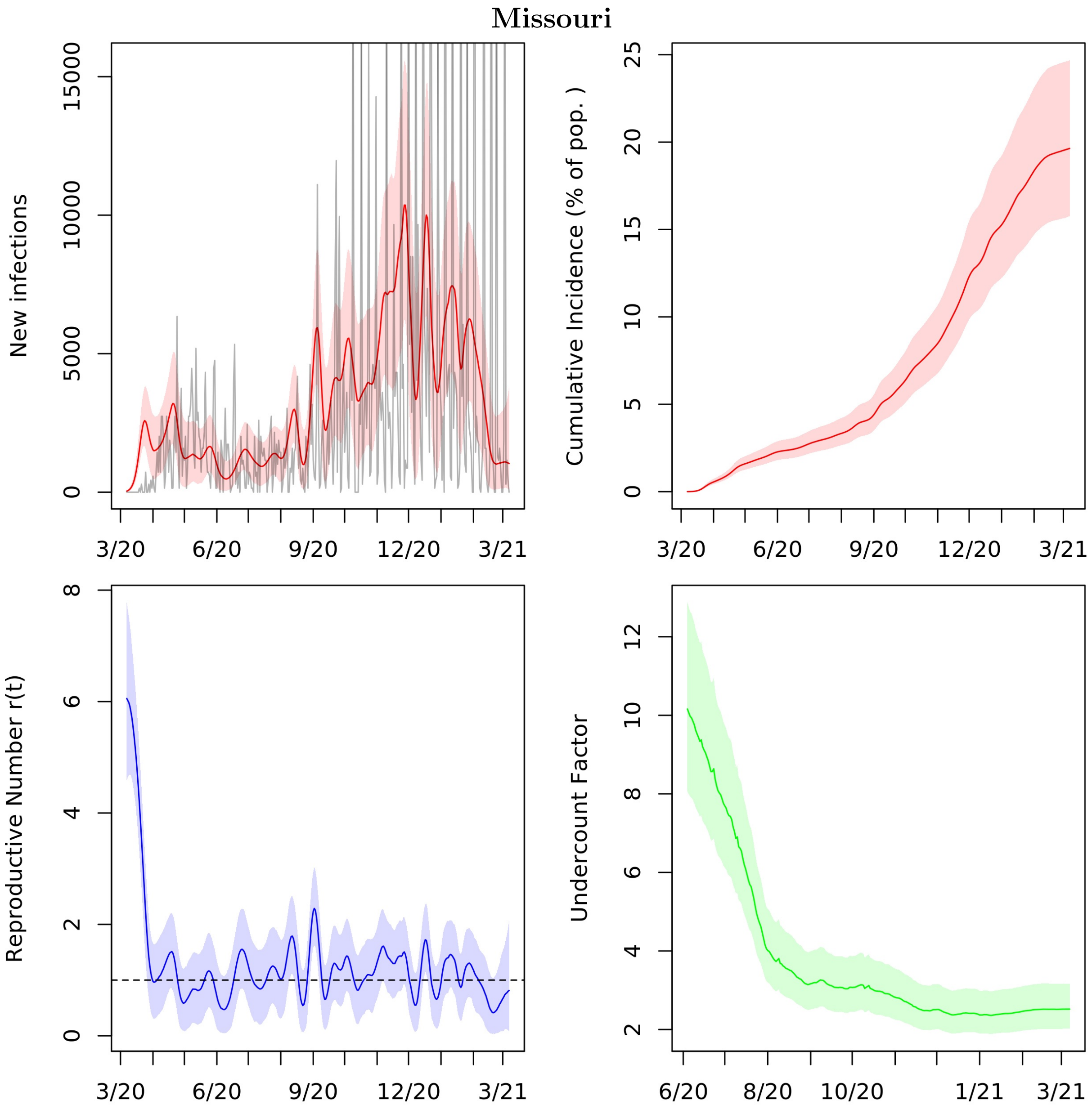

Figure 25: Posterior median and middle 95% intervals for daily new infections, cumulative incidence, $r(t)$, and cumulative undercount from March 2020 to March 2021. In the top left panel, deaths divided by the posterior median IFR are plotted in grey for comparison.

Mississippi

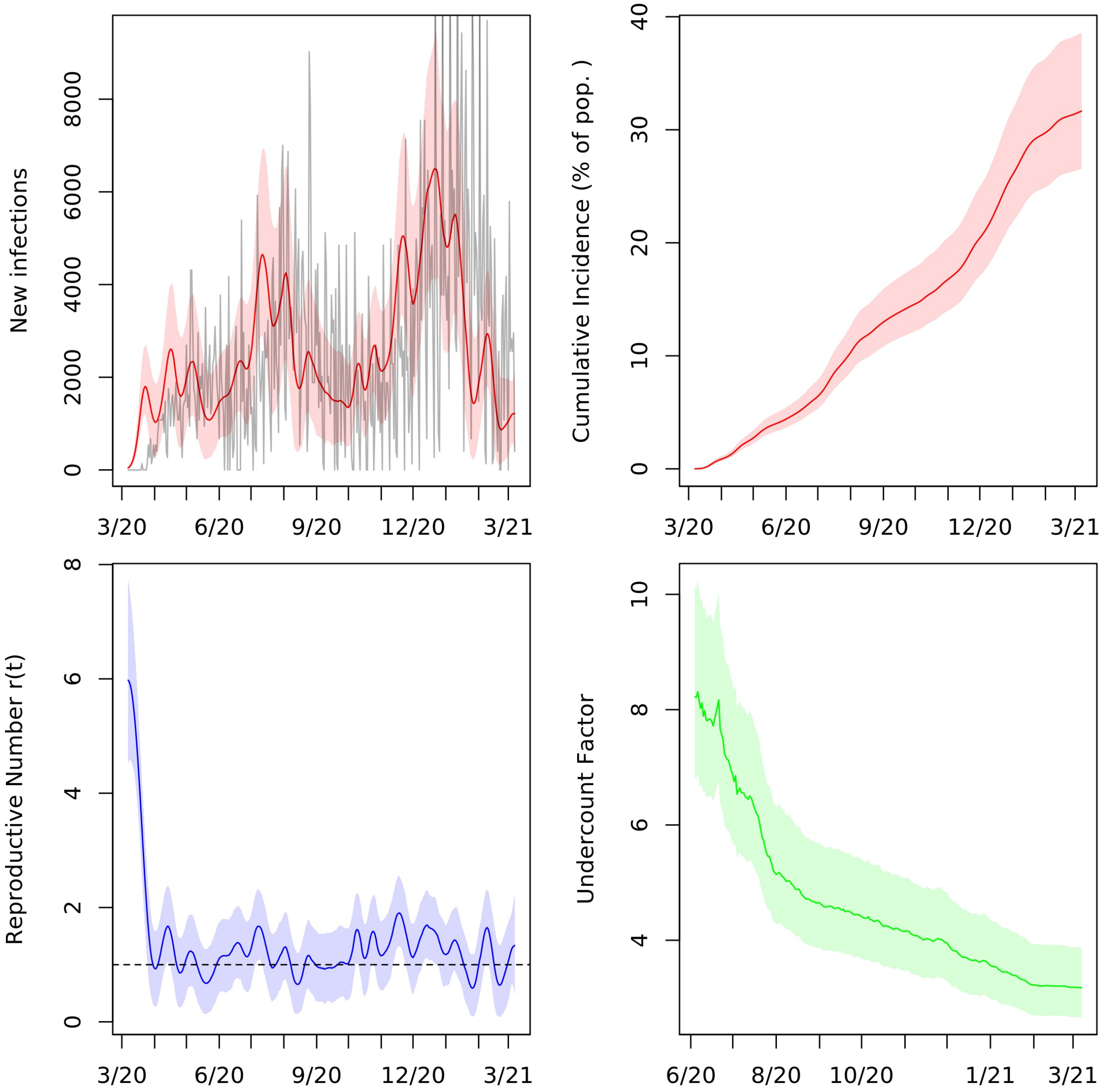

Figure 26: Posterior median and middle 95% intervals for daily new infections, cumulative incidence, $r(t)$, and cumulative undercount from March 2020 to March 2021. In the top left panel, deaths divided by the posterior median IFR are plotted in grey for comparison.

Montana

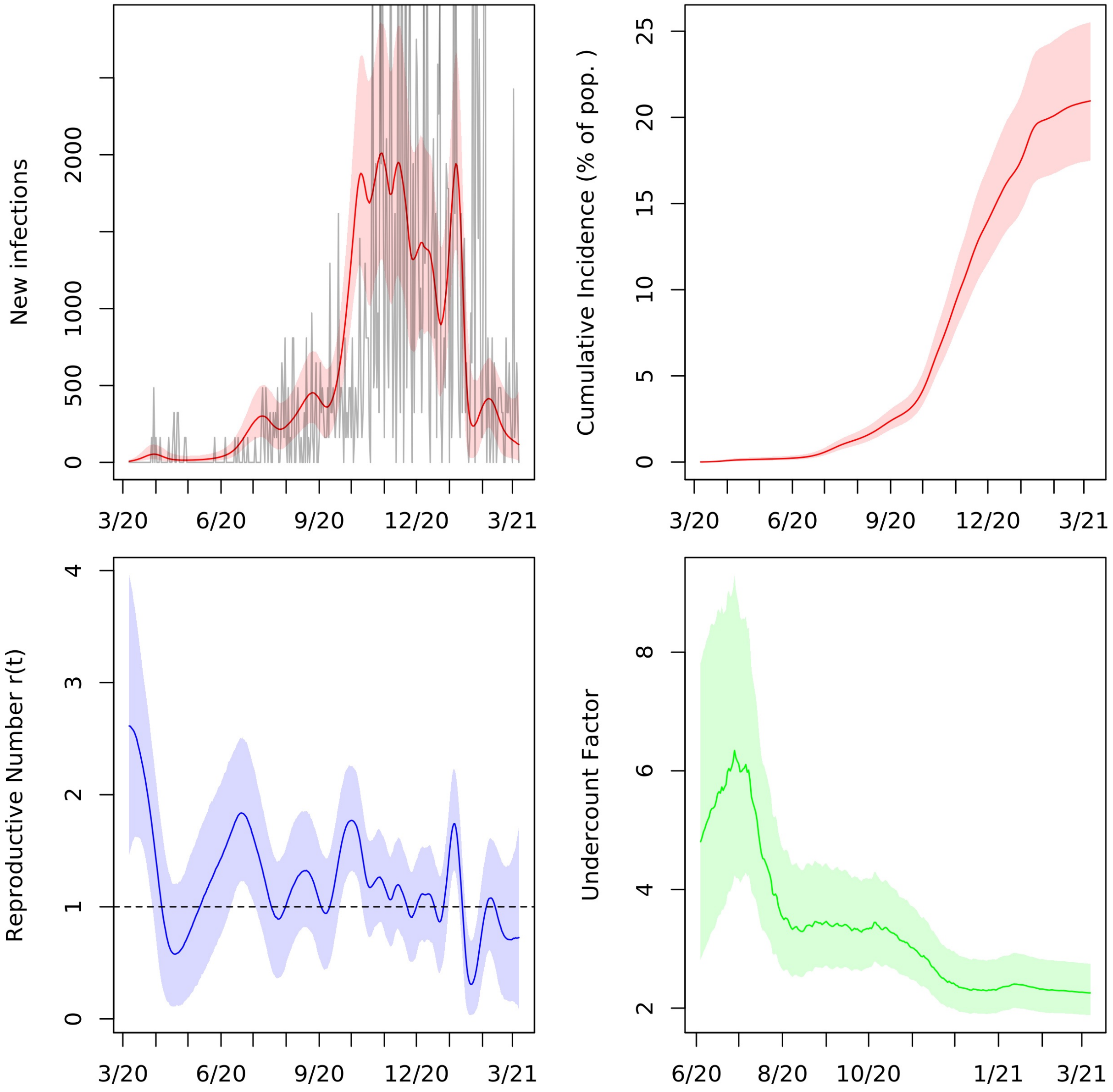

Figure 27: Posterior median and middle 95% intervals for daily new infections, cumulative incidence, $r(t)$, and cumulative undercount from March 2020 to March 2021. In the top left panel, deaths divided by the posterior median IFR are plotted in grey for comparison.

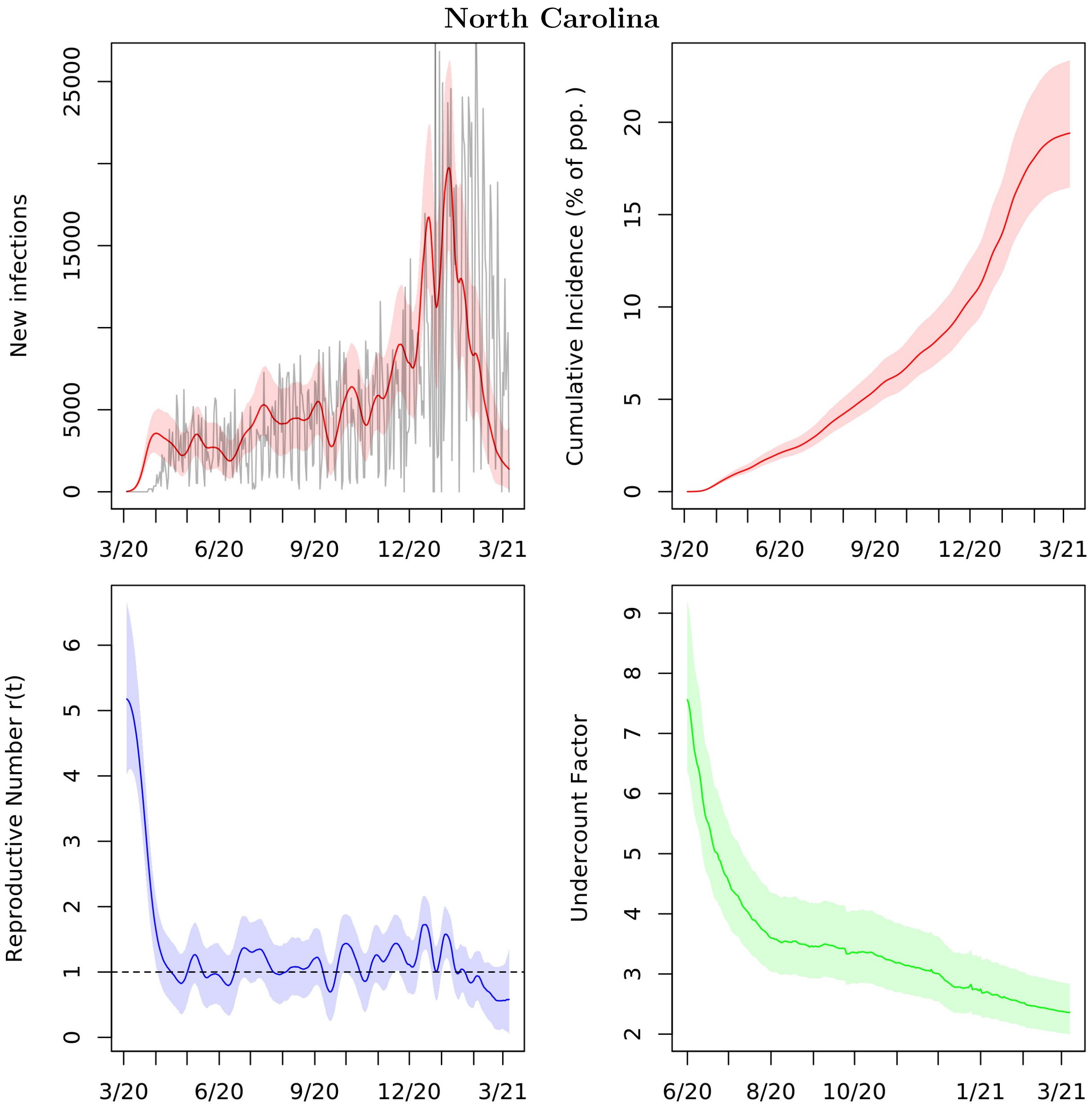

Figure 28: Posterior median and middle 95% intervals for daily new infections, cumulative incidence, $r(t)$, and cumulative undercount from March 2020 to March 2021. In the top left panel, deaths divided by the posterior median IFR are plotted in grey for comparison.

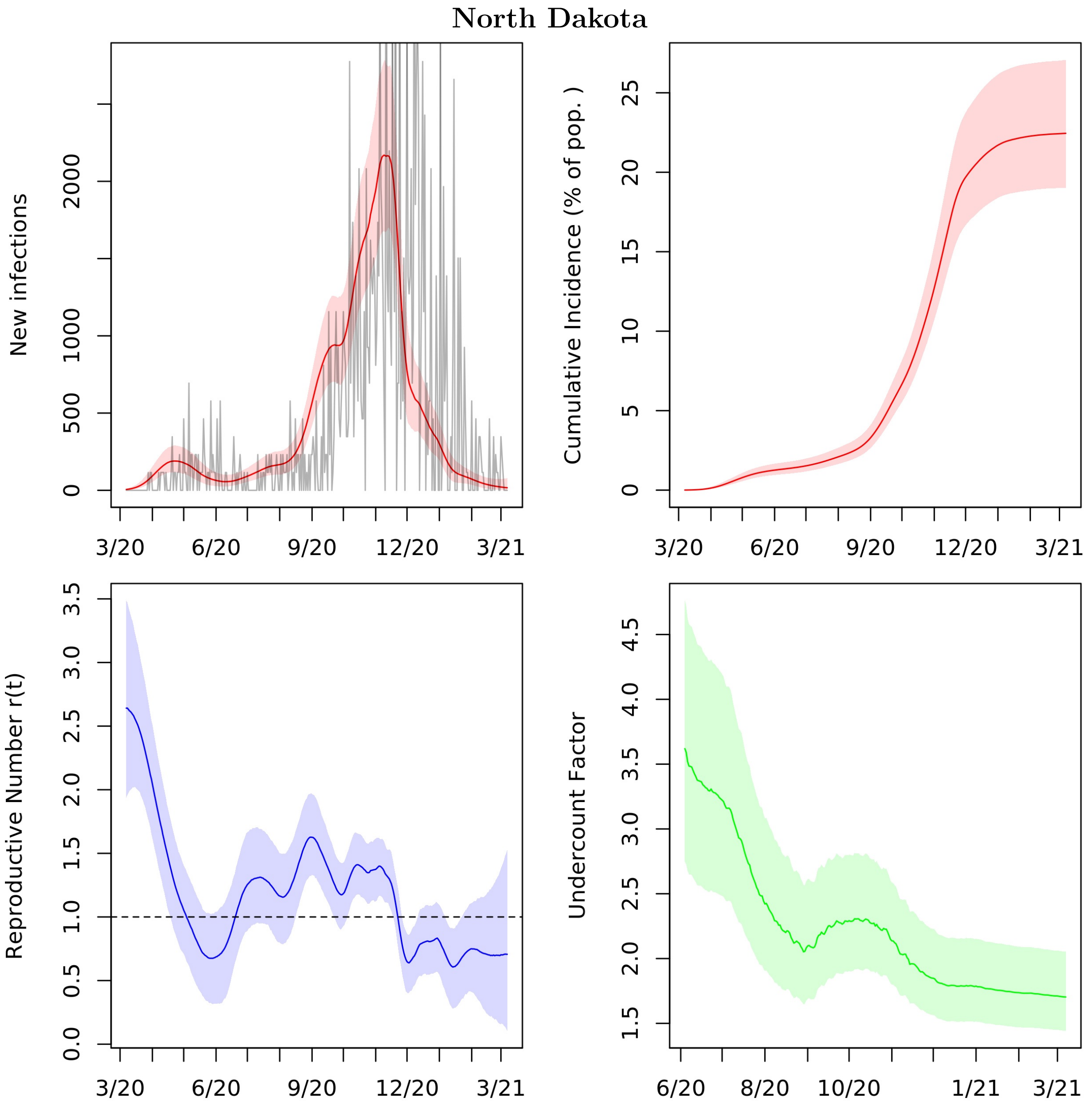

Figure 29: Posterior median and middle 95% intervals for daily new infections, cumulative incidence, $r(t)$, and cumulative undercount from March 2020 to March 2021. In the top left panel, deaths divided by the posterior median IFR are plotted in grey for comparison.

Nebraska

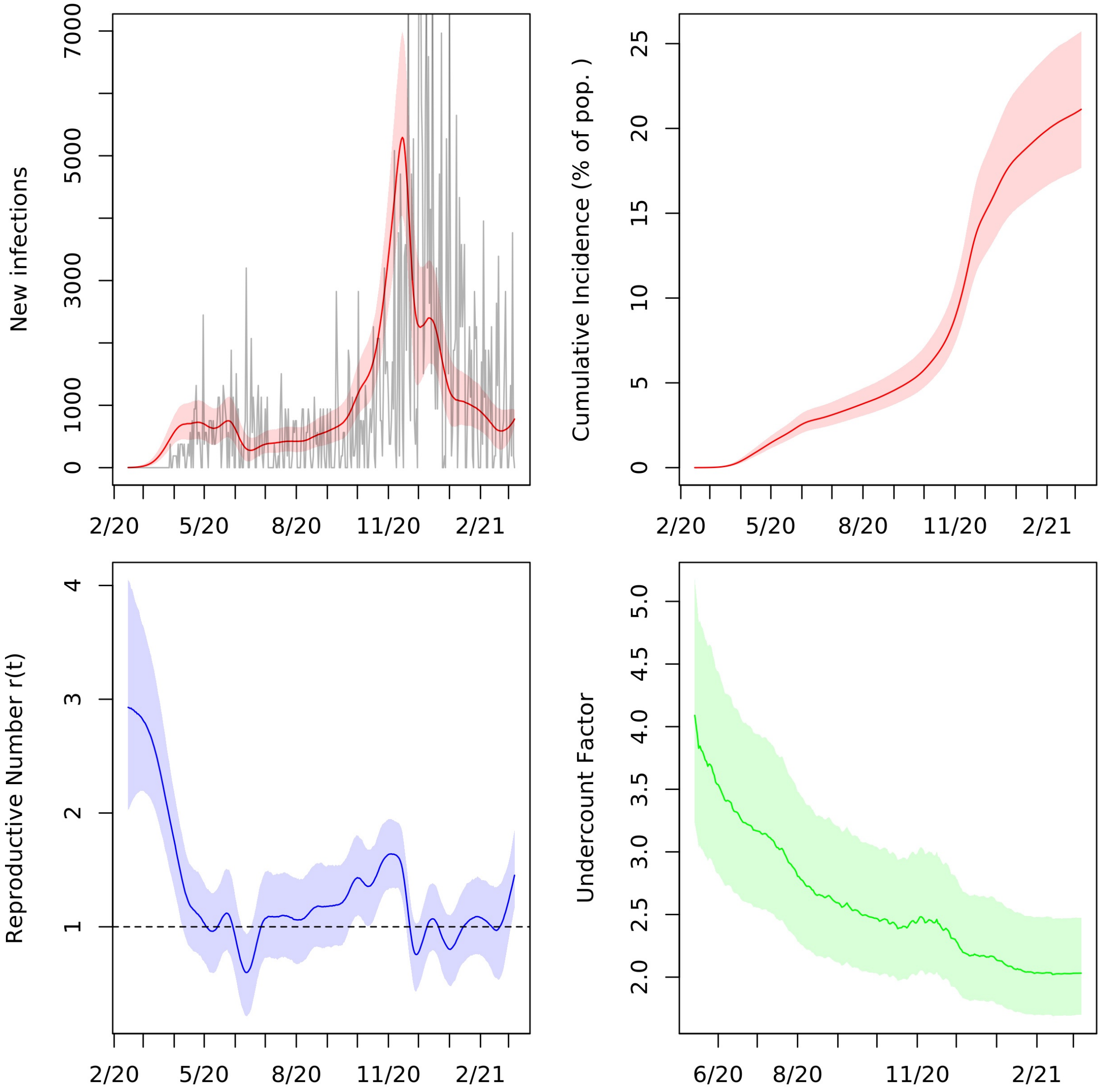

Figure 30: Posterior median and middle 95% intervals for daily new infections, cumulative incidence, $r(t)$, and cumulative undercount from March 2020 to March 2021. In the top left panel, deaths divided by the posterior median IFR are plotted in grey for comparison.

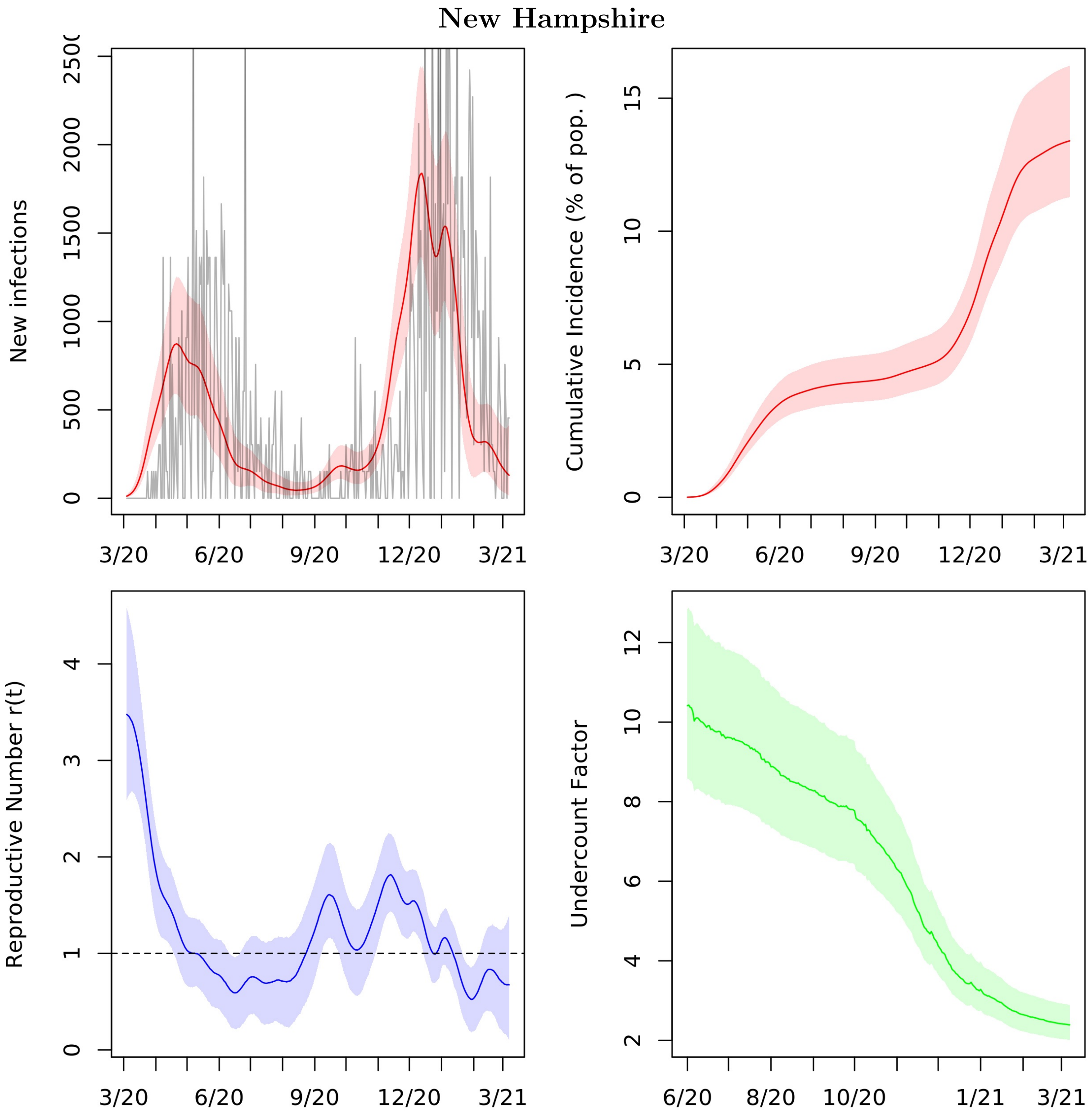

Figure 31: Posterior median and middle 95% intervals for daily new infections, cumulative incidence, $r(t)$, and cumulative undercount from March 2020 to March 2021. In the top left panel, deaths divided by the posterior median IFR are plotted in grey for comparison.

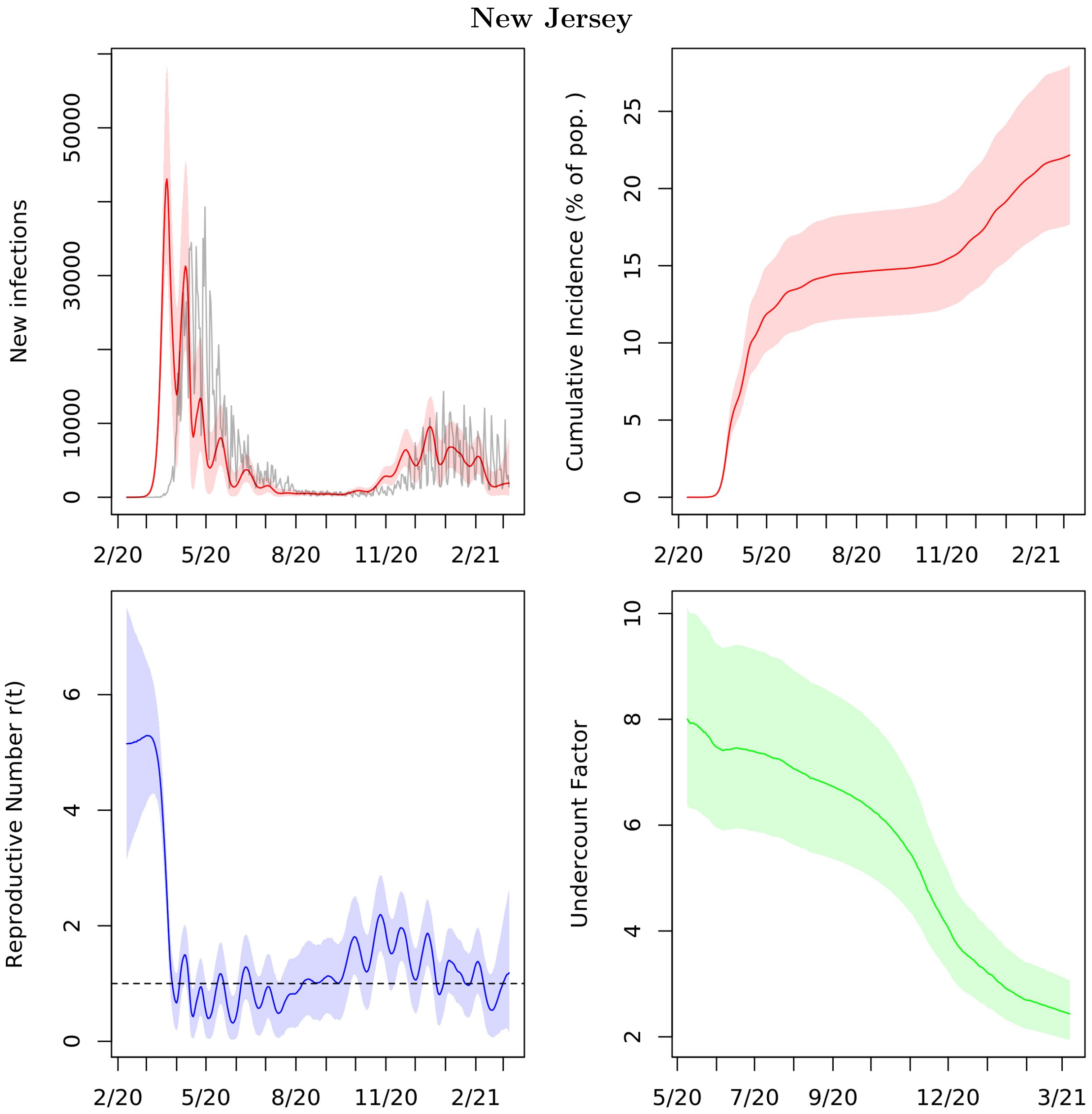

Figure 32: Posterior median and middle 95% intervals for daily new infections, cumulative incidence, $r(t)$, and cumulative undercount from March 2020 to March 2021. In the top left panel, deaths divided by the posterior median IFR are plotted in grey for comparison.

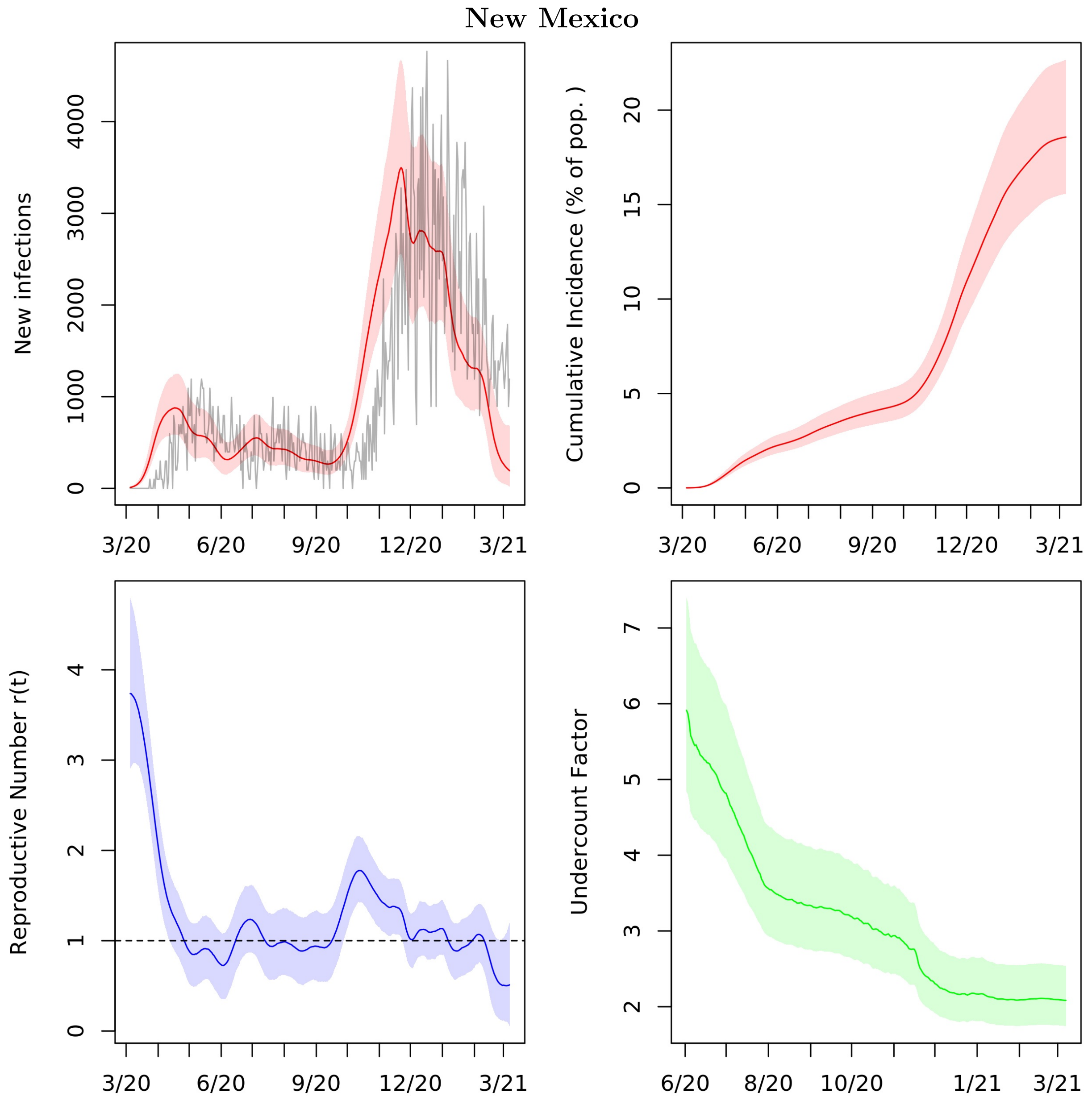

Figure 33: Posterior median and middle 95% intervals for daily new infections, cumulative incidence, $r(t)$, and cumulative undercount from March 2020 to March 2021. In the top left panel, deaths divided by the posterior median IFR are plotted in grey for comparison.

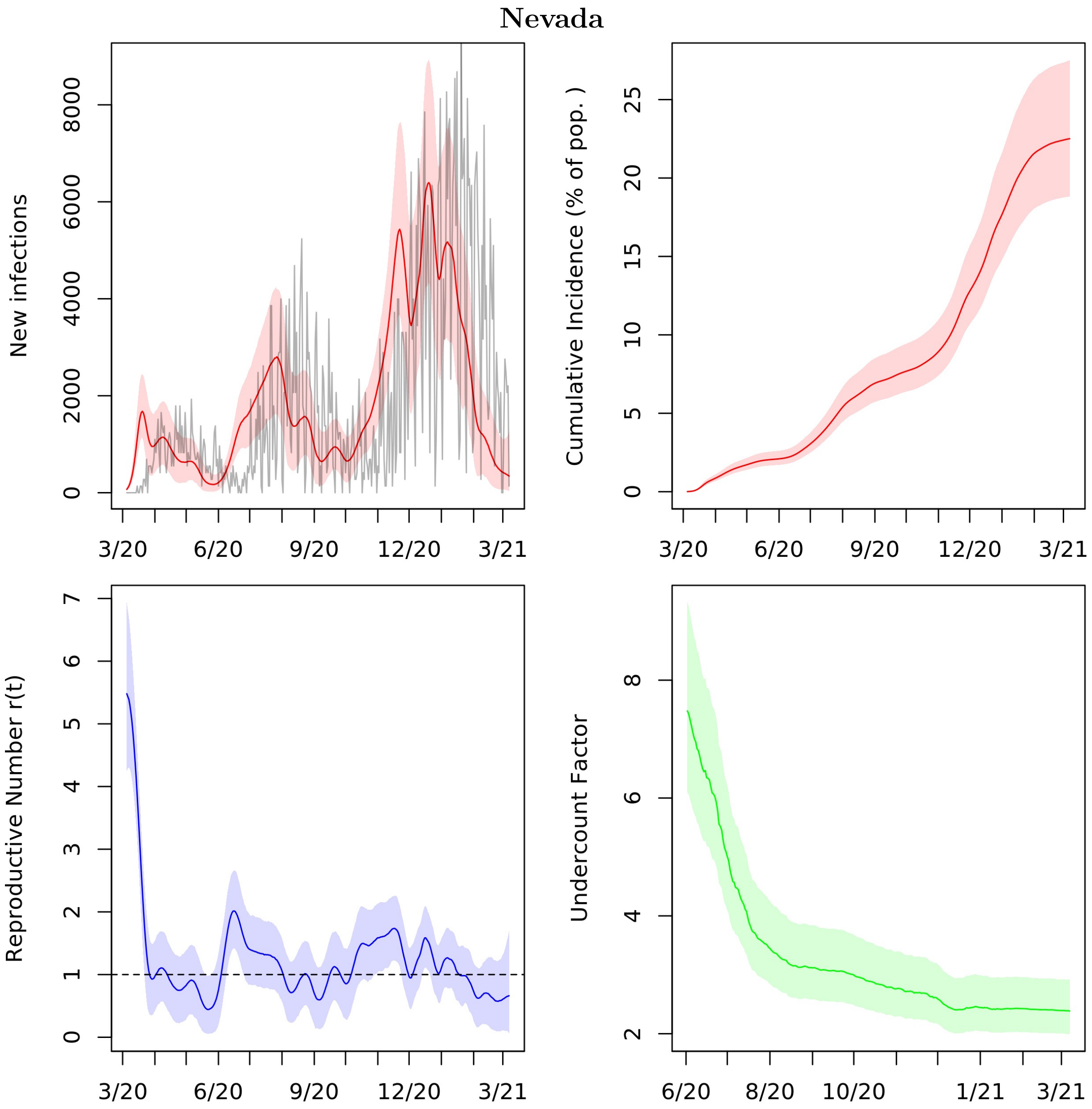

Figure 34: Posterior median and middle 95% intervals for daily new infections, cumulative incidence, $r(t)$, and cumulative undercount from March 2020 to March 2021. In the top left panel, deaths divided by the posterior median IFR are plotted in grey for comparison.

New York

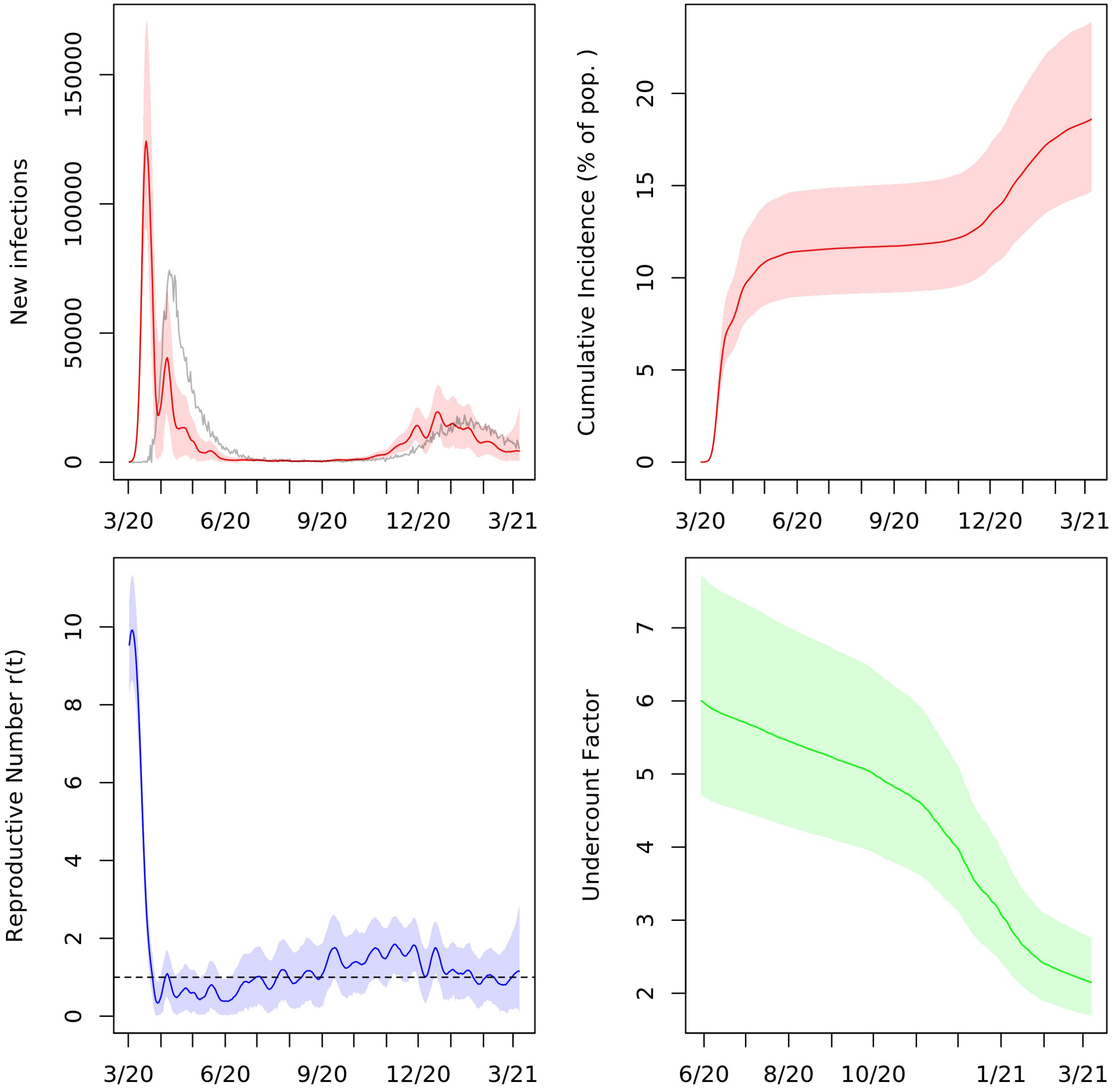

Figure 35: Posterior median and middle 95% intervals for daily new infections, cumulative incidence, $r(t)$, and cumulative undercount from March 2020 to March 2021. In the top left panel, deaths divided by the posterior median IFR are plotted in grey for comparison.

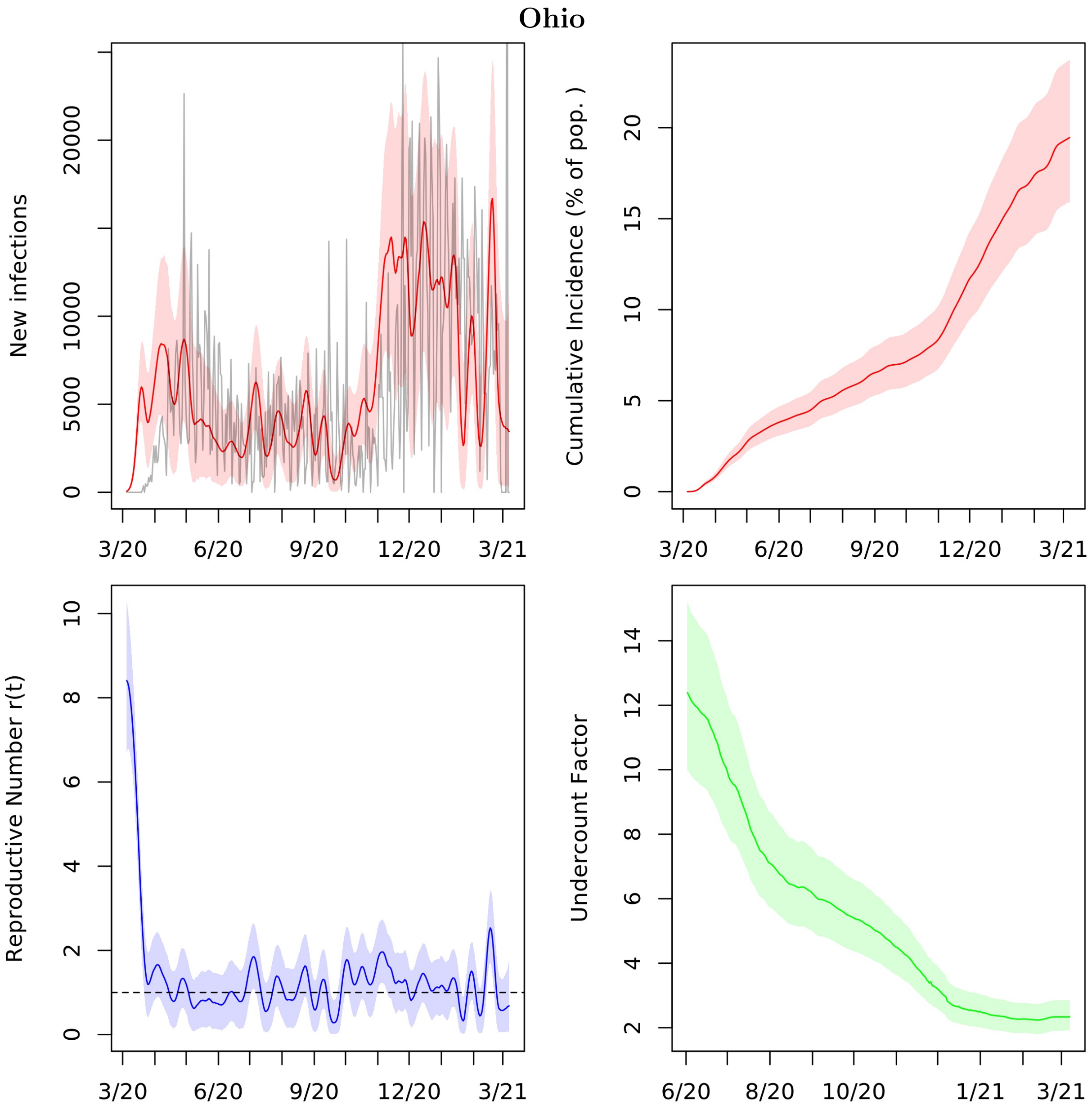

Figure 36: Posterior median and middle 95% intervals for daily new infections, cumulative incidence, $r(t)$, and cumulative undercount from March 2020 to March 2021. In the top left panel, deaths divided by the posterior median IFR are plotted in grey for comparison.

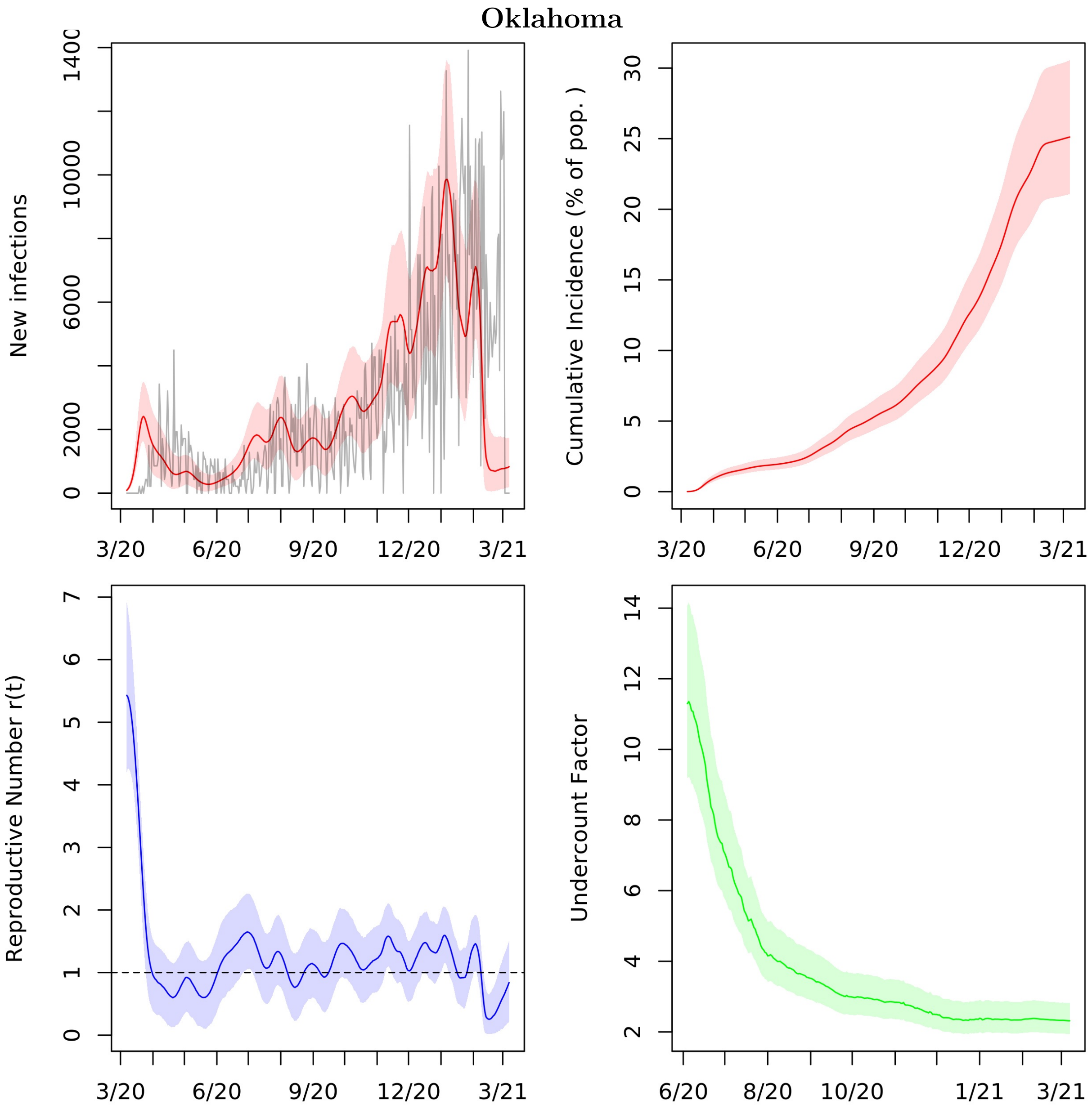

Figure 37: Posterior median and middle 95% intervals for daily new infections, cumulative incidence, $r(t)$, and cumulative undercount from March 2020 to March 2021. In the top left panel, deaths divided by the posterior median IFR are plotted in grey for comparison.

Oregon

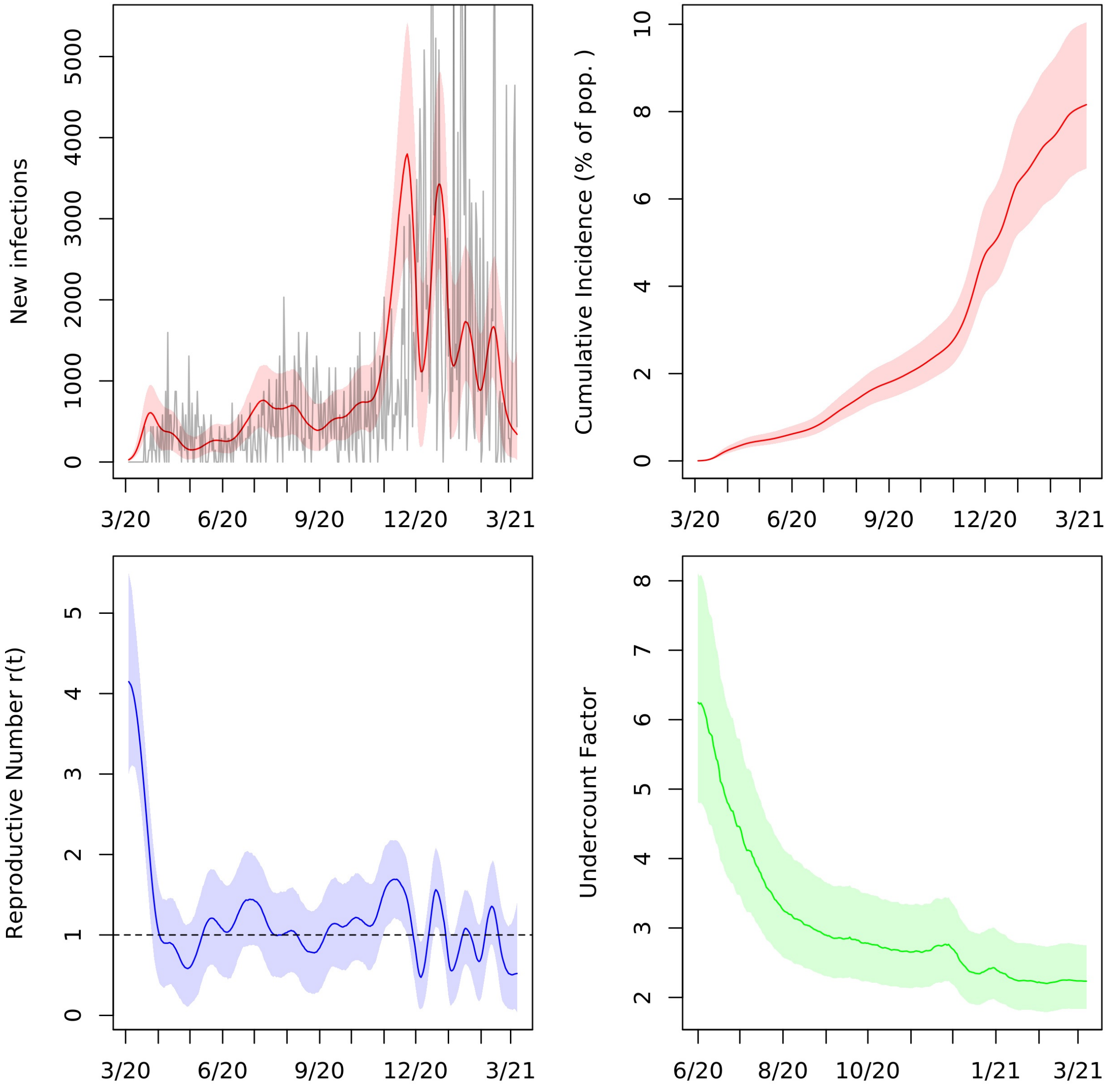

Figure 38: Posterior median and middle 95% intervals for daily new infections, cumulative incidence, $r(t)$, and cumulative undercount from March 2020 to March 2021. In the top left panel, deaths divided by the posterior median IFR are plotted in grey for comparison.

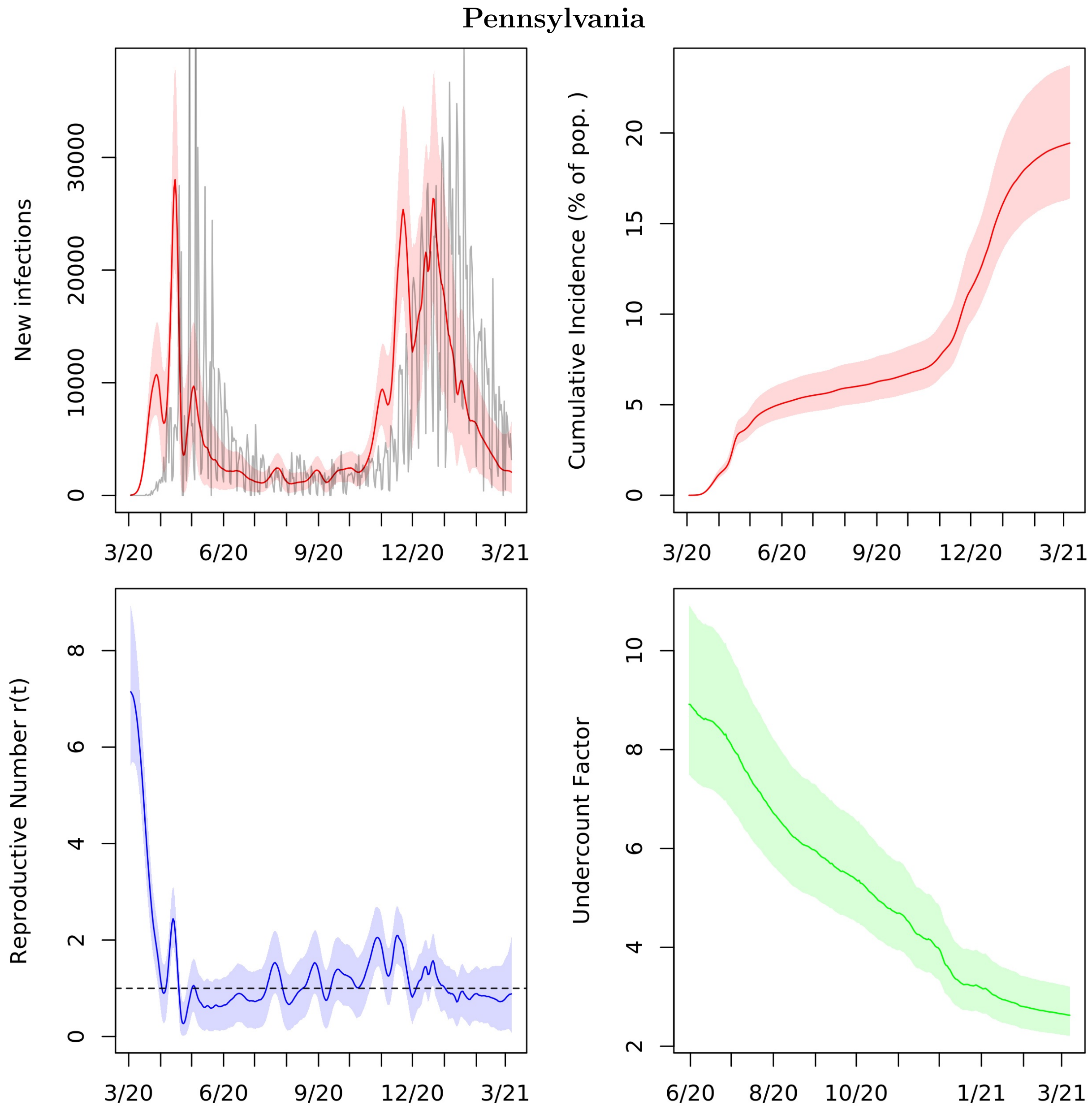

Figure 39: Posterior median and middle 95% intervals for daily new infections, cumulative incidence, $r(t)$, and cumulative undercount from March 2020 to March 2021. In the top left panel, deaths divided by the posterior median IFR are plotted in grey for comparison.

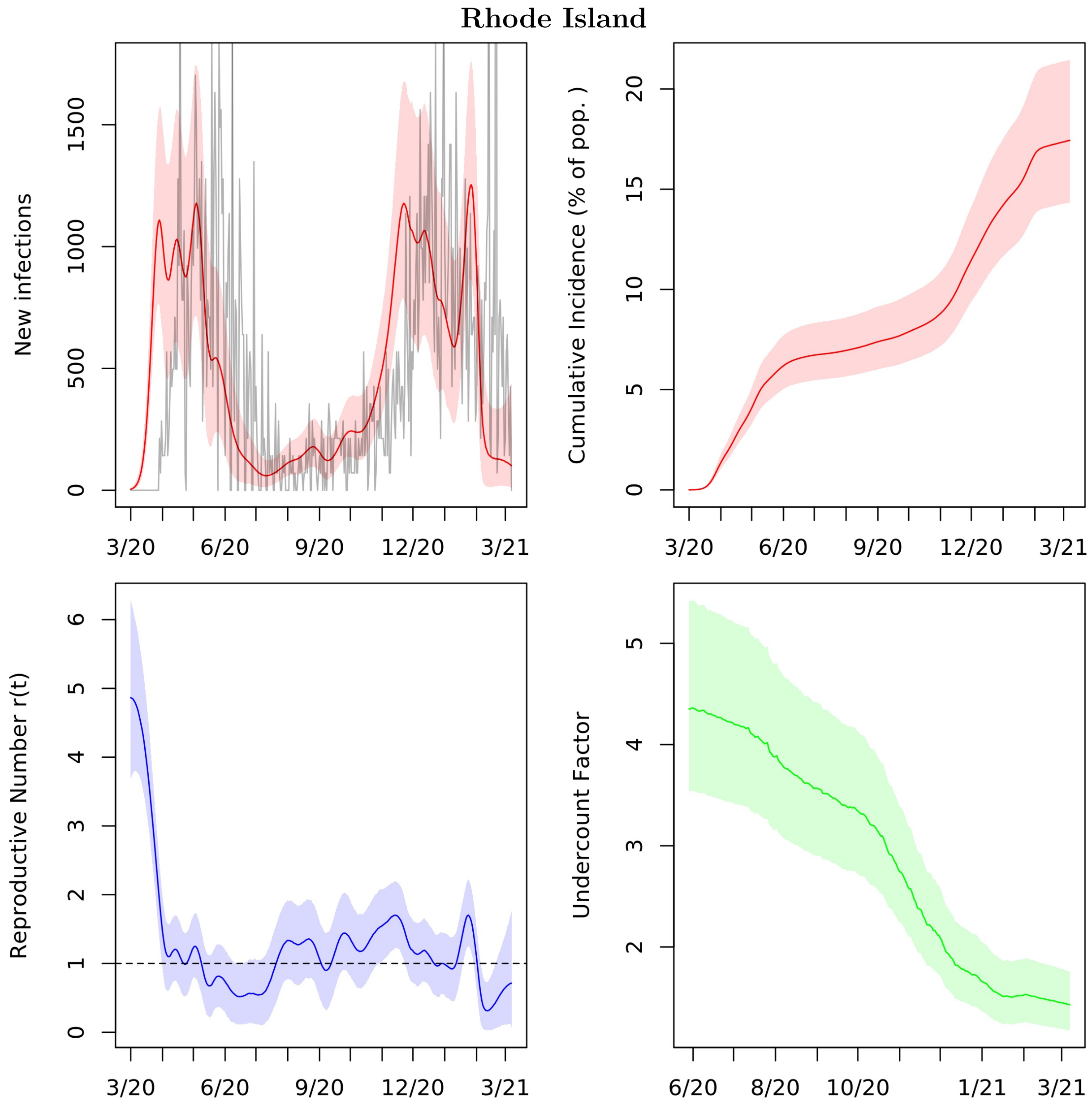

Figure 40: Posterior median and middle 95% intervals for daily new infections, cumulative incidence, $r(t)$, and cumulative undercount from March 2020 to March 2021. In the top left panel, deaths divided by the posterior median IFR are plotted in grey for comparison.

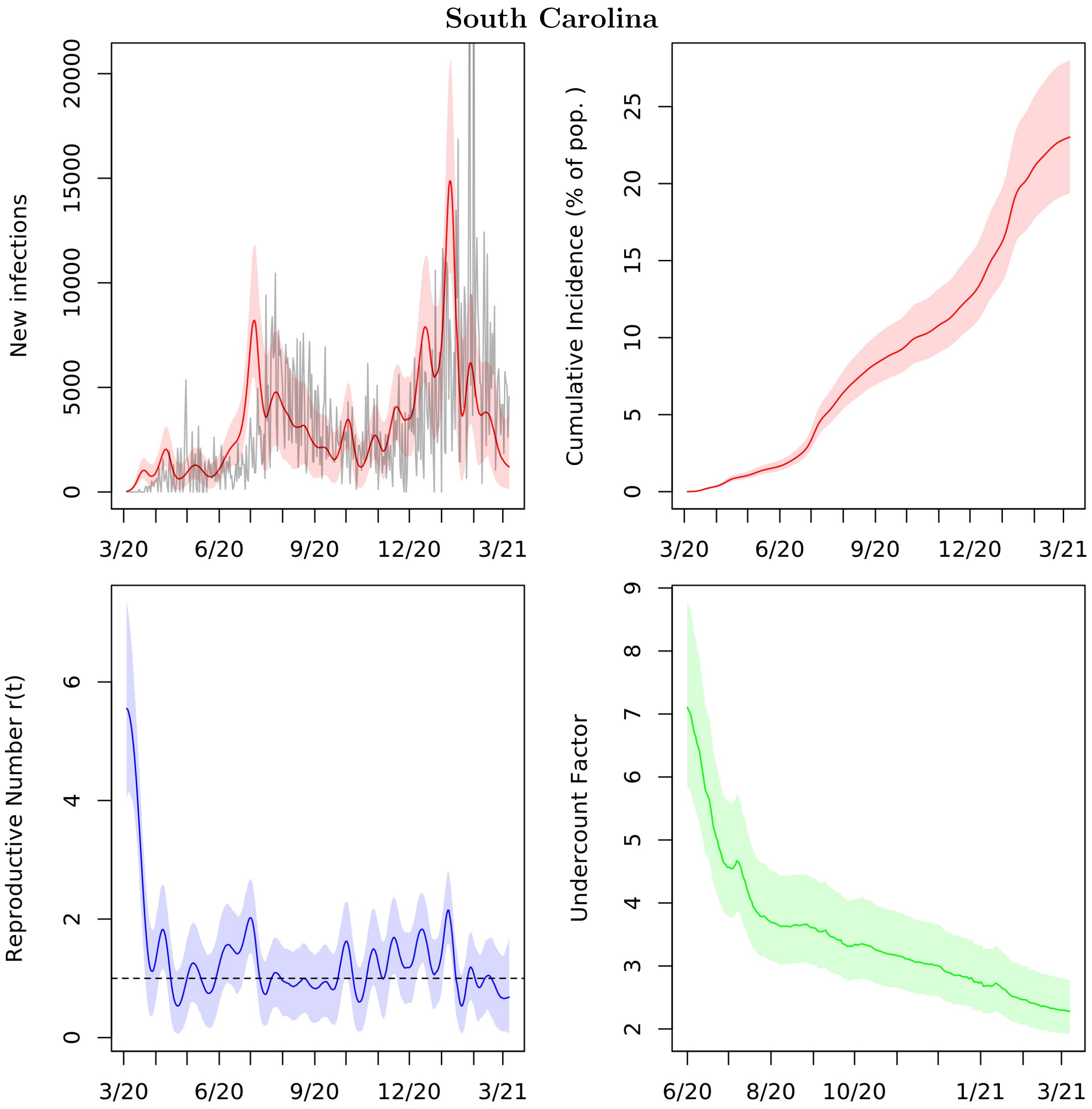

Figure 41: Posterior median and middle 95% intervals for daily new infections, cumulative incidence, $r(t)$, and cumulative undercount from March 2020 to March 2021. In the top left panel, deaths divided by the posterior median IFR are plotted in grey for comparison.

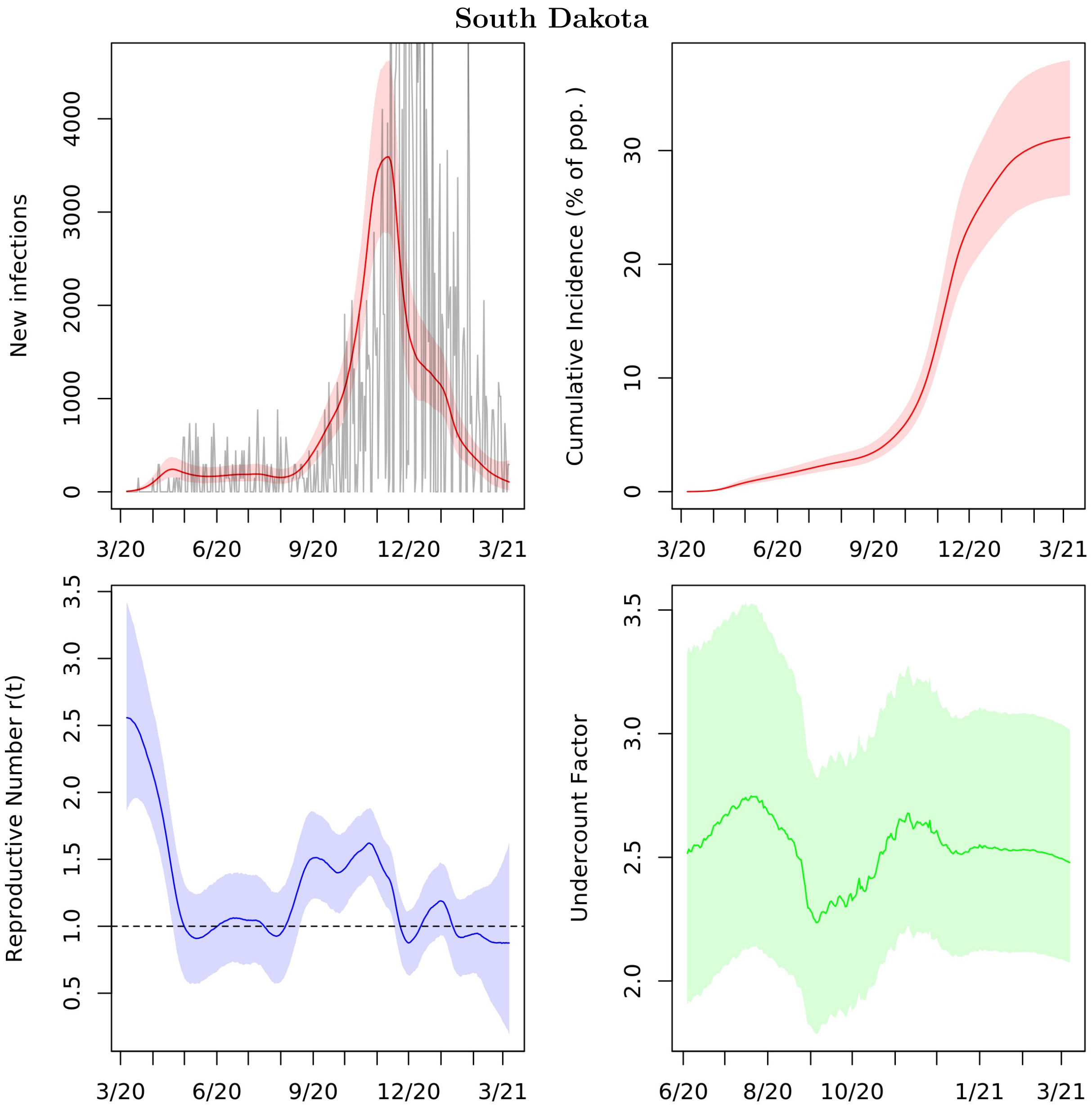

Figure 42: Posterior median and middle 95% intervals for daily new infections, cumulative incidence, $r(t)$, and cumulative undercount from March 2020 to March 2021. In the top left panel, deaths divided by the posterior median IFR are plotted in grey for comparison.

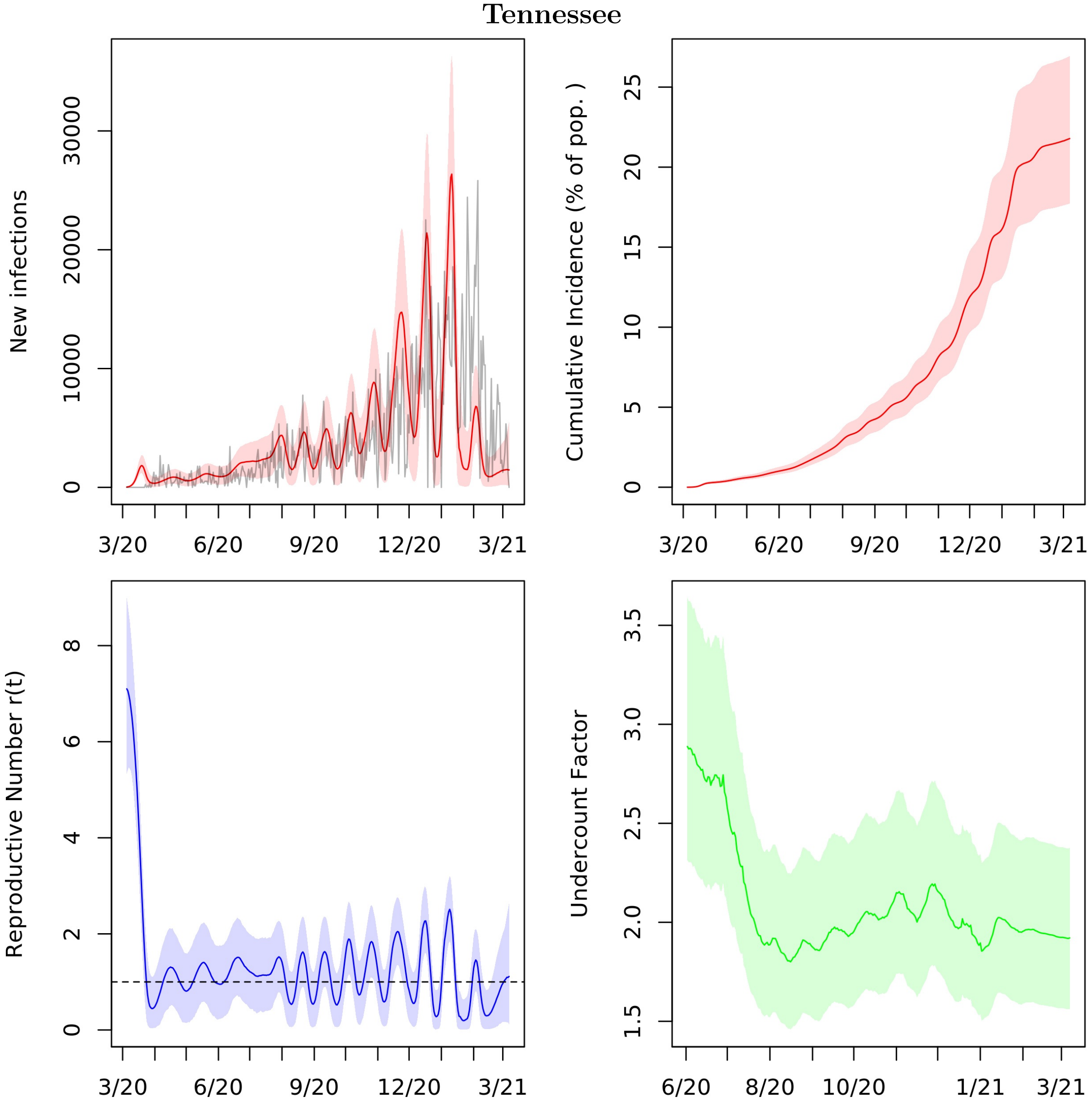

Figure 43: Posterior median and middle 95% intervals for daily new infections, cumulative incidence, $r(t)$, and cumulative undercount from March 2020 to March 2021. In the top left panel, deaths divided by the posterior median IFR are plotted in grey for comparison.

Texas

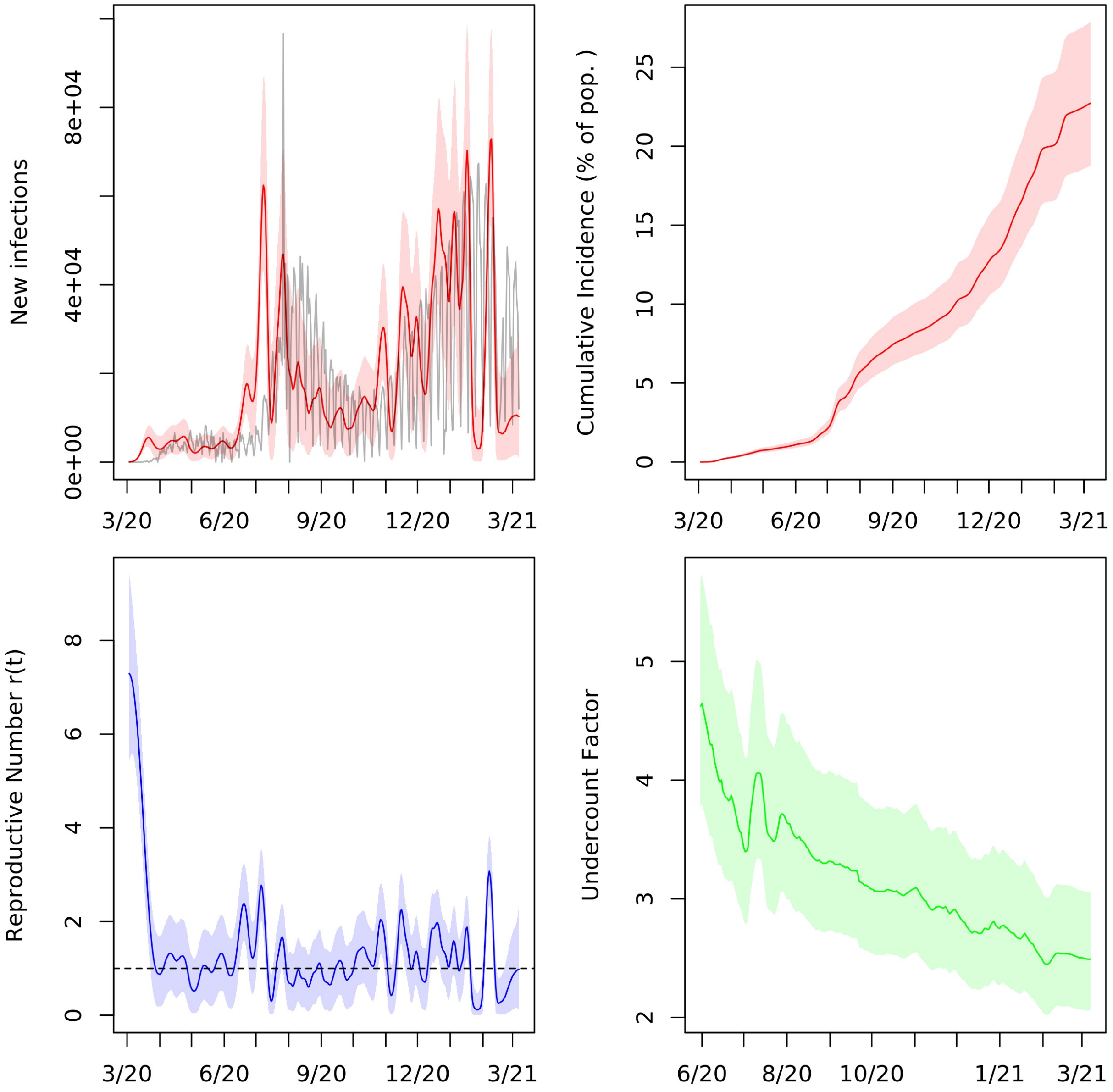

Figure 44: Posterior median and middle 95% intervals for daily new infections, cumulative incidence, $r(t)$, and cumulative undercount from March 2020 to March 2021. In the top left panel, deaths divided by the posterior median IFR are plotted in grey for comparison.

Utah

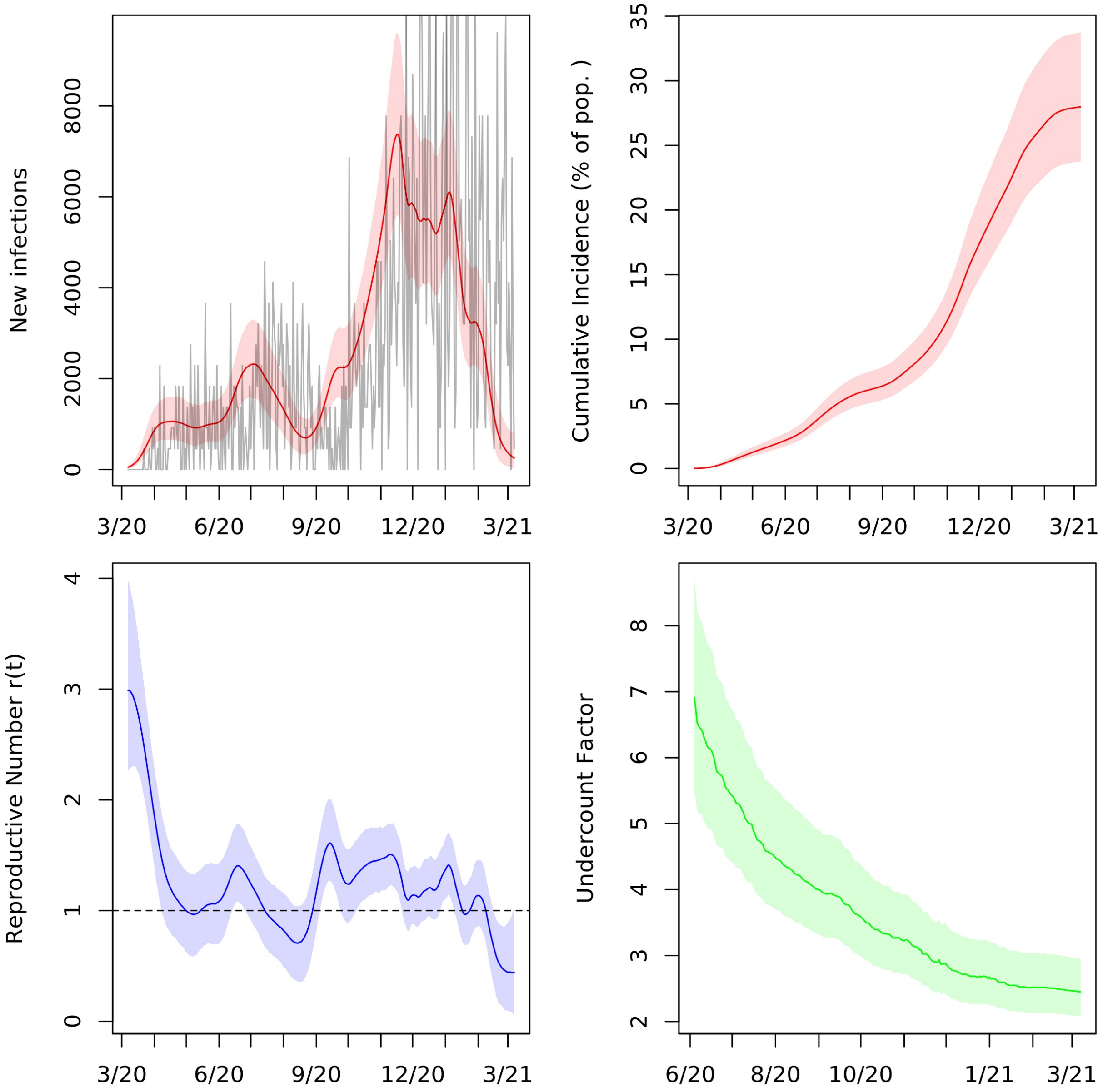

Figure 45: Posterior median and middle 95% intervals for daily new infections, cumulative incidence, $r(t)$, and cumulative undercount from March 2020 to March 2021. In the top left panel, deaths divided by the posterior median IFR are plotted in grey for comparison.

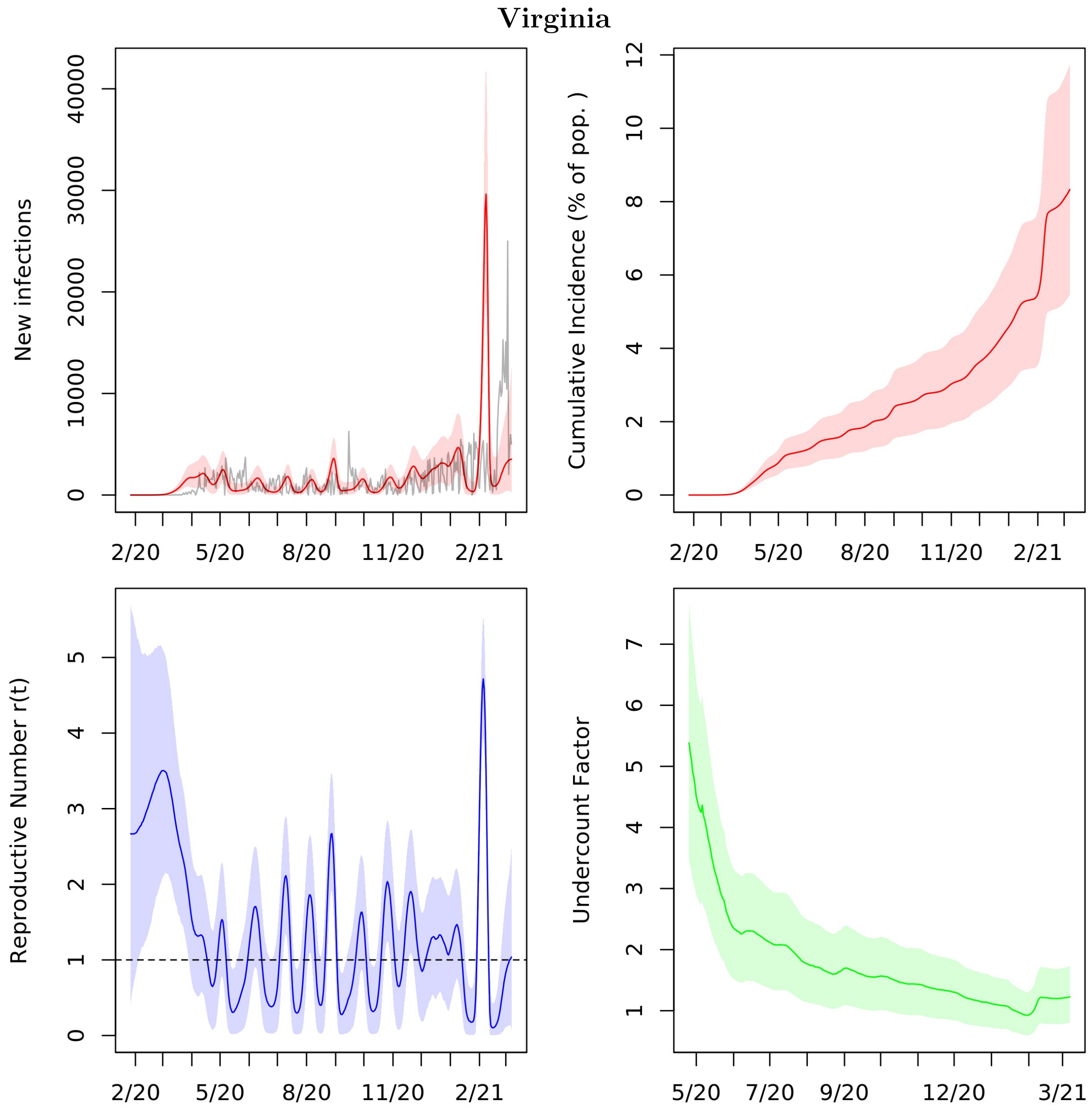

Figure 46: Posterior median and middle 95% intervals for daily new infections, cumulative incidence, $r(t)$, and cumulative undercount from March 2020 to March 2021. In the top left panel, deaths divided by the posterior median IFR are plotted in grey for comparison.

Vermont

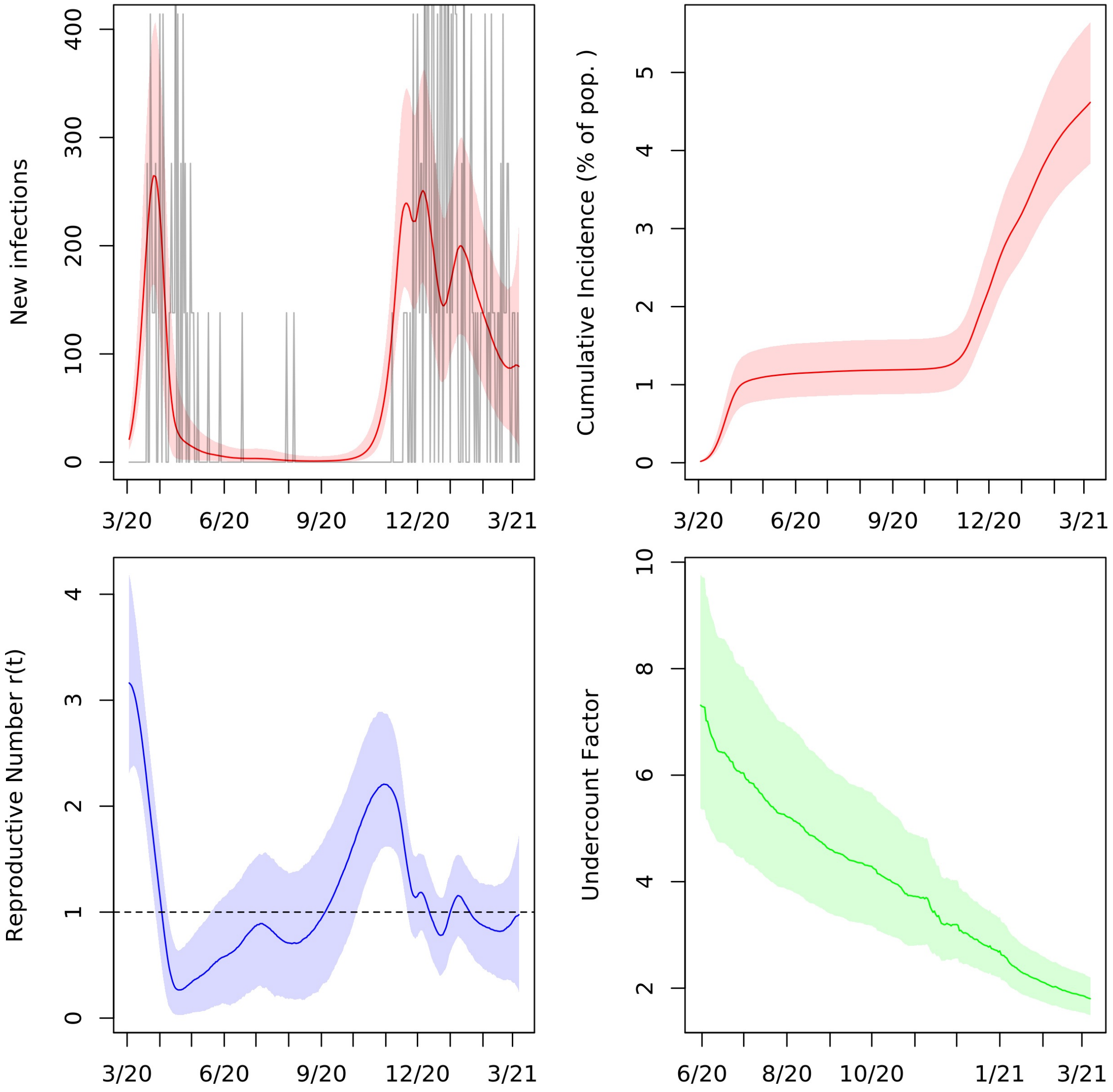

Figure 47: Posterior median and middle 95% intervals for daily new infections, cumulative incidence, $r(t)$, and cumulative undercount from March 2020 to March 2021. In the top left panel, deaths divided by the posterior median IFR are plotted in grey for comparison.

Washington

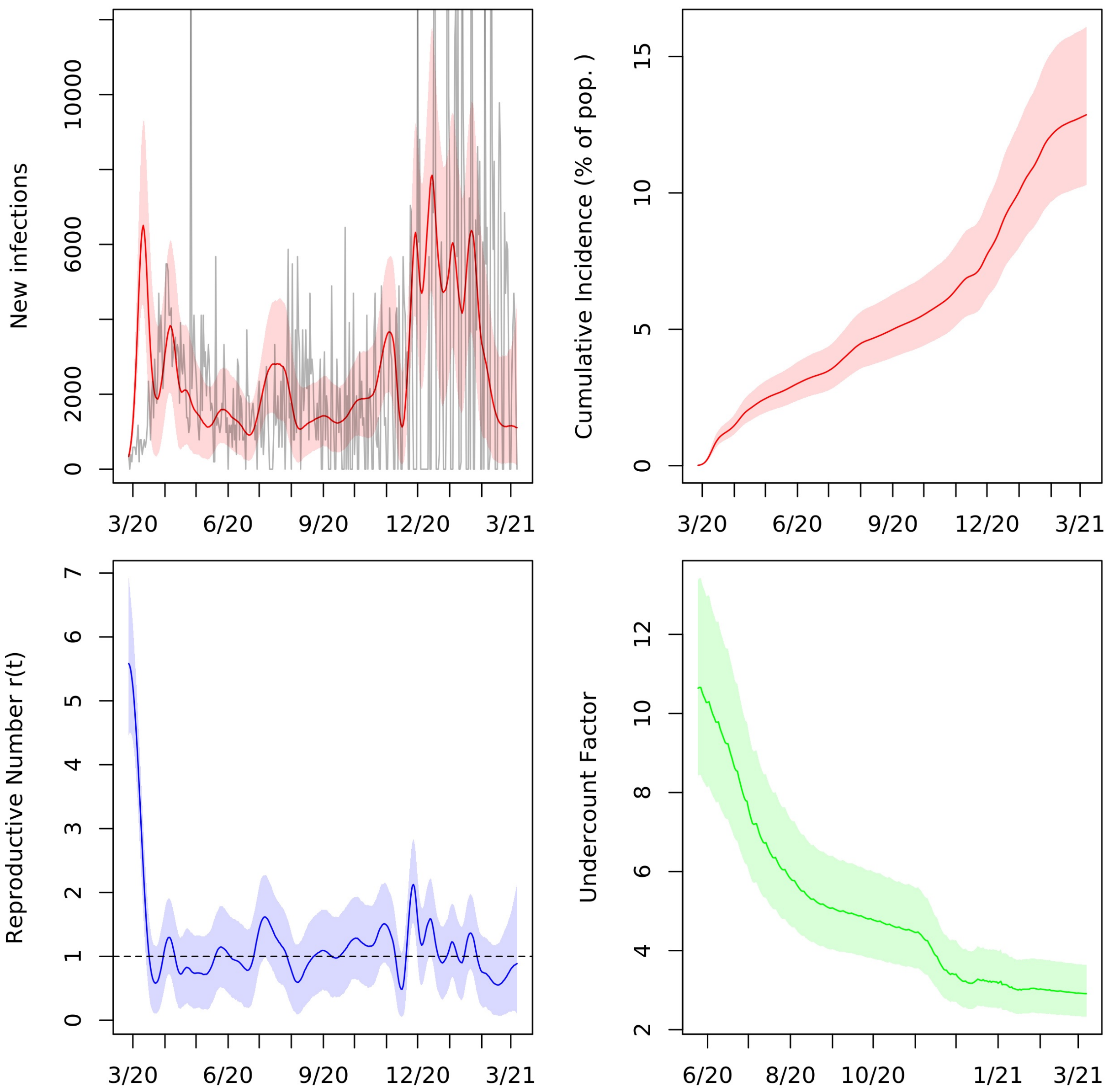

Figure 48: Posterior median and middle 95% intervals for daily new infections, cumulative incidence, $r(t)$, and cumulative undercount from March 2020 to March 2021. In the top left panel, deaths divided by the posterior median IFR are plotted in grey for comparison.

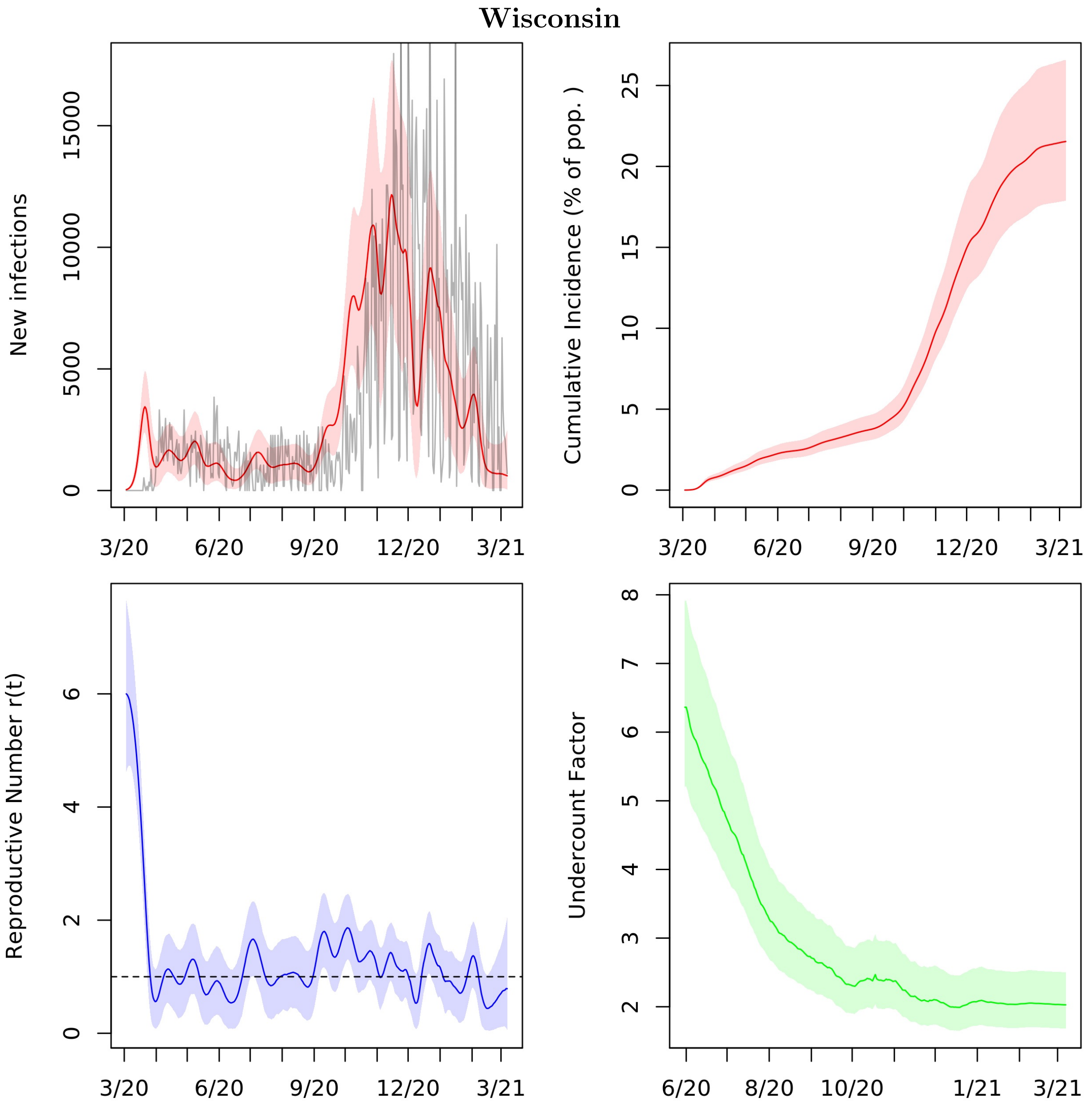

Figure 49: Posterior median and middle 95% intervals for daily new infections, cumulative incidence, $r(t)$, and cumulative undercount from March 2020 to March 2021. In the top left panel, deaths divided by the posterior median IFR are plotted in grey for comparison.

West Virginia

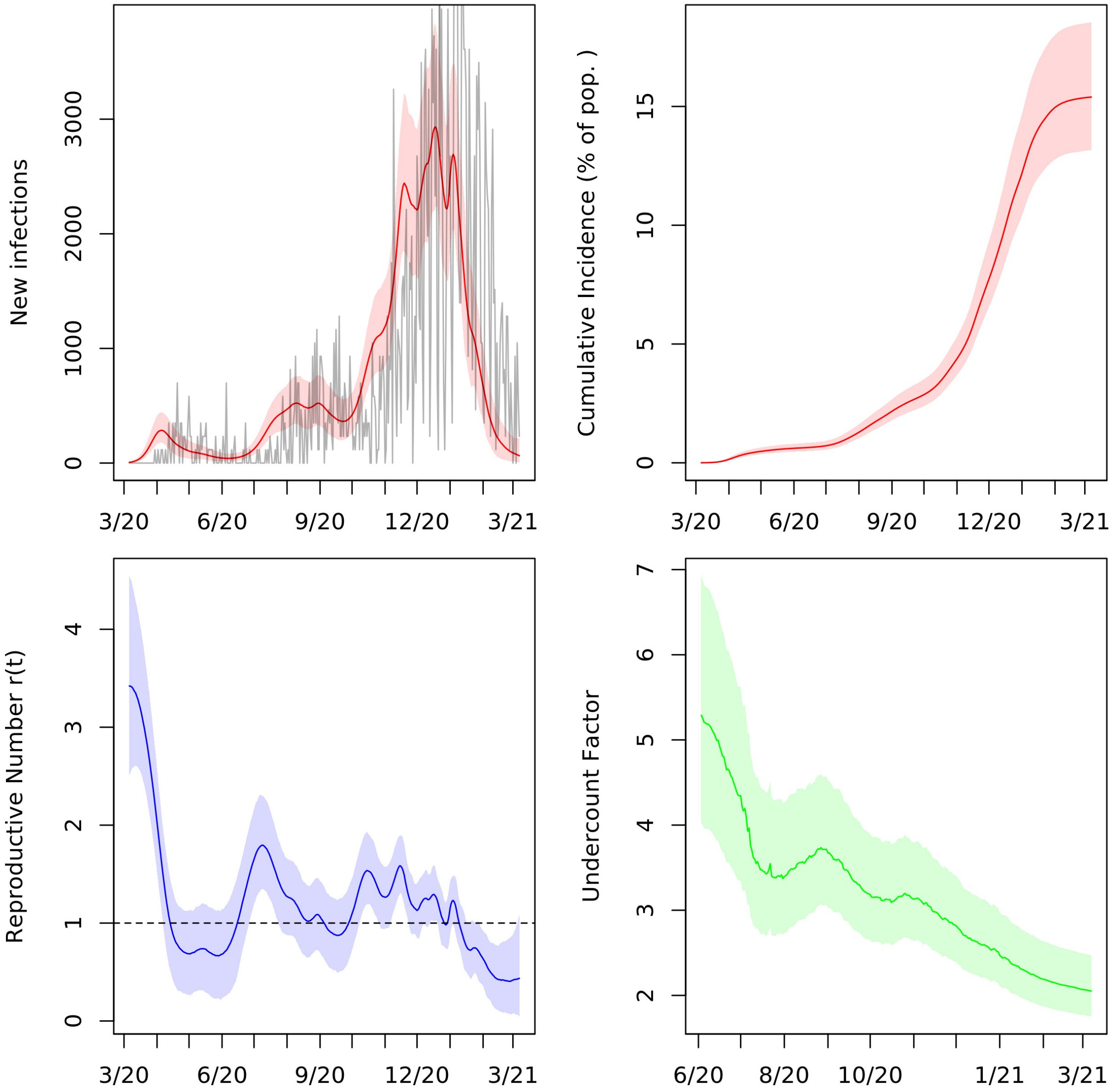

Figure 50: Posterior median and middle 95% intervals for daily new infections, cumulative incidence, $r(t)$, and cumulative undercount from March 2020 to March 2021. In the top left panel, deaths divided by the posterior median IFR are plotted in grey for comparison.

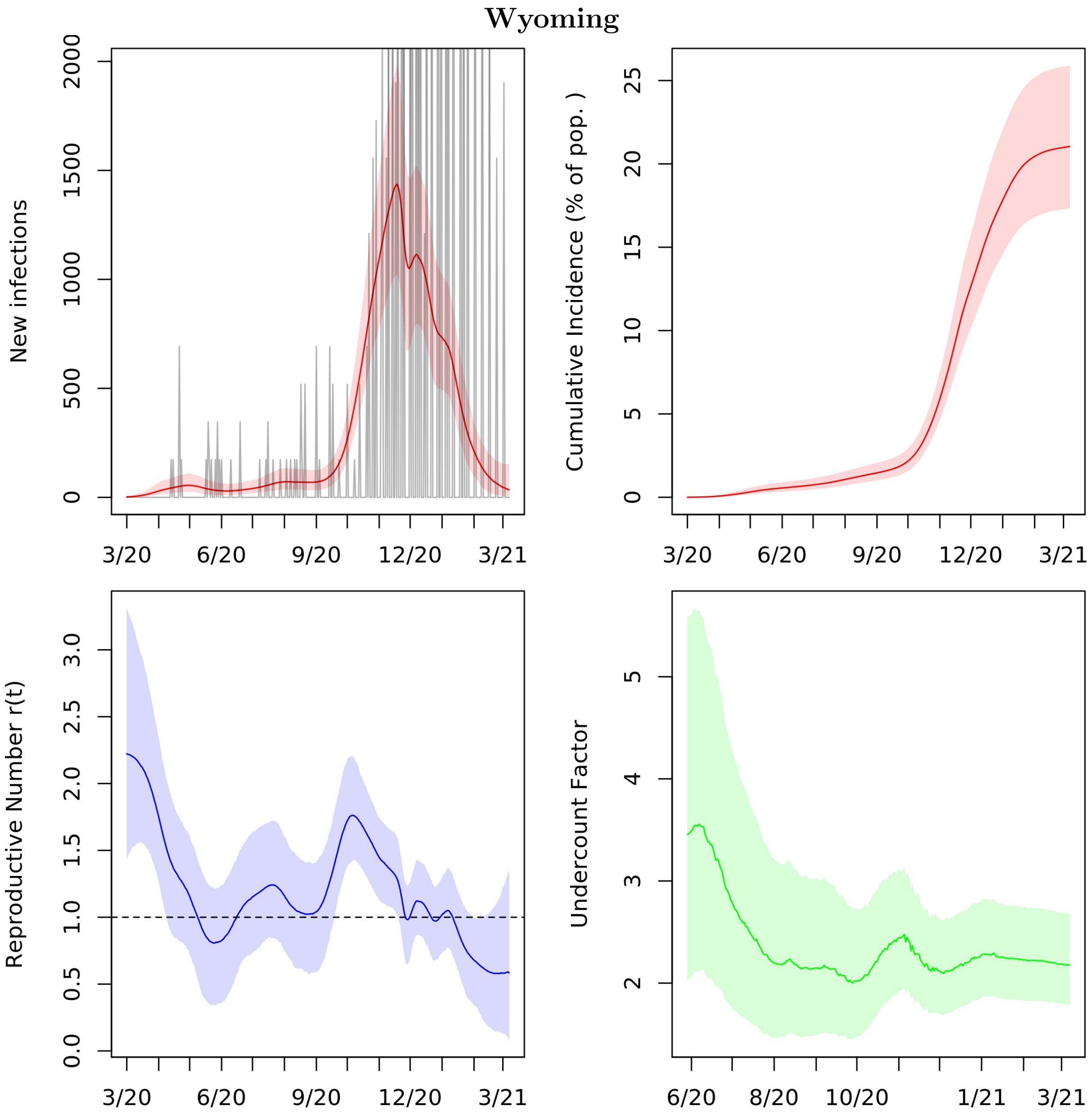

Figure 51: Posterior median and middle 95% intervals for daily new infections, cumulative incidence, $r(t)$, and cumulative undercount from March 2020 to March 2021. In the top left panel, deaths divided by the posterior median IFR are plotted in grey for comparison.
